# Supplementary material for: Genomic signatures of globally enhanced gene duplicate accumulation in the megadiverse higher Diptera fueling intralocus sexual conflict resolution
Source: PeerJ. 2020 Oct 12;8:e10012. doi: 10.7717/peerj.10012 (PMC7560327; doi:10.7717/peerj.10012)
Supplement: Supplemental Information 9 [file peerj-08-10012-s009.zip › Tim13 protein sequences 2020.docx]

>Dmel_Tim13_CG11611

MAAANMEKGELMNQVKQQIALANAQEMLSKMTEKCFKKCIQKPGKSLDST

EQRCISQCMDRFMDAWNLVSRTYGNRLQREQYRTMESLEMTS

>Dvir_Tim13_XP_002048624.1

MAQKQFGKGEVISQVRQQIAVANAQEMLSKMTEKCFKKCIYKPGKALDGTEQRCITQCMDRFLDTWNLVSRTYGNRLEREHLNAMRSENALN

>Dmel_CG34132

MAMANVDKGELMDQVKQQIAVANAQELLTQMTEKCFKKCVNKPGTSLDSSEQKCISMCMDRFMDSWNLISRVYGQRIQREQSKF

>Dvir_XP_002051868.1

MAHTVDKGEIMDQVKQQIAVANAQELLTQMTTKCFKKCINKPGTSLDASEQKCVSLCMDRFMDSWNLISRTYGQRLQREQSKM

>Dmel_CG42302

MATNQHDLERIRQQIVLANIQELIKKMTRRCFDVCIAMPEMELRSTERDCLANCMDRFMDSVQVVSSQYFRRRRRHQQIRLSRSTASSASPPASASASMPKSAAANESESASRASNDEKVK

>Dvir_XP_002059026.1

MRQQIALANAQQMLGKITVNCFRKCIDNPGKSLARAEERCLLQCMDRFMDSLKVVSLTYSRRLVRERK

>Dvir_XP_002055451.1

MERGSNIEPKELMSQLKQQIALANVQELLATVTCKCFEKCVTKPRDHLSGPEQSCIHLCMDRYLDSFRLCAHTYGHRLRREYSRQRS

>Ccap_XP_004523520

MSGITNLSSDKKGELIEQVKQQIAVANAQELLTKMTEKCFNKCISKPGLQLDSSEQKCISMCMDRFVDSYNLVSRTYSNRLQREQSKI

>Ccap_XP_004535304

MSEDIKTGELIEQVKQKIAVANAQELLTKVTEKCFKKCIVKPGTKLDTAEQKCISMCMDRYMDSWNLVSRTYGKRLQREQSKM

>Dant_Unigene1831

EKGELMDQVKQQIAVANAQELLTKMTEKCFNKCINKPGVSLDSSEQKCISMCMDRFMDSWNLVSRTYGNRLQREQ

>Tdal_comp157648

SGADKGELIEQVRQQIAVANAQELLTKMTEKCFKKCVGKPGVTLDSSEQKCISMCMDRFMDSWNLVSRAYGTRLQREQ

>Aaeg_AAEL008128

MEMSLDNLSSAQKDELMTSVKQQIALANAQELLTKMTEKCFKKCVGKPGQELDSSEQKCI

AMCMDRFMDSWNLVSRTYTQRIQKEQYKG

>Gmor_GMOY011701

MSDMLNLSNNEKGELIEQVKQQIAVANAQELLTKMTEKCFNKCIIKPGVQLDSSEQKCIS

MCMDRFMDSWNLVSRAYGSRIQREQNRM

>Gmor_GMOY006103

MSDLMTSTTSEKEEIIEEVKQQLAAANAQELLTKMTEKCFCKCITRPGAQLNSSEQKCIS

MCMDRFIDSWNLVSRAYGTRIQREQNRM

>Mdom_MDOA011431

MSGLSNLSSADKGELMEQVKQQIAVANAQELLTKMTEKCFNKCINKPGTSLDSSEQKCIS

MCMDRFMDSWNLVSRTYGSRLQREQHKM

>Cpip_CPIJ018211

MEMALDNLSSAQKDELMSQVKQQIALANAQELLTKMTEKCFKKCISKPGTELDSSEQDCK

RSMIFG

>Agam_AGAP002277

MDVSLENLSSSQKDELMTTIKQKIAIANAQELVTKMTEKCFKKCVGKPGQDLDGSEQKCI

AMCMDRFMDSWNVVSRALTQRLQQEQYKG

>Tcas_XP_008199565

MDSLGGGLTGAQKDELMDQVKQQIAVANAQELLTKMTEKCFKKCINKPGTSLDSSEQKCVAMCMDRYMDSWNLVSKAYSLRIQRERNNM

>Amel_XP_001121522

MSALTSESLTDKEKSEFMQQIKQEFAIASAQEMLSKMSEKCFKKCVVRPGTSLDSSEQKCVAMCMDRYMDAFNLVSKTYSARIQREHNRM

>Pcoq_MNCL01000148.1

DFMNGVKAKNNAASLAEFLNRMTPNCFKKCIEKPGTQLSPYEKGCIFKCTDLYLETWMVAARTYKKRLENE

>Pcoq_MNCL01000193.1

SVQKGELMDQVKQQIAVANVQELLTVSVEXKMTEKCFKKCVDKPGSVLDSSEQXKCIVMCMDRYMDSWNLVSRTYGSRLQRER

>Cnas_XP_031635707

MDLQAPLGNLSSTQKDQLMTEVKQQIAVANAQELVTKITEKCFKKCVTKPGTSLGGSEQKCIAMCMDRFM

DSWNLVSRTYVRRIQREQGGGGF

>Mdes_AEGA01014751

LDLASQISNLSPAQKDQLMNEVRQQYAVANAQELITXLLQKITEKCFRKCITKPGTSLGGSEQKCLAMCMDRFMDSFNLISKTYVRRIQREQKDQLMNEVRQQYAVANAQELIT

>Smos_VUAH01000001

MDLQAPLGNLSSTQKDQLMTEVRQQIAVANAQELVTXHLLQKITEKCFKKCVTKPGTSLGGSEQKCIAMCMDRFMDSWNLVSRTYVRRIQREQGGGGF
