# Supplementary material for: Genomic signatures of globally enhanced gene duplicate accumulation in the megadiverse higher Diptera fueling intralocus sexual conflict resolution
Source: PeerJ. 2020 Oct 12;8:e10012. doi: 10.7717/peerj.10012 (PMC7560327; doi:10.7717/peerj.10012)
Supplement: Supplemental Information 9 [file peerj-08-10012-s009.zip › Grx protein sequences 2020.docx]

>Dmel_Grx1_AAF46761

MGTVVSTLQRPTLYVSMDSSHAQFVRDTISGNKVVIFSKSYCPYCSMAKE

QFRKINVKATVIELDQRDDGNEIQAVLGEMTGSRTVPRCFIDGKFVGGGT

DVKRLYEQGILQKYFQ

>Dvir_Grx1_XP_002049720

MGTVVSSMQKPMMYVNMDSSQAQFVRQTIADNKVVIFSKTYCPYCSMAKEQFRKLNVQMTVVELDLRNDA

DEIQAVLGELTGARTVPRCFINGKFVGGGTDVKRLFEQGILQRYFQ

>Dmel_CG6852_AAF49222

MGAVGSALRSPIVDMSTKQAKFVENTIASNKVVIFSKTYCPYCTMAKEPF

KKLNVDATIIELDGNPDGNEIQAVLGEITGARTVPRVFIDGKFIGGGTDI

KRMFETGALQKYFQ

>Dvir_CG6852_XP_002047597

MDSAEAQFVRDTIAKNKVAIFSKTYCPYCTMAKEPFRKLKVNAMIVELDGRKDGNEIQSVLGEMTGARTV

PRVFINGKFVGGGTDIKRMYELGTLQKFFE

>Aaeg_AAEL013980

MGSFVS--RSPP--ANMSGPVAEFVKSAIAKDKVVIFSKTYCPYCTMAKEPFKKLNQPVACYELDQRNDGDEIQVVLGNLTGARTVPRVFINGNFVGGGTDIKKMYSDGRLEKLL

>Agam_AGAP011107

MGSLVSR----SVPANMSGPVAEFVKSAIAKDKVVIFSKTYCPYCTMAKEPFKKLNQEYACYELDKRNDGDEIQSVLGELTGARTVPRVFIGGNFVGGGTDIKKMYDDGRLQK

>Gmor_GMOY002543

MGSVISQLPKPEYTVDMNSPQAEMVKSAVQNHKVVIFSKSYCPFCTMAKEQFRKLDIAMHVIELDQRDDGDEIQSVLGEITGSRMVPRCFINGNFIGGGTDVKKMYDQGTLQKYF

>Gmor_GMOY011241

MGSVISQLPKPADTVNMSSPQAEMVKSALQNHKVVIFSKTYCPFCTMAKEQFRKLDIAIHVIELDQRDDGDEIQSILGEITGSRTVPRCFINGNFIGGGTDVKKMYDQGTLQKYF

>Mdom_MDOA002306

MGSVVSQLPKTAVNMSSPQAVFVQNALKEHKVVIFSKSYCPYCTMAKDQFKKLSVPFHVIELDQRQDTDEIQDVLGQMTGARTVPRCFIDGKFIGGGTDVKKMYETGALQKYF

>Ppap_PPATMP010765

VDMSGPVAQFVRETIASDKVVIFSKTYCPYCSMAKEVSVFGVFLGFPDHKFYWKFFQQFRKLSQDFTAIELESREDGSEIQDVLGEITGARTVPRVFVKGEFIGGGTDVKKLYNDGSLKK

>Tcas_XP_EFA08711.1

MGVFSSKLPIDMSSPKVEVVKDLIKSDTVVIFSKTYCPYCKLAKEVFNNLKKTFTTIELDKRDDGEEIQGILGELTGAKTVPRVFVKGQCLGGGSDVKALYDKGELQKYFD

>Tcas_XP_975253.1_PREDICTED_glutaredoxin-C3

MSSEKSKSKFVQNLIASDTVVIFSKTYCPYCQLTKEIFDDMDQKFTAIELDSRKDCEEIQEVLGQMTGARTVPRVFVNGSFLGGASDIKKLYENGQLQTYLD

>Amel_XP_001123018

MPTTKEEVNQLIASHSIVIFSKTSCPFCKMAKQVFHNLQKEYTAIELNERNDGDEIQSILGEMTGARTVPRVFVNGVCLGGGTDVKKLYETGELQKMF

>Rpro_RPRC009996-PA

LGSSSLPTDMSGPTAQFVKDAITQDKIVIFSKSYCPYCKMAKDVFDKLKKSYTSIELDGRDDGDQIQSVLNEITGARTVPRVFVNGECVGGGTDVKSLYENGQLEKML

>Dant_Unigene1609_transcribed_RNA_sequence_GAWI01006090

MGSVVSQLPKQAVAINMSSPQAQLVQNVLKEHKVVIFSKSYCPYCTMAKDQFKKLSVEAF VVELDKRDDGDEIQSVLGQITGGKTVPRCFIDGEFIGGGTDVKQMYEKGTLQKYF

>Tdal_comp160147_transcribedRNAsequence_GBBP01087487

MGSNISTLLKNPVVDMTSPKADLVQHAIKSNKVVIFSKTYCPYCTMAKEQFEKLSVSPTVIELDERNDADEIQNILGEITGSRTVPRCFIDGKFIGGGTDVKKMYEKGSLQKYF

>Ccap_XP_004519116

MGSVISALPRKPPISVNMSSPQADMIREIIANNKVVIFSKSYCPYCTMAKEQFRKLSVAAHVVELDGRNDADEIQNILGELTGGRTVPRCFIDGKFIGGGTDVKKMYEQGTLQKYFT

>Pcoq_MNCL01000001

MGTIIDRMSGPRIDMTNAEAKFVVETIQKDKVVIFSKTHCPYCIMAKEYYSIVFQQFDKLQHPYVAIELDRRNDANEIQDVLMQMTGARTVVPRVFINGKFVGGGTDIKKMYDNGQLKK

>Mdes_AEGA01023598

FVNEAIAGSKVVIFSKTYCPYCTMAKEVSQHIVTCFVIRHSQIYKKINHFFLLQEFRAIELDSRDDGDDIQAALGELTGARTVXVPRVFINGKFIGGGTDVKTMRETGELSKLLN

>Cnas_VYII01002135

EFVTKAIAQNRVVVFSKTNCPNCNTVKQQLEYLVTKKGFKTIEMDQLANGKQIQDALELLTAIPTVPYVFVNGKFIAGETDLKDMHQSGQLQK

>Smos_VUAH01006165

AEFVSETIAKDKVVIFSKTYCPYCTMAKEXQFQKLKQEFTAIELENRDDCNEIQAALGQITGGTTVPRVFVKGKFIGGGTDVKKLNESG

>Cnas_VYII01000127

MGNILNTARPNPTNIAMAS---KFVSETIASNKVVIFSKSYCPYCTMAKEVRRNIFVRKLYFHLXQFQKLQQEFTAIELDKRDDGGDVQAALGEITGATTVXVPRVFVNGNFIGGGTDVKKLNQTGELKKLLE
