# Supplementary material for: Genomic signatures of globally enhanced gene duplicate accumulation in the megadiverse higher Diptera fueling intralocus sexual conflict resolution
Source: PeerJ. 2020 Oct 12;8:e10012. doi: 10.7717/peerj.10012 (PMC7560327; doi:10.7717/peerj.10012)
Supplement: Supplemental Information 9 [file peerj-08-10012-s009.zip › Trx protein sequences 2020.docx]

>Dmel_Thioredoxin-2

MVYQVKDKADLDGQLTKASGKLVVLDFFATWCGPCKMISPKLVELSTQFADNVVVLKVDVDECEDIAMEYNISSMPTFVFLKNGVKVEEFAGANAKRLEDVIKANI

>Dmel_deadhead_Trx-1

MASVRTMNDYHKRIEAADDKLIVLDFYATWCGPCKEMESTVKSLARKYSSKAVVLKIDVDKFEELTERYKVRSMPTFVFLRQNRRLASFAGADEHKLTNMMAKLVKA

>Dmel_CG13473

MAAMQKKVIIVDSKSYFDKLIDDAGTNKYVLVEFFATWCGPCAMIGPRLEQLASDYFGRMLVLKIDVDENEDLAVQYEVNSMPTFLIIKNRVTLIQFVGGNVERVVSTVEKFVGKVEDSKEHKSKEGGASSATVPKLER

>Dmel_thioredoxin_T

MVYPVRNKDDLDQQLILAEDKLVVIDFYADWCGPCKIIAPKLDELAQQYSDRVVVLKVNVDENEDITVEYNVNSMPTFVFIKGGNVLELFVGCNSDKLAKLMEKHAGVYTDEAADVKAVHIDGECIVDLTAESSESDNDNNNVNEVSAHDENAVLEH

>Ccap_Contig5575

MMHTVRSNADFDRQLMAAGGRLVVVDFTASWCGPCKSIEPKVKALSRKYKDRAVVLKVDVDKCSNVAHDYRVSCMPTFVFIRNGRRIDRFSGADEMELE

>Mdom_MDOA014337

MIYNIQDKKDLEKHLANAGGNLVVLDFYATWCGPCKLIAPQLEEISTSHSDVVVVLKINV

DDCEEIAMDYNVTSMPTFVFMKNRKIIDVIVGGNSENLTKNVEKYIHHENVNNANTQAID

NADGE

>Dvir_XP_002057022

MVHVIQSKEDFEQQLSNAGDKLIVIDFCANWCGPCKIIAPKLEELAATYAERAVVLKVNVDENEEITIEYNITSMPTFVFIKSGEVLEVFVGGNSDKLAKSMEKYVSDSDEPNEEMQQQLSTSSESAASGSTICDLDEEVEGAHEMSMRVQSGALDKEGVLEN

>Ccap_comp62603_c0_seq1.2

MVYTIQNKEDLDKKLEEAVGSGQLVVIDFFANWCGPCKIISPKLEELATQYAEKAIVLKVNVDDCEEIALEYNVTSMPTFVFIKDNHVIDVFVGGNSEKL

>Dvir_dhd_XP_002057023

MSAIRSVNDFYKRIEAAENKVVLVDFYATWCGPCKEMDPMVKSLARQYASKAIVIKVNVDKFDELVEKYK

VRSMPTFVFLKGNRSLGKIIGADEHKLTRMMAKVCK

>Dvir_XP_002057025

MPTVRTVSGFNKRLEAAKNKVVVLDFYARNSKPCQDIDQLVQTLEYKYASKAIIIKVNVDKFDELVDEYRVRSMPTFVFLKCNHNVAKIIGADEHKLTRMMAEMCETE

>Mdom_MDOA000488

MHLSQILQGINTVPIYYFFSDLFWSTNLTMVSIIKNTEDFEKKLANAGDKLVILDFYATW

CGPCKEMDPHIRKLTQKYKDQAIVLKINVDKFNEISDYYKVKSMPTFVFIKNKKRLSSFA

GADDEMLQQRVEQYVN

>Dant_Unigene1032

VSIIKTTEDFQTKLDTAGDKLVLLVFYATWCGPCKEMDPHVRQLIKEYKDRAMAIKINVDKFEDICDYYKIRSMPTYVFIRNKKRLSSFAGADAK

>Dvir_XP_002047895

MASAATNKNKPKKVITIQSKEDFDRIVAEAGNKHVLVEFFATWCGPCALIGPRLEELAYEYEDCLLIVKV

DVDDHGDLAEEYDVSSMPSFLIIKNKVKLEQFVGSNGDKVQSTLQKFCGKPDDKKAPVAGPSAQPTQKTN

RLVSMLKAVTPSKSNNMPTDKQ

>Dvir_XP_002057775

MVYAVKDVNDFKTQLTNAGDKLVVVDFFATWCGPCKMIAPKLEELSQQYAEKIVVIKVDVDECEDIAMEY

NISSMPTFLFIKNSVKVEEFAGANAGRLAETIQKLI

>Tcas_XP_967987

MVTHINDKADLVSKLTDAGDQLVVIDFFATWCGPCKMISPKLEELAQEFQNVHIFKVDVDECEDIAMEYNISSMPTFVFVKNSQTITQFSGANYEKLRQLVVENK

>Amel_XP_003250408

MVYQIKNASDLKNQLEKAGNQLVVIDFFAMWCGPCKMIGPKVEELSMEMEDVIFLKVDVDECEDIAGEYEITSMPTFVFIKNNKVLENFSGANYDKLKSTIQKHK

>Aaeg_AAEL010777

MVYIVKDAADFDSKLESAGDKLVVVDFFATWCGPCKVIAPKLEEFQNKYAEKVLIIKVDV

DECEDLAAKYEISSMPTFLFIKGKKVVYQFSGANDQKLEMYILKHA

>Agam_AGAP009584

MVYMVKDSEDFNNKLEAAGDQLVVVDFFATWCGPCKVIAPKLEEFQNKYADKIVVVKVDV

DECEELAAQYNIASMPTFLFIKRKEVVGQFSGANAEKLENFIQQHSA

>Cpip_CPIJ012011

MVYVVKDAADFDSRLEAAGEKLVVVDFFATWCGPCKVIAPKLDEFQNKFSEKIVIIKVDV

DECEDLAAKYNISSMPTFLFIKNKEVVDQFAGANAEKLQSFITKHSE

>Gmor_GMOY006832

MVYAVRNKADFNQQLENAGNKLVIVDFFAHWCGPCKMIAPVLEELSKEYADKVVVIKVDVDECEDVAMEFNISSMPTFVFIKNKQTLEEFAGANAEKLEATILNLV

>Gmor_Y1scf7180000649373_1

VHTIGDKEDFENILTAAGDKYIMVEFFATWCGPCRMLGCKIDELASLYQDKAIMVKIDVDDFEDLAAEYDITSMPAFMIIKNKQKVEHYCGSKVEQLEEFIEKHLG

>Gmor_Y1scf7180000649373_2

MTYSVQSKEDMEKQINEAGDKLIVIDFYASWCGPCKIISPKLEELSVQYSDKALVLKVNVDDCEEIALEYNVTSMPTFVFMKNRQIIDIFVGGNPEKLVKNMEKYVG

>Mdom_MDOA007400

MVYTVTSKSDFDQQLENAGDKLVVVDFFATWCGPCKMIAPRLQELSNEYADKIVVIKVDV

DECEDVAMEYNISSMPTFLFIKKKQELEKFAGANAEKLLATIQKHA

>Mdom_MDOA004695

MVEFFATWCGPCRMLGCKIDELASLYQDKAIMVKIDVDDFEDLAAEYDITSMPAFMIIKMVEFFAPWCPACKNLAPTWERFATTAADINVNVAKIDVTTSPSLSGRFFVTALPTIYHVKDGEFRQYRGSR

>Mdom_AQPM01000161

TIDSKDDFENIIKEAGERLIMFEFFAPWCGPCRILTTKLVDMANLYRDKLLIVKIDVDEFEDLAIEHNVTAMPTFLIMQNKKLLQQFSSSNAEHLQETVEKYAG

>Llon_LLOTMP005819

MVHLVADTADFDGKLDSAGDLLVVVDFFALWCGPCKMIAPKLEELANQYSEKAVVLKVDV

DECEELAMRYNISSMPTFLFIKNKEVVESFSGASGEKLASFFAKYTA

>Ppap_PPATMP003111

MVHVVVDTMISTFNVVKVTAVRFSWNFSGQADFDGKLESAGDLLVVVDFFAQWCGPCKMI

APKLEELATQYTDKAVVLKVDVDECEELAMRYNISSMPTFVFIKNKQVVETFSGANNDKL

ANFFVKYTA

>Ccap_comp61885_c0_seq2

MPHLVKDLSDLNNQLKEAGEKLVVIDFFATWCGPCKIIAPTLDELAKEYEGRIVVLKVDVDECEDIAMQYNVSSMPTFVFIKQTKQISSFSGASAKKLAEDI

>Ccap_Contig5575_2

ISDRGTYDQLMNAIGRKHLLIEFYAPWCGACMLINKKLEELAVTYTGKLIIAKVNIDDCEQIAVENNVSMMPAFILFKENQILEKFAGSNEEKLMSTIKKHV

>Tdal_comp151633

MVYVVKNRQDFNMKMAEAGNKLVVLDFCATWCGPCRIISPKVEELAGTYAAKAVVLKINVDECEELAMEYNVTSMPTFIFIKNGIVVDSFVGGNAEKL

>Tdal_comp155821_1

IMSINSKELFDKALASAGNKIVMVEFFAEWCGPCKKITPKLEELSTQYAERLIMLKVDVDDCEDIAIQYKVTSMPTFIFLKNNEKVEEVIGSNTDKIEKAL

>Tdal_comp155821_2

MAYIVKDKFDLQQKLIEAGDKLVVLAFYANWCGLCSIISPKVNELANNYAIQAAILKINVDQSRDIATDFNVTTLPTFIFMKHRRIIDFSTSGNTDELNKYI

>Tdal_comp144014

MVYIVKNKTDFDQQLEDAGDKLVVVDFFAVWCGPCKMISPKLEELAKEHSEKVVVIKVDVDECEEIAMEYNVSSMPTFLFIKNKQVKEQFAGANAEKLASYIE

>Tdal_comp4265346

MVHKVVDRADFNKQLTDAGNKLVVVDFFATWCGPCKLISPQLDKLAQQYTNIVVLKVDVDENEEVATSYNVNAMPTFV

>Tdal_comp146431

MLYIVQSYNDFNEKLKEAGSKLVVVNFFANWDSACRLISPSLRVLANTYGKDAVLLKINVDRLTDLCTFYDVTMVPTYIFLKNKCNIDTAVGPSLKELEALIK

>Tdal_comp166262

MVYTISSDSDFEKKLIEAGSKLVLVDFYAKWCVPCMNISPSITTLSRRYSKNVVVLKVDVDKLTNLTTYYKIVSMPTFIYIKNKRVVGKVIGADFKGIEN

>Tdal_comp151557

MVHNIEGAVDYNKQLNEAGSKLILLQFYSTICSRCLYLSSSIDELSKKYNENLVVLLINVDTLDNLSRHFKIKATPTFLYIRNKRMVGKLVRADLNEIE

>Dant_Unigene1698

KMVYAVKSKSDFDQQLENAGDKLVVVDFFATWCGPCKMIAPKLEELSKEYVEKILVIKVDVDECEDVAMEYNISSMPTFIFIKNKQKVEEFAGANAEKLAATISKL

>Dant_Unigene22236

SSKLVVVDFFATWCGPCKMLAPLFERFAAEYTDAEFYKVDVDELGAVAQEQEVSAMPTIIFYKNGKVVDKVIGAN

>Pcoq_MNCL01000044

DFDDRVNGAGDKLIVVDFFATWCGPCKVIAPKLEELATKYDDKVIVLKVQVDVDDCEEIAMEYKIQSMPTFIFIKNGQEIDRFSGANAENLEKNFAKYAQ

>Cnas_XP_031625960

MVHLVKDVADFDEQVNNAGDKVVVVDFFATWCGPCKVISPVLEKFAQQYASNIVVLKVDVDECEELAMRFDVSSMPTFVF

MKDGKKVDSFSGANPDKLEKTIVQYIN

>Cnas_XP_031625959

MCLILSCFCVGHTRTNSWLEIGSMAQFDYEMRNAIDRLIVVEFYYSWCDYSKLIVSSLDLLALKYSNVIMLRVDIDKFKALAARYNVIYSPTFVYLRFSRLLEIFTDANVNRFEHVLNKRLRYL

>Smos_VUAH01000001_1

FSPN-SFQADFDEQIKNAGDKLVVVDFFATWCGPCKVIAPVLEKLAQQYASNLVVVKVICVDVDECEDLAMRFDISSMPTFVFLKKGEKVEVFSGANPDKLEKTIVQY

>Smos_VUAH01000001_2_3

QAHFDEQVKNAGNVLIVVEFFAQWCGPCKVITPALNKLAQKY-DNVIMLKVXFQMNVDENIELAARFKVTSMPTFVFLRNSETQEYFAGADLRRVEKTIVKF

>Smos_VUAH01000001_6

QPQFDHEIRNAGNKLVVIEFYYSWCGHTQKITPDLEQMSHKYPNVVMLRVFQVNIMNFSELAERYKIIYSPTFVYLRHGRLLEMFADA

>Smos_VUAH01000001_5

VDIEKMKDLGTRFKISAVPAYVFLKDGQFIESFLGAESRRLEHTIMKXIKLTIYYRSQAQFDEQLKNAGNGLVVVEFCVKWATPCIAMKTILEKLAVLYENVAI

>Smos_VUAH01000001_7_8

VDIEEMKDLGTRFNISAVPAYVFLKDGEFIESFLGFQPDHLXYHSQAQFDEQLKNAENGLVVVEFCVKWASPC

>Mdes_gb_AEGA01013763

FDEQVKEAGEKLVVVDFYATWCGPCKVIAPALEKFSQQYAANIVVLKVXDVDECEELAMRFEISSMPTFIFLKNGEKVDGFSGANQDRLEKTIVQYIN
