# Supplementary material for: Genomic signatures of globally enhanced gene duplicate accumulation in the megadiverse higher Diptera fueling intralocus sexual conflict resolution
Source: PeerJ. 2020 Oct 12;8:e10012. doi: 10.7717/peerj.10012 (PMC7560327; doi:10.7717/peerj.10012)
Supplement: Supplemental Information 9 [file peerj-08-10012-s009.zip › Tom40 protein sequences 2020.docx]

>Dmel_Tom40_AAF46272

MGNVLAASSGAPGSGASNLGLGLQEPAPLPSNSGSLTESSSSAEGLDSLA

AAKDAALENPGTVEELHKKCKDIQAITFEGAKIMLNKGLSNHFQVSHTIN

MSNVVPSGYRFGATYVGTKEFSPTEAFPVLLGDIDPAGNLNANVIHQFSA

RLRCKFASQIQESKVVASQLTTDYRGSDYTLSLTVANPSIFTNSGVVVGQ

YLQSVTPALALGSELAYQFGPNVPGRQIAIMSVVGRYTAGSSVWSGTLGQ

SGLHVCYYQKASDQLQIGAEVETSLRMQESVATLAYQIDLPKANLVFRGG

IDSNWQIFGVLEKRLAPLPFTLALSGRMNHVKNNFRLGCGLMIG

>Dvir_XP_002058101.1

MGNVLAASSGLDTTAARSNSVAASLGLPEPAAIPTEPTPSATIAADASVAAKDHQLENPGTVEELHKKCK

DVQAITFEGAKIMLNKGLSNHFQVSHTINMSTTVPSGYRFGATYVGTKQYSPTEAFPVLLGDIDPSGNLN

ANVIHQFSSRLRCKFASQIQESKMMATQLSTDYRGNDFTASITLGNPSIFTNSGVVVGQYLQSVTQRMAL

GAEVAYQYGPNVPGRQIAIVSALGRYSNGDSIWSGTLGPSGLHLCYYQKASEQLQIGVEVETSLRMQESV

ATIAYQIDLPKADLVFRGSIDSNWHISGVLEKRLQPLPFSFALSGRMNHVKNNFKLGCGLMIG

>Dvir_XP_002057312.1

MGNVHTLEEGKVHGHGHGRGQGQGRGQGKGYEKEKLYRFFGIRMANEGDGYSITGQRRAEIQMKQEENLE

TAGPGGTRSAAAVAVGGLAVPATALPERLGNPGTVAELHRRCHDMLPQTFDGFRLNVSRAL GSNVTVGHS

LQVGNRPQTADCQFNASYMGQRQHELTGEAYPMIIGEVDAHGNVTGTLMHFITPRCRGKLTTTILDSEVQ

SSRLFLDYFGNNYSCTCVLSNIDLAQRMGVFVASYLQQVTPELALGVDYIYQREDIVPGGQAALVSAVAR

YQQENRQWSAMLSLHALELCYTQFYGQSLGASVQLQANILKRQAISRLCYHCHMPRVGFSFRGGIDTRGV

ISAVCEKRLEPLPILLQLSGKLNHLTSRFRFGLGLTLG

>Dmel_tomboy40_AAF57268

MGNVMASTADAESSRGRGHLSAGLRLPEAPQYSGGVPPQMVEALKAEAKK

PELTNPGTLEELHSRCRDIQANTFEGAKIMVNKGLSNHFQVTHTINMNSA

GPSGYRFGATYVGTKQYGPTEAFPVLLGEIDPMGNLNANVIHQLTSRLRC

KFASQFQDSKLVGTQLTGDYRGRDYTLTLTMGNPGFFTSSGVFVCQYLQS

VTKRLALGSEFAYHYGPNVPGRQVAVLSAVGRYAFGDTVWSCTLGPAGFH

LSYYQKASDQLQIGVEVETNIRQQESTATVAYQIDLPKADLVFRGSLDSN

WLISGVLEKRLQPLPFSLAISGRMNHQKNSFRLGCGLMIG

>Ccap_XP_004523968.1

MGNVLAASSSSSATLDAAKLGLPELSTPSQNQSASSAAAAMPSAATGIDSKEGKLDNPGTVEELHKKCKDVQAMTFEGAKVMLNKGLSNHFQVSHTLNLCTAQPSGYRFGATYVGTKQFGPSEAFPVLLGDIDPSGNLNANVIHQFSPRIRCKFASQVQDSKLTAAQLTTDYRGEDYTASLTVGNPNIFNNSGVFVGHYLQSITDRIALGAELAYQYGPNVPGGQIAVVSAVGRYSEGNSLWSGTLGPGGIHLCYYQKASDQLQMGVEVETSLRMQESVATFAYQVDLPKADLIYRGSLDTNWNVCGVLEKRLQPLPFSFALSGRINHTKNQFRLGCGLIIG

>Dant_Unigene2702

DNPGTVEELHKKCKDIQAMTFEGAKVMLNKGLSNHFQVSHTLNLCSTQPSGYRFGATYVGTKQFSPSEAFPVLLGDIDPSGNLNANVIHQFTPRIRCKFASQIQESKMTAAQLTTDYRGNDFTASLTVGNPNIFNNSGVFVGHYLQSVTQKIALGAELAYQYGPNVPGGQIAVLSAVGRYTSGDSIWSGTLGPSGVHVCYYQKASDQLQIGVEVETSLRMQESVATIGYQVDLPKADVIFRGSFDSNWNVCGVLEKRLQPLPFSFALSGRLNHTKNQFRLGCGLIIG

>Tdal_Td_comp157445

ENPGTVDEIHKKCKDVQALTFEGARIMLNKGLSNHFQVSHTLNMSSTQPSGYRFGATYVGTKQFTPSEAFPVLLGDIDPSGNLNANIIHQFTPQLRCKFAMQIQESKVLACQAAGDFKGQDFTASVTLGNPNIINGSGVIVAHYLQSITKRLALGTELAYQYGGNVPGGQIAVVSAIGRYATENYVWSGTAGPAGVHICYYQKASDQLQIGAEVETSPRMQESVATIAYQVDIPKADLIFRGSFDSNWNVAGVLEKRLTPLPFSFALSGRLNHTKNQFRLGCGLIIG

>Aaeg_AAEL007001

EKPLENPGSMEELHKKCKDVMPANFEGAKLMINKGLSNHFQVSHTINLNSSNTSGYRFGATYVGTKQMSPSEAFPVILGDIDPAGNLNANIIHQLTPNVRCKFASQIQNQKVTAAQLTTDYKGQDFTASLTVGNPNILNNSGVMVAHYLQAVTNKLALGGELAYQYGPQVPGGQIAIMSAAARYATEVGTWSGTIGLAGLHICYYQKASDQLQLGVEVETNLRMQEATATMGYQIDLPKSELVFRGMVDTNWTVAAVLEKKLQPLPFTFALSGILNHTKNQFRLGCGLIIG

>Llon_LLOTMP006852

GTLEDLHKKCKEVFPATFEGGKVMINKGLSNHFQVSHTLTLSGDPGKTGISGYRFGATFVGTKQMSPTEMYPVLLGDIDPAGNLNANIIHQLTDRIRGKFAAQIQDSQVSAAQMSAEYHGHDFTASVITANPNLVNGSGIIIGHYLQGITDNISLGSEIVYQFGPAVPGGENAMISLAGRLIRGDTTWTGTVDFSGVHLCYHSRASEQLQLAVEVETNFRMQDSVATFGYQVDLPKANLVFKGMVDTHGTVAGVLEKKLEPLPFTLALSGALNHSKNQFRMGCGIVLG

>Gmor_GMOY009558

MGNVMAATSSTPSSAATALDKAAKQGLPEPAPPGALTSQSKEKDDSLENPGTVEELHKKCKDIQAMTFEGAKVMLNKGLSNHFQVSHTLNLCSTQPSGYRFGATYVGTKQFSPSEAFPVLLGDIDPSGNLNANIIHQFSPRIRCKFASQIQESKITAAQLTTDYRGDDYTASLTVGNPNIFNNSGVFVGHYLQSITKCMALGAELAYQYGPNVPGGQIAVVSAVGRYANGDSTWSGTLGPGGVHICYYQKASEQLQIGVELETSLRMQESVATMGYQVDLPKADLIFRGSFDSNWNVCGVLEKRLQPLPFSFALSGRLNHTKNQFRLGCGLIIG

>Ppap_Contig4257

GTLEDLHKKCKEVFPATFEGGKVMINKGLSNHFQVSHTLTLSGDPGKTGVSGYRFGATFGTKQISPTEMYPVLLGDIDPAGNLNANVIHQLTDRIKGKFAAQIQESQVTAAQMSAEYHGHDFTASVITANPNLVNGSGILIGHYLQGITDNISLGSEIVYQFGPAVPGGENAMISLAGRYIHGDTTWTGTIDFSGVHLCYHSRASEQLQLAVEVETNFRMQDSVASFGYQVDLPKANLVFKGMVDTHGTVAGVLEKKLEPLPFTLALSGALNHSKNQFRMGCGIVLG

>Agam_AGAP007871PA

LENPGTMEELHRKCKDVMPVNFEGAKLMINKGLSNHFQVSHTINLNSSNTSGYRFGAMYVGTKQLSPTEAFPVVLGDIDPSGNLNANIIHQLTPKVRCKFASQIQSSKVTAAQLTTDYKGPNYTTSLTIGNPNIINNSGVVVAHYLQAVTNKLAMGGEFAYQYGPAVPGGQIALMSAAARYATDLYTWSGTVGAAGVHLCYYQRASEQLQIGAEVETSFRMQESVATIGYQVDLPKSDLVFRGMLDSNWTVGAVLEKKLQPLPFTFALSGMLNHTKNQFRLGCGLIVG

>Cqui_CPIJ002542PA

AEPEQPPLTNPGTMDELHKKCKDVMPANFEGAKLMINKGLSNHFQVSHTINLNSSNTSGYRFGATYVGTKQTSPTEAFPVILGDIDPAGNLNANIIHQLTPFVRCKFASQIQNSKITAAQLATDFKGDDYTASITVGNPNIINESGVIVSHYLQAVTNRLTLGGELAYQYGPAVPGGQIAIMSAAARYATELSTWSGTIGVAGVHLCYYQRASEQLQLGVEVEANLRMQEAVATIGYQIDLPKSELVFRGMVDTNWTVAAVLEKKLQPLPFTFALSGILNHTKNQFRLGCGLIIG

>Tcas_XP_966771

MGNVHAYSAPAPPPPPPSAAPYTKPETPTSGPPQEVENPGPLEEIHTKCKNIFPTNFEGARVMLTRGLSN

HFQISHTINMSSITPSGYRFGATYVGTKQISPSEAYPILLGDIDPSGNLNATIIHQLCSKVQAKFGAQVQ

NSKFTVGQLTMNYKGSDYTASVTVANPDIISGSGVMVLHYLQAVTPRLALGSELAYQKGPAIPGGEIALL

SAAAKYTTENTQISGTLGVSGVHLCYYQKASSQLQIGVELEANPRMQESVASIGYQVDLPKSEVVFKGHV

DSNWSVGAVLEKKLSPLPFTLALSGLLNHNKNQFRLGVGILIG

>Amel_XP_391836

MGNVLAASVPPPPSPPPPTSGLLPNLEKPDSSETLNSSSRLSDGLKNPGTIEDLHKKCKDVFPANFEGAK

LMFNKGLSNHFQISHTISMSSIAQSGYRFGATYVGTQQPVPSEAYPVLLGDIDPSGNLNANIIHQFGERL

RGKLATQVQKSKFTAVQMTTDYRGDAYTVSLTLGNPDILNGSGVFVMHYLQSITPSVALGGELAYQRGPA

VPGGQVAVLSAAGRYTNGDSTISGSLGLSGCHLCFHQKASQQLQVGVELEINCRIQESTGAIAYQIDLPK

ADLIFRGSVDTNWTVGAVLEKKLQPLPFTFALSGMINHSKPQFRLGCGLIIG

>Amel_XP_006567371

KLEEPSVISNEDCIPCEAEDRAPGNPGSFEDIHKKVKDLYPQNFEGARLIINKILSQHFNV

THTITLSSVTPSGYKFGAKYIGTKVVNPNERYPVASGDIAPNGNLTASFMHTLGCRLRYKLSAQIADGKC

KASSSSLEYRSNDFTVAITLANPKFTKKQGTVVIHFLQSITSRIALGAEIACLRGSKVPGGQQTVMCMAF

RHSTGLTTLSGTIGEAGLHLCYHRKASSQLEIGVELETNTRTHQSIATIVYQVNVPYADLLFRGIVNSET

TVGGVFEKKLYPIPESSLIISALLNHKKQQFRVGVGLNIGQ

>Dgrim_XP_001991309

MGNVLAASSGVEATTARSNSVAASLGMPEPSANSTEATGSSSSMSSELSDAAKDNTLDNPGTVEELHKKC

KDVQAITFEGAKIMLNKGLSNHFQVSHTINMSSTVPSSYRFGATYVGTKQYSPTEAFPVLLGDIDPSGNL

NANVIHQFSSRLRCKFASQIQDSKMLATQMSTDYRGNDFTASITLGNPSIFTNSGVIVGQYLQSVTQRIA

LGAEVAYQYGPNVPGRQIAIVSALGRYSTGDSIWSGTVGPSGLHLCYYQKASEQLQIGVEVETSLRMQES

VATIAYQIDLPKADLVFKGSVDSNWHISGVLEKRLQPLPFTFALSGRMNHLKNNFKLGCGLIIG

>Mdom_XP_005185138

MGNVMAASSTPSSAAASLDKAAKSGVPAPPTAPAGLAAVDAPAVDVKLENPGTLEELHKKCKDIQAMTFEGAKVMLNKGLSNHFQVSHTLNLCSTQPSGYRFGATYVGTKQYSPSEAFPVLLGDIDPSGNLNANVIHQFSPRIRCKFASQVQESKITVAQLTTDYRGDDFTASLTIGNPNIFNNSGVFVGHYLQSVTKNIALGAELAYQYGPNVPGGQITVLSAVGRYTSGDSIWSGTLGPGGVHICYYQKASEQLQIGVEVETSLRMQESVATLGYQVDLPKADLVFRGSFDTNWNVCGVLEKRLQPLPFSFALSGRLNHTKNQFRLGCGLIIG

>Dwil_XP_002062810.1

MGNVLAASSPTSTTMSGQSGSGVLPPPSSPPMSTLPASLTEAIKAEIKRTPVILENPGTVEELHNKCREI

QASTFEGARIMLNKGLSNHFQISHTLNMTASSSNGYRFGATYVGCKQYGPTEAFPVLLADIDASGNLNAN

IIHQMSSRLRCKFAAQFQDSRLMATQLTNDYRGDNYSASLTLGNPGILSGSGVVVAQYLQSITRRLAMGT

ELAYQYGPNVPGRQIALLSLVGRYGDKDCVWSGTLGLNGLHVCYYRKASDQLQIGVEVETSLRMQESVTT

LAYQVDLPKADLVFRGSFDSNWHVCGVLEKRLQPLPFAFALSGRMNYVKSVFTLGCGLMIG

>Dwil_XP_002071695

MGNVLAASSGTGPGLNSSVAASLGLPEPAPEQPQQSSRSAAVTENSGASAITTSKDAALENPGTVEELHK

KCKDIQAITFEGAKIMLNKGLSNHFQVSHTINMSTTAPSGYRFGATYVGTKQYSPTEAFPVLLGDIDPSG

NLNANVIHQFSPRLRCKFASQIQDSKMVASQLTTDYRGDDFTASITLGNPSIFTNSGVIVGQYLQSVTQR

VALGAELAYQYGPNVPGRQIAIVSAVGRYAADNSTWSGTLGQSGLHLCYYQKASDQLQIGVEVETSLRMQ

ESVATIAYQIDLPKADLVFRGSIDSNWHISGVLEKRLQPLPFSFLLSGRMNHVKNNFRLGCGLMIG

>Pcoq_MNCL01000021

LLTGEIEKCGNMQASIVHFVTNKIRVKFGAEYQGRHLLGGQFIADYLGESFTASMGIANPNVTNGTGIFLGQYLQSVTKNLALGSEIGCQYGMLHPQGTITVFNFISKYKGESSIFSTSIGGSGVHLSYVKEASPQLKVGVEFNTNFDGQSTYTTIGYQVNIPVADLSFRASLDTNWNICSVLEKKLKPFPFSFSLSALLNQKTGKFKMGCGMLIG

>Pcoq_MNCL01000193

DLMPITFEGAKIMVNKGLSNHFQVSHTLNLCSSTPSGYRFGATYVGTKQYSPNEAFPVMLGDIDSSGNLNANMIHQFTPKFRCKFASQIQQSKIIAAQLTTDYKGDDFTASLTIGNPNIINNSGVIVAHYLQSVTDSLAFGGELAYQYGPNVPGGEFAVVSAAARYIHKTSTWSGTIGPGGIHLCYYQKASEQLQIGVEVETSFRMQESVASIGYQVDLPKADLLFRGTI

>Mdes_gi309241287

VTKEPATSTQQQQTESIFDISDRENPGTIEELHKRCKDVMPTNFEGAKLMIQKGLSSHFQVSHTINLSSTTPSGYKFGATYVGTKQLSPSEAFPVVLGDIDPSGNMNANIIHQFTPNIRCKFGSQIQENQIAAAQLTTDYRGADFTASLTLANTDLINESGVAVAHYLQSVTKNLALGGELAYQYGRAVPGGEIAVISAAARYQNGLATWSGTAGLAGIHACYYRKASDQLQFGVEVETNLRVQEAVGTFGYQIDLPKADLVFRGMLDTNWNVGAVMEKRLLPLPFTLAISGHLNHVKNQHRL

>Cnas_XP_031624560

MGNVLAASQSQPSLPPSPAVTKETLENASTAQTDNFFNVDDRENPGTIEDLHKKCKDVMPTNFEGAKLMI

QKGLSSHFQVSHTINLSSTTPSGYKFGATYVGTKQLSPTEAFPVVLGDIDPSGNVNANIIHQFTPNIRCK

FGSQIQDNKISAAQLTTDYKGSDFTASLTLANTDLINEAGVAVAHYLQSVTKNLALGGELAYQYGRAVPG

GEIAVISAAARYQNGLSTWSGTAGLAGVHVCYYRKASEQLQFGVEVETNFRVQEAVGTFGYQIDLPKADL

VFRGMLDTNWNVGAVLEKRLLPLPFTLAISGHLNHVKNQHRLGIGMIIG

>Smos_VUAH01006196

ENAIPAQTDSFFNVSDRENPGTIEDLHKKCKDVMPTNFEGAKLMIQKGLSAHFQVSHTINLSSTAPSGYKFGATYVGTKQLSPTEAFPVVLGDIDPSGNMNANIIHQFNQNIRCKFGSQIQDNKISAAQLTTDYKGSDFTASLTLANTDLINESGVAVAHYLQSVSVTKSLALGGELAYQYGRAVPGGEIAVISAAARYQNGLSTWSGTAGLAGVHVCYYRKASEQLQFGVEVETNFRVQEAVGTFGYQIDLPKADLVFRGMLDTNWNVGAVLEKRLLPLPFTLAISGHLNHVKNQHRLGIGMIIG
