# Supplementary material for: Genomic signatures of globally enhanced gene duplicate accumulation in the megadiverse higher Diptera fueling intralocus sexual conflict resolution
Source: PeerJ. 2020 Oct 12;8:e10012. doi: 10.7717/peerj.10012 (PMC7560327; doi:10.7717/peerj.10012)
Supplement: Supplemental Information 9 [file peerj-08-10012-s009.zip › Mdh2 CG7998 protein sequences.docx]

>Dmel_Mdh2_CG7998

MLKQVTKQLALQGVRTFSVGQQNNYKVTVCGAAGGIGQPLSLLLKQNPLVTDLALYDIVHTPGVAADLSHIDTKSKTAGFIGADQLGDSLKGSDVVVIPAGVPRKPGMTRDDLFNVNAGIIKDISNSIAKNCPKALVAIITNPVNTCVPIAAEILKKAGVYDPKRLFGVSTLDVVRARAFIGHALGVDPQTVQIPVIGGHSGVTILPVLSQSQPLFKGNQDTIEKLTVRIQEAGTEVVKAKAGAGSATLSMAYAGARFAGSLLKGLNGEKNVIECSYVQSTVTEATFFSTPLVLGKNGVQENLGLPKLNDYEKKLLEAAIPELKKNIQKGIDFANA

>Dmel_CG10748

MLLLTSLKSLAKPATWGVVVRTLKVAVVGAGGGIGQPLSLLLRRCPGIDELALHDLSEMKGIATDLSHISQTGKVIGFTGEKELESAVSGADVVVVAAGMPRLPGMQRDHLMAANGNVAVKVATAISNASPRAHLAFITNPVNMIVPAAAEVLMAHGTFDSRRLFGITTLDVVRSKKFIGDSMNISPDDVNIPVIGGHAGITILPLISQCQPIYRCDLQEIQNLTHRIQEAGTEVVNAKAGKGSATLSMAYAGATFVNSLLRGIAGQDGLIECAFVASKLTDAPFFASPLELGKDGIKRYIPLPQMSDYEKEALEKLLPILRQNADEGVNFAKMILSGQSHSPIPAALP

>Dmel_CG10749

MFLASRLLSHVGNLPPKVQQLGYINRGLKVAVVGSVGGIGQPLSLLLKHNPQISTLSLYDIKNTTGVGVDLSHINTRASVCPFEGKNGLKKAMDKADIVVIPAGLPRKPGMKREDLVDVNASVACEVAFAASEVCPGAMLAFITNPINVIVPIVATILKAKGTYDPNRLFGVTTLDVVRAQTFVADILNVDPQKVNIPVIGGHTGRTILPILSQCDPPFKGTDKEREALIQRIQNAGTEVVNAKDGLGSATLSMAFAATQFVSSLIKGIKGSKDECIVECAYVESDVTEAQFFATPLILGPQGVKENTGLPDLDDEERKALNGMLPILKESIAKGIKLGEGMICSCA

>Dvir_XP_002054903

MLKQVTKQLALQGVRNFSVSQQNNYKVTVCGASGGIGQPLSLLLKQNPLVTDLSLYDIVHTPGVAADLSHIDTKSKTVGFMGADQLGASLKGSDVVVIPAGVPRKPGMTRDDLFNVNAGIIKDISNAIAKNCPKALVAIITNPVNTCVPIAAEILKKAGVYDPKRLFGVSTLDVVRARAFIGHALGVDPQSVQIPVIGGHSGVTILPVLSQSQPQFKGNQDAIEKLTVRIQEAGTEVVKAKAGAGSATLSMAYAGARFAGSLLKGLNGDKNVIECSYVQSNITEATFFSTPLVLGKAGLQENLGLPKLNDYEKKLLEAAIPELKKNIQKGIDFANA

>Dvir_XP_002055293

MFGGQIRSSLLASRHICWAGLSPLACRLQVRRNFKVSVVGAAGGIGQPLSLLLMYNSMITELVLHDLVNV

KGVSADLSHVSTATDVKGFQGPEQLEKAVRGADLVIITAGMGRGPGMTREQLFEINAKIIIQTVNAIAKN

SAHALIAIVTNPINTLVPMAAEVLKRNQVFDPNRLFGVTTLDCVRAERFIGNYFNIDPKEVKVPVIGGHS

GITIMPILSQCKPAVNADEECIAALVQRIQMAGDEIVLAKEGKGSATLSIAYATNRFADALLKGLKGDKT

PIESAYVQSDLTEACFFATPLSFGPKGIEENHGIPELNDVEKLALESAVSDLKKSIEKGISYV

>Dvir_XP_002050073

MLGCACTCLKVMLKAQNSSAVVQRKGNWSWNGLGVRNYKVTVVGAGGGIGQPLSMLLKQNPLIDELTLHD

VGDIKGVAADLSHICTSTQVDFFDGVKQQELIDSLHDSHVVVVPAGLPRQPGMTRDQLEDANSGVAMAVS

CAVGMACPEALLAFITNPINTIVPIAAEFLKAKGVFDPNRLFGVTSLDVVRAKTFIADYMNIDPATVEIP

VIGGHAGKTILPIFSQCSPKFTGEDEDVKRLTERIQEAGTEVLNAKAGKGSATLSMAYAAAYFVNALLRG

LNDEPGVIECAYVASDATELAFLATPLELGPNGIKKNLGLPSLNADEEAALQKLLPELRQNIERGISYAA

KIIDAAKPDQEPIKEPDCKVEDSAKYAQSN

>Mdom_XP_005190203

MLKNFQRCISQETFQFCKICIVGANGGVGRSLAQQLKQNNRFSEICLYDITCTKGLRADLSHINTLPEVT

AYSGAENLIESLRCCDIMVLTGGQSHSKGITSREQMFEANAKIMVKLIEALVKANKEQPHIHIVTNPINS

LVPLTAELLKKHGIYNARRLSGCTKVDGMRAATFLAEHMGVDPRYVEVPVIGGHSSKSIVPLISQSQPKF

ALDDETQMKIALRIIHGGEEVVLAKNHKGAAQLSMGYCVAQFCDSLVRSMQGEKNVKEVGFIPSDVNDVP

YFACQFELDKEGIRGCKRLPPMLPFEINLYNQAREDIRQNVEIAMEFMEKNKLK

>Mdom_XP_005187487

MLKQATKQLAIQSMRSFSTSRQNNYKVTVCGASGGIGQPLSLLLKQNPLVTSLSLYDIVHTPGVAADLSH

IDTKSSTHGYIGADQLGDALKGADVVVIPAGVPRKPGMTRDDLFNVNAGIIKDIAATVAKQCPKAMVAII

TNPVNTCVPIAAEVLKKAGVYDPKRLFGVSTLDVVRARAFIGEALGVDPQQVNIPVIGGHSGVTIMPILS

QCQPTFKGSADTIDKLTKRIQEAGTEVVKAKAGAGSATLSMAYAGARFAGSLLKGLDGQKNVVECSYVQS

TVTEAAFFATPLVLGKNGIQENLGLPKLNDYEKKLLEAAIPELKKNIQKGVDFANA

>Ccap_XP_004536932

MLKQVTKQLATQSVRGFSTTKQNNFKVTVCGASGGIGQPLSLLLKNNPLVSNLALYDIVHTPGVAADLSH

IDTQSTTEGHMGPEQLGKALKNADVVVIPAGVPRKPGMTRDDLFNVNAGIIRDIANEISKSCPKALVAII

TNPVNTCVPIAAEILKKAGVYDPKRLFGVSTLDVVRARAFIGAALGVNPQQVDIPVIGGHSGVTILPIIS

QCQPAFKGDQATIEKLTVRIQEAGTEVVKAKAGAGSATLSMAYAGARFAGSLLKGLNGENGVVECSYVQS

DITEASFFSTPLILGKNGLQENLGLPKLNDFEKKLLAAALPELKKNIQKGIEFANA

>Ccap_XP_004533385

MLRNTGFIFKTCASFSRKLSTTQANKNRVCVIGASGAIGQALSLLLKRDTRVTALSLYDIKKAVGIALDM

SDIDTNSLIGGHEELEMLPKALYCADVVVIVAGQPRKKGMTDDELLKKNADVIIQTMPHIAETCPKALIA

IVTSPVDSLVPLAAEVLKTKNAYDPNRLFGVTTHNAVRARTYISDVLQVDASSVKVPVIGGNSKCTILPI

LTHTKPILKIEDEDDALPIIKKVRDAEETVAKTKGAPPTLVMAHSVAKFTHSLILGLAGLGDPVECAYVE

STVTDCAFFATPLKLGKNGIEKNLGIPSPLAKFEAEMLQELMPELKESIKKGLEYAKKVDKGENKREKSE

KSEKSEKK

>Ccap_XP_004531266

MKSLRARHISRLLRCFSTTTRYNYKVTVVGGCGGIGQPLSLLLKRNTKVKTLAIYDVARTPGIYTDLSHI

DTSVVVEGYQCAENLPAALEKADIVLLVGGAARKPDMTRDDLFKLNVNVVEEVVTTIADKCPKALVGIIT

NPVNSCVPVAAEILKEKNVFDPKRLFGVTTLDLVRARTFIGEILDMDPGKVSIPVIGGHSGETILPILSQ

CKPPLKLDKERATKLIKRIQDAGTEVVKAKCLDGGTSATLSMAHAAYLFTDALLRGLQGDCKPVKCAYVA

SDVSDCAFFSTPLQLGKNGVEKNLGLPKMNKDEEEMYCKAVEKLKKHIKSGVDYFKNKSKCDKKVKEEKKDKDKKC

>Dant_Unigene996

MLKQVSKQLAIQSMRSFSTTGQNNYKVTVCGASGGIGQPLSLLLKQNTLVTDLALYDIVHTPGVAADLSHIDTKSKTTGHMGADQMGQALKNADVVVIPAGVPRKPGMTRDDLFNVNAGIIKDIANSIAKNCPKAMVAIITNPVNTCVPIAAEILKKAGVYDPKRLFGVSTLDVVRARAFIGEALGVDPQQVNIPVIGGHSGVTILPVLSQCQPAFKGNQATIEKLTVRIQEAGTEVVKAKAGAGSATLSMAYAGARFAGSLLKGLNGDSNVVECSYVQSNVTEATFFATPLVLGKQGLQENLGLPKLNDEKKLLEAAIPELKKNIQKGIDFANA

>Tdal_comp152343

MLKQLSRPVISLRNFSTSRSNKYKVTVCGASGGIGQPLSLLMKQNKLVTTLSLYDIVHTPGVATDLSHIDTNSTVCGFMGPDQMPDALDGADVVVIPAGVPRKPGMTRDDLFKTNASIVRDIAKCISTKCPKALVAIITNPVNTVVPIAAEVLKKAGVYDPKRLFGVSTLDIVRSRTFIGNSLNVDPQKVTIPVIGGHSGVTIIPVLSQSQPAYKGSQSDIEKLTVRIQDAGTEVVKAKAGAGSATLSMAYSGARFAGSLLRGLNGESGVVECSYVESCVTDATFFSTPICLGKNGQEENLGLPKLNDFEKKLLEKALPELKKNIQKGVDFAN

>Tcas_XP_973533

MFSRVVRPSLASARSFSTSKQNNVKVAVAGASGGIGQPLSLLLKQSPLVTELSLYDIVHTPGVAADLSHI

ETPAKVKGFNGPENLKKAFEGAEVIIIPAGVPRKPGMTRDDLFNTNASIVQTLAEAAAESAPKALIGIIS

NPVNSTVPIAAEVLKKAGKYDPKRLFGVSTLDVVRANTFVAELKGLNPLEVKVPVIGGHSGVTIIPLISQ

ATPSVTFPPDQLKALTERIQEAGTEVVKAKAGAGSATLSMAYAGARFAISLIRALKGEQNIIECAYVESN

LTEAKYFSTPLLLGKNGLEKNLGLGKLSDFEQDLLKKAIPELKKNIQKGEDFVNKK

>Amel_XP_392478

MLPQYLKPILNVTQQGAKRLSTSAKCNAKVAILGASGGIGQPLSLLMKQSPLVTELSLYDVVNTPGVAAD

LSHMDTPAKVKAYTGPEELKDALKGTQVVIIPAGVPRKPGMTRDDLFSTNASIVRDLTQAIAEASPKAFI

AIISNPVNSTVPIASEVLKKAGVYDPNRVFGVTTLDIVRANTFIAEAKGLNPQNVSVPVIGGHSGVTIIP

LISQTKPSVSFPEDKVKALTMRIQEAGTEVVKAKAGTGSATLSMAYAGARFGFSLIKALNGERITEYCYV

KSDVCDTKYFSTAVVLGKAGIEKNLGIGNLNAYEKELLNAAIPELKKNVEKGEKFMNK

>Aaeg_EAT40089

MNISVRQMTERCCFTPGSPAGQAGHGARKQGKRKGGGASIHSVLPCHYCSVHPLACAVWCRFFSKCDQLFCEKGHFPQEPCKMFARTLKTVATQGVKNFSTTSQNNVKVAVCGASGGIGQPLSLLLKQSPLVTELSLYDIVHTPGVAADLSHIETHSKVTGYNGAENLEKALANADIVIIPAGVPRKPGMTRDDLFNTNASIVRDLAAGCAKACPKALIGIISNPVNSTVPIACETLAKAGVLDVKRVFGVSTLDIVRANTFIGEAAGVDPQKVNVPVIGGHSGVTIIPVLSQATPSVNFPQDKIAALTERIQEAGTEVVKAKAGAGSATLSMAYAGARFALALARAMKGEQNVIECAYVRSDVTEAKYFSTPLLLGKNGLEKNLGLPKLNAFEQELLKKALPELKKNIQKGEDFVGKK

>Agam_EAA01572

MFARAVKTAACQGAKNFSTTSQNNVKVAVCGASGGIGQPLSLLLKNSPLVTELSLYDIVHTPGVAADLSHIETQSKVTGYNGPENLEKALKGADIVIIPAGVPRKPGMTRDDLFNTNASIVRDLAAGCAKACPKALIGII

SNPVNSTVPIACDTLQKAGVLDPRRVFGVSTLDIVRANTFVGEAAGVDPQKMSVPVIGGHSGVTIIPVLS

QTKPGVNFPQDKITALTERIQEAGTEVVKAKAGAGSATLSMAYAGARFALALARAMNGEQNVIECAYVRSDVTESKYFATPLLLGKNGLEKNLGLPKLNAYEQELLKKAIPELKKNIQKGEEFVKKN

>Pcoq_MNCL01000101

IMRHLCKIFTFVHTKRFSTSALKQHKVTIIGAAGGIGQPLSLLLKCCPRIKTLMLYDIVHALGVAADVSHIDTDAAVHGFVEVNSLPEALKNSDIVIIPAGVPRKPGMARDDLFNVNANVIKETAGAISKNCPKALVAIITNPVNSVVPIAGEVMRKSGTFDPQRLFGVTTLDLVRARKFIGDELNVNPQCVNIPVIGGHSGVTIIPLLSQSTPEYNKSGDEVVKLTKKIQNAGTEVVEAKMGAGSATLSMAWAGALFVGSLLKGLDGEKDIVECSYVHSKIVPGVEYFATPMVLGKDGVEKNMGLPEMNEYEKCLLDQALPELKDNIEKGKKFA

>Pcoq_MNCL01000001

QNNFKVAVCGASGGIGQPLSLLLKQSSLVKQLALYDIVHTPGVAADLSHIDTQSDVKGYMGPEQLDQCLKGXGADVVVIPAGVPRKPGMTRDDLFNVNAGIIKTLAASISKNCPKACVAIITNPVNTCVPIAAEIMKKVGFSEXGTYDPKRLFGVSTLDIVRARAFIGHALKVDPQKVEIPVIGGHSGVTIIPVLSQSQPKYKGSQDDIQKLTVRIQEAGTEVVKAKAGAGSATLSMAYAGARFAVSLLRGLSGEKGVVECSYVQSNVTEAAFFSTPLVLGKNGIEQNLGLPQLNDFEKKLLDTAIPELKKNIKKGVDFASS

>Pcoq_MNCL01000058

KVSICGAAGSVGRIVSFFLKQSPYDVEISLHDNSHTVSMLSKQLQLINTRNCVETFLGPMKMRFALKXFKDADIVVIITKAKGKIGYGDFFDQNIPIVSNIARSCIEVCPEAILAIATNPINSIVPSINEIYKSYGLSHSDKIMGITAIDSMLASVSFAQIFSLDPSDVFVPVTGGNTGDTIVPLFSQSSPSPEQELSKEDTERLLTQFFTYKTQCVNSELTAAFAITRFIQSIIRALLGEIDVVECAYVAAPEGAPVSYLSTLIDLGERGIRNIYGPPSNINRTEQKLLNIALSKLEKDISIGEKYAS

>Mdes_AHB50501

MFPRLTVSAVKQAGKQFSTTSQNNTKVAVCGASGGIGQPLSLLLKQSPLVSHLSLYDIVHTPGVAADLSHIDTPAKVEGFNGAENLEKALKGADVVIIPAGVPRKPGMTRDDLFNTNAGVVRDLAVAISKACPKALVGIITNPVNSCVPIASEVLKKAGTYDANRIFGVSTLDVVRACAFIGEASKTNPMDVKIPVIGGHSGVTIIPLLSQCSPAVNFPEAQVKALTQRIQEAGTEVVKAKAGAGSATLSMAYAGARFALSLVRALNGDPNVVECSYVASNVTAAKYFATPLKIGKNGIEKNLGLPQMNAYEKSLLESAIPELQKNIKKGEDFVQNN

>Cnas_XP_031639088

MFSRLSVSIAKQAGKNFSTTSQNNVKVAVAGASGGIGQPLSLLLKQSPLISELSLYDIVHTPGVAADLSH

IDTPAKVLGFNGAENLETALKNADVVIIPAGVPRKPGMTRDDLFNTNAGIVRDLAVAISKVCPNALVGII

TNPVNSCVPIASEVLKKAGTYDPNRIFGVSTLDVVRAQAFIGEATNTNPLDVKIPVIGGHSGVTILPVLS

QSVPSVNFSQDKIKALTERIQEAGTEVVKAKAGAGSATLSMAYAGARFALSLVKALKGDPNVVECSYVRS

DVTAATYFATPLKLGKNGVEKNLGLPKLNAFEQTLLEAAIPELQKNIKKGEDFVKNN

>Smos_VUAH01000074

MFSRLSVSVAKQAGKNFSTTSQXNNVKVAVAGASGGIGQPLSLLLKQSPLVSHLSLYDIVHTPGVAADLSHIDTPAKVTGYNGKENLEKALENADVVIIPAGVPRKPGKXGMTRDDLFNTNAGIVRDLAVAISKVCPKALVGIITNPVNSCVPIASEVLKQAGTYDPNRIFGVSTLDVVRAQAFIGEKTNTNPLDVKIPVIGGHSGVTILPVLSQSQPSVKMSQDQIKALTERIQEAGTEVVKAKAGAGSATLSMAYAGARFALALVRALKGDPNVVECSYVRSDITAATYFATPLKLGKNGIEKNLGLPKLNAYEQSLLDAAIPELQKNIKKGEDFVKSN

>Smos_VUAH01000016

QQKPIKVTVIGATGGVGQPMSLLLKENTLISELALWGDARGGIGKIPGIAADLSHISTPVKVLGYSGPGNISAALQNADVVVIPAGVARKKGXGMSRQELFKANAEILRDIAVKISTVCPKALIAIITNPVNSGVPVVSEVLKKQGTYDPNRIFGVTTLDIVRAQTFIGDEMNVSPLEVNVPVIGGHSDESIIPVLSQTHLNSVPKKALTLPKFEELTKRIQVAGSEIVTAKNGDGSATLSTAYAATRFVNALVEALTGDPNVFEYAYVRSNVTTAKYFSTRLKLGKNGIEKIFGIPELNEFEKKLLHEAIPLLQKDIEAGEKYVK

>Cqui_XP_001863970

MVTIACDTLPKAGFLEPKCAFGISMLYIVRPNTFIGEGVDFGGSTFPVNIPVIGGHSGQTIIPDKIATLAVRIQEVLKTKTGAGADTLTTAYAEARFALAMAHAMNGKKNNVLEKNLGLPKVNAYGQELLKKDISELKKNIRKGEEFVKNYVTEKEVH

>Cqui_XP_001841671

MIGIISNPVNSVVPIACDTLLKAGVLDTRCMFGISTVEIIRANTFISEVIGRQSGETIIPVHPRASAALTEHIQELLLLSKVNDYEQELLKKVIPKLIKNIQKGEEFDMNKKKNLDLAENGDDFELEEVGFSPAISNGGGPRCVKQGCGVGVGVGVGVGGVGSFWGPGVGVGVVKTRTAGVGVGAGVG

>Cqui_AAWU01013293

RRMIGIISNPVNSVVPIACDTLPKAGVLGTRRVLGISTLEIIRANTFIGEGVDIDGSTFQSLEVTPARPSSQCIPELPKNKIAALKERIQEVLIAKAGAGAXATYFSXPLLLGKNSLEKNLDLSKVNVYEQEMLNKAIPKLMKNIQKGEEFVMNNGCRHRRVNIPVIGGHSGETIISVHPRAS

>Cqui_XP_001849862

MFARTLKSVAAQGAKNFSTSGQNNVKVAVCGASGGIGQPLSLLLKNSPLVTELSLYDIVHTPGVAADLSHIETRSKVTGYNGPENLEKALAGADIVIIPAGVPRKPGMTRDDLFNTNASIVRDLAAGCAKACPKALIGIISNPVNSTVPIACDTLAKAGVLDPKRVFGVSTLDIVRANAFIGEASGVDPQKVNIPVIGGHSGVTIIPVLSQATPSVSFPQDKIAALTERIQEAGTEVVKAKAGAGSATLSMAYAGARFALALARAMNGEKNVIECAYVRSDVTEATYFSTPLLLGKNGLEKNLGLPKLNAYEQELLKKAIPELKKNIQKGEEFVKKN
