# Supplementary material for: Genomic signatures of globally enhanced gene duplicate accumulation in the megadiverse higher Diptera fueling intralocus sexual conflict resolution
Source: PeerJ. 2020 Oct 12;8:e10012. doi: 10.7717/peerj.10012 (PMC7560327; doi:10.7717/peerj.10012)
Supplement: Supplemental Information 9 [file peerj-08-10012-s009.zip › GPDH protein sequences 2020.docx]

>Dmel_GPDH-1B

MADKVNVCIVGSGNWGSAIAKIVGANAAALPEFEERVTMFVYEELIDGKKLTEIINETHE

NVKYLKGHKLPPNVVAVPDLVEAAKNADILIFVVPHQFIPNFCKQLLGKIKPNAIAISLI

KGFDKAEGGGIDLISHIITRHLKIPCAVLMGANLANEVAEGNFCETTIGCTDKKYGKVLR

DLFQANHFRVVVVDDADAVEVCGALKNIVACGAGFVDGLKLGDNTKAAVIRLGLMEMIRF

VDVFYPGSKLSTFFESCGVADLITTCYGGRNRRVSEAFVTSGKTIEELEKEMLNGQKLQG

PPTAEEVNYMLKNKGLEDKFPLFTAIHKICTNQLKPNDLIDCIRNHPEHMQNL

>Dvir_XP_002051368

MAEKVNVCIVGSGNWGSAIAKIVGANAAALPEFEERVTMFVYEEMIDGKKLTEIINETHENVKYLKGHKL

PTNVVAVPDLVEAAKNADILIFVVPHQFIPNFCKQLLGKIKPNAIAISLIKGFDKAEGGGIDLISHIITR

HLKIPCAVLMGANLANEVAEGNFCETTIGCTDKKYGKVLRDLFQANHFRVVVVEDADAVEVCGALKNIVA

CGAGFVDGLKLGDNTKAAVIRLGLMEMIRFVDVFYPGSKLSTFFESCGVADLITTCYGGRNRRVSEAFVT

SGKTIEDLEKEMLNGQKLQGPPTAEEVNYMLKNKGLEDKFPLFTAIHKICTNQLKPKDLIDCIRNHPEHM

QTL

>Dmel_GPDH-2

MDKIMICIIGSGNWATTIARNVGRNVLNSQTLDEKVPMYVYEEIVEGRKLTEIINTTHIN

SKYMPNFELPPNIVAVDDIVTTARDADIIIFAIPPTFVSSCCKTLLGKVKPTAHAVSLIK

GFERGDDGQFVLISQIIMRQLKIPCSVLVGCNLAHELAHDHFAEGTVGCRDQKYYRVLHD

IFKSPTFRVVVTEDADCVEICSTLRNIIAFAAGCSDGMELNENTKGGIIRRGFLEMLQFV

DVFYPGCRMGTFFESCGISDLVTSCYANRNRKLAEAFVKTGKPLSELEHILIPGHEPLGP

VTAELVHHMLKKKGLEDKFPLFTTVYRICTGDYPLQRLVETLIKAREDIFHPLHTFQL

>Dvir_XP_002049040

MTDRLNVCIIGSGNWATTIARNVGLNVANSQLMEPKVNMYVYEEYIDGRKLTEIINTTHINVKYMPNFVL

PPNVFALDDVVTTARDADILIFAIPPTFVSSCCKTLLGKVKPLAHAVSLIKGFERGDDGQILLISHLIMR

QLKIPCSVLVGCNLAHEISNGNFAEGTIGCRDQKYMRILQDIFKSATFRVVVTDDADCVEICNSLRNVIA

FGAGCADGMELNENTKAGIIRRGFLEILQFVDVFYPGSRLATFFESCGLSDLVTSCYANRNRKLAEAFVK

TGQPLSELEHILVPGHEPLGPITSELIHIMLKKKGLEDKFPLFTTIYRICTGVYPLSRLVETLRFAREDI

YHPNHIFQL

>Dmel_GPDH-3

MAGKLKICIIGAEGWGSAIAAVVSNNVLEGDFDSRVHLYVYDEMIRDTALSEIINTRHEN

VKYLPGIKLPNNLIAVNDLLEAAQNADILVFSTPLEFVQSYCNILSGNVKESAFAVSMTK

GLLSENGEGIELVSHAISESLGIPCYSMMSAHSAMEMAQGKLCEVTIGCSDNSHSKLLIS

AMQTNNCRVISVNDVDGVELCGTLTDVVALGAGFIDGLRLGENARLAAIHLGVKEIMRFI

KTFFPSSKMSTFYESCGVTNAVASSFVDKNVTFAKSLVTSGQTIEEIEANLHSGRKLLGP

MVASNVNAFLENGLMQHEFPLFTAIHLICQSEAPPELMIEALRNHPDLSSSSISHL

>Dvir_002054373

MPKHPKVCIIGAEGWGSAIATSVCKNVVDVGEFDSRVHIYVYDELVRSNYLSEVMNEQHENIKYLPGIRL

PSNLIAINDLIAAARNADILIFATPHSFVKSYCNILAGNIKSTAYAISLTKGLEHRDGEIELYSHAITHL

LGIPCYSMMNANSAMEMAQGKLCEITIGCNNDAHANQLNYLLQTENCMVFTIDDVDGVELCNTLKDLVAL

SAGFIDGLHLGENARIACLHLGLKEMMRFITNCNPTTNVTTFLESCSLANSVASAYGDKNVTFAKNFVTS

QKTTQEIEASLLNGRKLLGPIIAGEIFAYLDNEDLHDIYPLFSIIHRICEKEVPPQAIVDTLRNHPDLSP

KKKLSHGKFTPNTEMEVENAILDDVIPKTIDKKLNDKTKFINYKPRRDRDDGSDKKKRGRNNETKKDRRD

ENRNSLDEDQRTLALEATENREDIAFMEKLESDVEKAILEEQVPAAVESKIGEEEATQNLDLIHNKSDEN

TNFTALDQDHTQLKYLKAEQEETLLLEAESADVKRSNQEPIGMSESLDGDSERETKAIQMEDKAQQFLAS

KIHNEKVPKPSSEWDWLLDNDKFDSALEEFKQNKDDAAKKPKPLDLSDFKKKLQTYRLERSQEPEPGRMQ

ELKIDSDLLWPNREQDSNEPTRIEALVKSEEATKNETPEQLVEPRAEWSKDEDTVKYTFPEPTPLMEAQT

NVEKAKDLASPDTDVPEAEDTQLKKEWPKENVSYNIEIEPSAEEKISSIEEPEELKDPRKRRKQKSQEMY

DIARQQFWQAQDEHENKTKRDDLQVSEITKQLTKIMTKGQPAPPQPKERPVDYQGDDPYIKENKKVARHS

YGNMLEGDSKQRSRVVVKPFHPPLNPRVRIPRPPFDVRDHEYHTVNFRPPPDLLRTVNLPKKRTTVTCQA

GQTGTRSVSMLPRPVLKLPPFMMRSSVLAIELGFVAAILSRYKSGRK

>Dant_CL1818

MAQKTNVCIVGSGNWGSAIAKIVGANCANLPEFEERVTMFVYEEMIDGKKLTEIINTTHE

NVKYLPGHKLPPNVVAVPDLAEAAKDADVLIFVVPHQFIPNFCKTLLGKIKPNAQAISLI

KGFDKAEGGGIDLISHIITRHLKIPCSVLMGANLANEVAEGQFCETTIGCKDPKYAKILR

DLFQANMFRVVVVEDSDAVEICGALKNVVAMGAGFVDGLGLGDNTKAAVIRLGLMEMIRF

VEIQYPGSKLSTFFESCGVADLITTCYGGRNRKVGEAFVKSGKTIQQLETEMLNGQKLQG

PLTAEEVNYMLKNKNLEAKFPLFTAVHKICSGASKPQDLIESIRIHPEHMQNL

>Ccap_XP_004529383

MSEKISVCIVGSGNWGSAIAKIVGNNCATLPQFEKCVTMYVYEEMIEGKKLTEIINTTHE

NVKYLPGHKLPENVVAVPDLIEACKNADILIFVVPHQFIPNFCKTLLGKIKPNAVAISLI

KGFDKAEGGGIDLISHIITRHLKIPCSVLMGANLANEVALGNFCETTIGCRDIKYAKPLR

DLFQAPYFRVVVVEDSDAVEVCGALKNIVATGAGFVDGLGLGDNTKAAVIRLGLMEMVRF

VDVFYPGSKLSTFFESCGVADLITTCYGGRNRRVAEAFVKTGKSIEELEKEMLNGQKLQG

PPTAEEVNYMLKNKGLEDKFPLFTAIHKICTNQLKVQDLIDCIRNHPEHMQNL

>Mdom_ALHF_11471

MAQKTSVCIVGSGNWGSAIAKIVGANCANLPEFEERVTMYVYEEMIDGKKLTEIINTTHE

NVKYLPGHKLPPNVVAVPDLVEAAKDADVLIFVVPHQFIPNFCKQLLGKLKPNAIAISLI

KGFDKAEGGGIDLISHIITRHLKVPCAVLMGANLANEVAEGQFCETTIGCRDPKYGKILR

DLFQANHFRVVVVDDSDSVEICGALKNIVAMGAGFVDGLGLGDNTKAAVIRLGLMEMIRF

VEIMYPGGKLSTFFESCGVADLITTCYGGRNRKVGEAFVKSGKTIQQLETELLNGQKLQG

PLTAEEVNYMLKNKNLEEKFPLFTAIHKICSGVLKPQDLIECIRCHPEHMQKL

>Mdom_XP_005190609

MEKIHVCIIGSGNLGTALGKILAENAAALSDFEERVNMFVYDELFDGIRLSDLINSHHIN

EKYLPQIQLPDNLVACTDLVETAKYADIIVFVMPKQFIEDFCKTLLGKIKPNAMAISVIK

GFVLNEGDDNGGEVASGSGIQLISQTIMKYLKIPCAVLMGVNLASELTLNRYCEATLGCR

DMKHSKLLKDLFSTPNFRVIVIDDADAVEVCGYLKHLIAFASGILDGLQTNENTKSACLR

FALIEMLRFIDVFYPGCKLSTLFESCGIADVLTVCAGSRNRRLGEAFAKSDRSIEQLETS

LMGGEQVLGPLTANAVYLMLQQKGLQERFPLFTIIHRICKREVKPEELLHCIATMPNIIY

HPTQIFPL

>Gmor_GMOY009911

EKVSICMVGSGNWGTAVAKVVAENAIRLEDVENRVNLFVYDEIFEGKRLSETINQHHINSKYLPYVKLPENLVAFTDLVESAKYADIIVFAIPSKHIIDFCKTLLGKVKPNALAVSLIKGFLPSEGG-IELISHTITKYLKVPCAVLVGVNLASEIVANKFCEATLGCRDTKQTRILKEIFKGPSLRIVVVDDADCVEVCGLLKHILAFGTGLLDGLDCNENIRSALIRFGLLEMMHFIDIFFPGSKLGSFFESSGISDLITVCYGSRNRRIAEAYIRTDYTIEQLEGELLKGQKLLGPMVAKEVNHMLNNKGVEAK

>Gmor__GMOY008210

MSEKTNVCIVGSGNWGSAIAKIVGANCAALPEFEDRVTXXXMYVYEEMIDGKKLTEIINTSHENVKYLPGHKLPENVVAVPDLVEAAKNADILIFVVPHQF

IPSQCQMLLGKIKTNALAISLIKGFDKAEGGGIDLISHIITRHLKIPCAVLMGANLANEV

AEGHFCETTIGCREPKYAKTLRDLFQSENFRVVVVQDSDAVEICGALKNVVAMGAGFVDG

LGLGDNTKAAVIRLGLMEMIRFVEIMYPGGKLTTFFESCGVADLITTCYGGRNRRCAEAF

VKTGKTLVEVEAELLNGQKLQGPLTAEEVNHMLKNKKLEEKFPLFTAVHEICTGQIKPQC

LIERIRAHPEHAQASTPAPKL

>Tdal_Td_comp159056

MEKLSVCIVGSGNWGSAIAKIVGANCANMDEFDNRVNMYVYEEVVNGRKLTEIINDTHEN

VKYLPGHKLPENVVAVPDLVEAAKNSDILIFVVPHQFIPSFCKTLLGKIKPNAFAISLIK

GFDKAPGGGIDLISHIINRNLKVPCSVLMGANLANEVAEGNFCETTIGCRDAKHAKIFHQ

LFQSQNFRVVVVDDADAVEICGALKNIVACGAGFVDGLGLGDNTKAAIIRLGLMEMIRFV

DVFYPGSKLSTFFESCGVADLITTCYGGRNRKVSEAFVKTGLTIAQLETELLNGQKLQGP

ATAEEVNYMLKNKGLEDKFPLFTAIHKICTNQLKVEELIECIRSHPEHM

>Aaeg_XP_001653595

MADKVKVCIVGSGNWGSAIAKIVGVNAKRLPNFEDRVTMYVFEEMIDGKKLTEIINTTHE

NVKYLPGHKLPENVVAVPDVVEAAKDADILIFVVPHQFIRGLGAQLLGKIKTSAVGLSLI

KGFDVAEGGGMELISHIITKHLKIPCSVLMGANLAGEVAEEKFCETTIGCRDMKIAQTLR

DLFQTPNFRVVVVDDVDAVEICGALKNIVACGAGFVDGMGLGDNTKAAVIRLGLMEMIKF

VDVFYPGSKLSTFFESCGVADLITTCYGGRNRKVSEAFVKTGKSIKQLEDEMLNGQKLQG

PITAEEVNFMLKNKGMEDKFPLFTAIHRICTAQIKPQGFLDCLRNHPEHM---KQRAKL

>Agam_XP_001687881

MSDKVRVCIVGSGNWGSAIAKIVGANAKRLATFEDRVTMYVYEEMIDGKKLTEIINTTHE

NVKYLPGHKLPENVVAVPDVVEAAKDADILIFVVPHQFIRGLGTQLLGKIKPTAVGLSLI

KGFDVAEGGGMELISHLITKHLKIPCSVLMGANLAGEVAEEKFCETTIGCRDMKIAQTLR

DLFLTPNFRVVVVDDVDAVEICGALKNIVACGAGFVDGMGLGDNTKAAVIRLGLMEMIKF

VDVFYPGSKLSTFFESCGVADLITTCYGGRNRKVSEAFVKTGKTIVELENEMLNGQKLQG

PITAEEVNFMLKSKGMEDKFPLFTAIHKICTGTVKPQGFLDCLRNHPEHM---QKFS--

>Cqui__XP_001841997

MADKVKVCIVGSGNWGSAIAKIVGVNAKRLPTFEDRVTMYVFEEQVDGKKLTEIINSTHE

NVKYLPGHKLPENIVAVPDVVEAAKDADILIFVVPHQFIRGLGQQLLGKIKKTAVGLSLI

KGFDVAEGGGMELISHLITKHLNIPCNVLMGANLANEVAEEKFCETTIGCRDMKVAQTFR

DLFQTPNFRVVVVDDVDAVEICGALKNIVACGAGFVDGMGGGDNTKAAVIRLGLMEMIKF

VDVFYPGSKLSTFFESCGVADLITTCYGGRNRKVSEAFVKTGKSIKQLEDEMLNGQKLQG

PITAEEVNFMLKSKSMEDKFPLFTAIHKICTGQIKPAGFLDCLRNHPEHM---QTFS--

>Llon_LLOTMP002548

ADNIKVCVVGSGNWGSAIAKIVGHNAAKLPNFCDRVTMYVYEEMIDGKKLTEIINEQHENVKYLPGHKLPPNVVAVPDVVEASKDADILIFVIPHQFIRGLAAQMLGKIKTTAVGLSLIKGFDVAEGGGIALISHIVTKHLNIPCAVLMGANLANEVADEKFCETTIGCKDMKIAPVLRDIIQTEYFRVVVVDDEDAVEICGALKNIVACGAGFVDGLKLGDNTKAAIIRLGLMEMIKFVEVFYPGAKLSTFFESCGVADLITTCYGGRNRKVSEAFVTSGKSIKQLEDEMLNGQKLQGPITAEEVNFMLKNKGMEDKFPLFTAIHRICTGALKPKDVIDAIRTHPEHMQTF

>Ppap_JP550838

ADNIKVCVVGSGNWGSAIAKIVGHNAAKLPNFCDRVTMYVYEEMIDGKKLTEIINEQHENVKYLPGHKLPPNVVAVPDVVEASKDADILIFVIPHQFIRGLAGQMLGKIKPTAVGLSLIKGFDVAEGGGIALISHIVTRHLNIPCAVLMGANLANEVAEEKFCETTIGCKDMKIAPVLRDIIQTEYFRVVVVDDEDAVEICGALKNIVACGAGFVDGLKLGDNTKAAIIRLGLMEMIKFVEVFYPGAKLSTFFESCGGADLITTCYGGRNRKVFEAFVTSGKSIKQLEGEMLNGQKLQGPLTGGEVNFMLKNKSMEGKFPLFSALHRICTGVLKPKDIIDGIRTHPEHMQTF

>Tcas_XP_008201416

MIGFLSFYQSLIGRIRIDVGRRFWAVSTLSARFKPQQSRQSLTSKRSSCHSTNLFESSARMSQKLKKVCI

VGSGNWGSAIAKIVGSNAKKLPHFDDKVTMYVYEEMINGKKLTEIINETHENVKYLPGHKLPPNVVAVPD

VVEAAKEADILIFVVPHQFIRTLCSTLLGKIKPTAIALSLIKGFDRAEGGGIDLISHIITRHLRIPCSVL

MGANLAGEVADENFCETTIGCRDTKQGPLLRDIIQTDYFRVVVVDDEDTVEICGALKNIVACGAGFVDGL

GLGDNTKAAVIRLGLMEMIKFVDVFYPGGKLSTFFESCGVADLITTCYGGRNRKVSEAFVKTGKSIKVLE

DEMLNGQKLQGPFTAEEVNYMLKNKGMEEKFPLFTAIHKICTGQKPVAEFIDCIRHHPEHMDNSYDRPKC

KL

>Amel_NP_001014994

MAEKLRICIVGSGNWGSTIAKIIGINAANFSNFEDRVTMYVYEEIINGKKLTEIINETHENVKYLPGHKL

PPNIIAIPDVVEAAKDADILTFVVPHQFIKRICSALFGKIKPTAIGLSLIKGFDKKQGGGIELISHIISK

QLHIPVSVLMGANLASEVANEMFCETTIGCKDKNMAPILKDLMETSYFKVVVVEDVDSVECCGALKNIVA

CGAGFIDGLGLGDNTKAAVMRLGLMEIIKFVNIFFPGGKKTTFFESCGVADLIATCYGGRNRKICEAFVK

TGKKISELEKEMLNGQKLQGPFTAEEVNYMLKAKNMENRFPLFTTVHRICIGETMPMELIENLRNHPEYI

DETRNYQECKCSI

>Amel_XP_006558427

MATDAKKRVCIVGSGNWGSAIAKIIGANVVKFNNKFETRVTMYVYEEIVNSQKLSDIINQLHENVKYLPG

HRLPENIVAVPDVIEAAKDADILIFVIPHQFITTLCATLLDKIKPTAVGVSLIKGFDRGDGVNIELISKV

IEKNLRIQCYVLMGANLANEVAEEKFCETTIGCRDKRLAPLLKDLIQTSNFRVSIVEDCEAVEVCGALKN

IVACAAGFVDGIGLGDNTKAAVIRLGLIEMVRFVDTFYTGSKLATFFESCGVADLITTCYGGRNRRVCEQ

YVKTGKTIRQLEEELLAGQKLQGPATADEVHGMLKARNLTDKFPLFTTIHRICTEQIRPADLINEIRAHP

ELPTIGDTQDA

>Pcoq_MNCL01000020

NRGSAIAKIVGANTTRLSQFEDRVNMYVYEEMIEGRRLTEIINETHENVKYLPGHKLPPNIVSVXVAVPDLVEAASGADILIFVVPHQFIRNFCATLLGKIKPTAVGISLIKGFDKATGGGIDLISHIITRHLKVXIPCSVLMGANIATEVADGKFCETTIGCRDLSLSSVFRDLFQAEHFRVVVVDDVDAVEVCGALKNIVACGAGFVDGLGFGDNTKAAVIRLGLMEMIRFVDLFYPGSKLATFFESCGVADLITTCYGXGGRNRKVSEAFVKTGKTIKELEEEMLNGQKLQGPYTAEEVNYMLKNKQMEDKRFPLFTAIHQICKGALPPSEMFNCIRNHPEHM

>Cnas_XP_031633500

MASDKVNVCIVGSGNWGSAIARIVGANAANSAQFNERVTMYVFEEMIEGKKLTEIINETHENVKYLPGKK

LPSNVVAVPDLVEAAKDADILIFVVPHQFIPGQCKQLLGKIKKTAVGLSLIKGFDVAEGGGIKLISSIIT

ENLNIPCYVLMGANLANEVADENFCETTIGCQDKKWAPILRDMMQASYFRVVVVDDVNTVEVCGALKNIV

ACGAGFVDGLKLGDNTKAAVIRLGLMEMIKFVEVFYPGSQLATFFESCGVADLITTCYGGRNRKVSEAFV

TSGKTIEQLEAEMLNGQKLQGPPTAKEVNYMLANKKMEEKFPLFTAIHNICIGKLKPDQLIDCIRNHPEH

ICKHITAKL

>Cmos_VUAH01006225

MASNKINVCIVGSGNWXGSAIAKIVGANAARLENFNDRVTMYVFEEDINGKKLTEIINETHENVKYLPGHKLPPNVVXVAVPDLVEAAKDADILIFVVPHQFIPGQCKQLLGKIKKTAVGLSLIKGFDVAPGGGIELISHIITRHLEIPCYVLMGANLANEVANENFCETTIGCNDKKWAPILRDTMQASYFRVVVVDDINTVEVCGALKXNIVACGAGFVDGLKLGDNTKAAVIRLGLMEMIKFVEVFYPGSKLATFFESCGVADLITTCYGGRNRKVSEAYVTSGKTIEELEQEMLNGQKLQGPPTAKEVNFMLSNKKMEEKITTLHSTLSVSLKSNVFFSCXRFPLFTAIHNICIGKLKPNQLIDCIREHPEHM

>Mdes_AEGA01000508

MASDKVNVCIVGSGNWXGSAIAKIVGANAARLANFNDRVTMYVFEEMINGKKLTEIINETHENVKYLPGHKLPENVVSVXVAVPDLVEAAKDADILIFVVPHQFVPGQCKQLLGKIKKTAVALSLIKGFDIAEGGGIELISHIITRHLQIPCYVLMGANLANEVAEEKFCETTIGCNDKKVAPILRDMMQAHYFRVVVVDDVDAVEVCGALKIKNIVACGAGFVDGLKLGDNTKAAVIRLGLMEMIKFVDVFYPGSKLSTFFESCGVADLITTCYGGRNRKVSEAFVTSGKTIEELEKEMLNGQKLQGPPTAKEVNFMLKAKKMEDKYVFLHIKSNCRTLWFGQVLIHFIKYSRFPLFTAIHDICIGSKKPNQLIDCIRNHPEHM
