# Supplementary material for: Genomic signatures of globally enhanced gene duplicate accumulation in the megadiverse higher Diptera fueling intralocus sexual conflict resolution
Source: PeerJ. 2020 Oct 12;8:e10012. doi: 10.7717/peerj.10012 (PMC7560327; doi:10.7717/peerj.10012)
Supplement: Supplemental Information 9 [file peerj-08-10012-s009.zip › Gpo protein sequences 2020.docx]

>Dmel_Gpo-1

MTSRMFKFGVTAASACVGSVLASYTLDRWNTPHVVNNATVPPKRKRTLPP

RADQIKSLMSGEEFDVLIIGGGATGAGCALDSVTRGLKTALVELDDFASG

TSSRSTKLIHGGVRYLQKAILGLDLEQYRMVKEALAERATMLESAPHLTH

PLPIMLPVYTWWQVPYYWVGIKAYDLVAGDRNVKSSYYLSKKDALELFPM

LKKDKLCGAIVYYDGQQDDARMCLAVALTAARHGATVCNHVEVKELLKKD

DGTGKQVLCGAKVKDHISGKEFTVKAKCIVNAAGPFTDSIRKMDNPTVKS

ICCPSSGVHIVLPGYYSPDQMGLLDPSTSDGRVIFFLPWQRQTIAGTTDL

PCEITHNPTPTEDEIQFILNEIKNYLNADVEVRRGDVLSAWSGIRPLVSD

PNKDDTQSLARNHIVHVSPSNLITIAGGKWTTYRAMAEHTIDAAIKACNL

KPERAEAVTSYLKIEGGQGWTPTMYIRLVQDFGLECEVAQHLAKSYGDRA

FAVSKMASLTGKRWPIIGNRIHPEFPYIDAEIRYGVREYACTAVDMIARR

LRLAFLNVQAAQEALPVIVDIMGEELGWSKDEKERQIKHATEFLANEMGH

SVNRTSKERIPIKLSKEEIQTYIKRFQLIDKDKKGYVSINDIRRALKSFG

DGDVSGEQLHEILREIDTNMNGQVELDEYLQMMSAIKTGDVAYSRFARMA

ELEEQKHEAANLKQISVDRSGGGL

>Dvir_XP_002050587.1

MSSRMFKFGVTAASACVGSVIASWTIDRWNSPHVVNNAMQRPPKRKRTLPMRSEQIKSLMSGEEFDVLII

GGGATGAGCALDSITRGLKTALVELDDFASGTSSRSTKLIHGGVRYLQKAILGLDLEQYRMVKEALAERA

TMLESAPHLTHPLPIMLPVYTWWQVPYFWVGIKAYDLVAGDRNVKSSYLLSKKDALELFPMLKKDKLCGA

IVYYDGQQDDARMCLAVALTAARHGATVCNHVEVKELLKKDDGTGKKVLCGAKVKDHISGKEFTVKAKCI

INATGPFTDSIRKMDDPTVKSICCPSSGVHIVLPGYYSPDQMGLLDPSTSDGRVIFFLPWQRQTIAGTTD

LPCEITHSPTPTEDEIQFILSEIKNYLNADVEVRRGDVLSAWSGIRPLVSDPNKDDTQSLARNHIVHVSP

SNLITIAGGKWTTYRAMAEHTMDAAIKACNLKPERAEAVTSYLKIEGGQGWTPTMYIRLVQDFGLECEVA

QHLAKSYGDRAFAVSKMASLTGKRWPIIGNRIHPEFPYIDAEIRYGVREYACTAVDMIARRLRLAFLNVQ

AASEALPVVVDIMGEELQWSKEEKERQLKHATEFLANEMGHTINRQSKERIPIKLSKEEIQTYVKRFQLI

DKDKKGYVSINDIRRGLKSFGDGDVSGEQLHDILKEIDTNMNGQVELDEYLQMMSAIKTGDVAYSRFARM

AELEEQKHDAANLKQKISVDRSGGGL

>Dvir_XP_002050869.1

MSKFLNVAILAGTSTMLAYSSAPVAQDRNKAPQMKRELPSRSMSIDSLRRTEYDILIIGGGAVGAGCALD

AATRGLKTALVEADDFGSGTSSKSSKLLHGGLRDLDQAISHFDMAAFRRVQTSLNERSNIANLAPHLNQP

VPIMLPVHHWWQLPIYWVKLHLYHLMAPGTTKGFHFINSREALEMFPMMRREIFGAFVFYEGQHDDARMC

LAVILTASRYGADICNHMKVVKLLRDKKNMVVGAIAVDQLTGKLYKIHAKMVINATGPMSDTIARLEDAQ

APAQCTPTWASHIVLPPFYCPEEVGLFDPHTKNGSAIFFLPWLGHTLMGTSDETREESEGPQPTEMEVQY

LLDGIRNYINPNFDVRRCDVLAVWGGYKPLPIQSKALQHSTLKNHVISLGPGKMISIVGGTWTSFRLLAE

KAIDTAIKSGGLEPLRRHSITATHCKLDGADGWSPSMFIRLVQDFGLEEDVAKHLSDTYGSNAFKLAVCA

NTCGGKWPIIGKRLHSEFPYIEAEVRQGVRDYACTLVDMVARRLRVAFLNVHAAEEILPQVANEMARELK

WSKKRKSKEILKARQFLNTQMGHVPKSEQAQLSIPIKMSVHQVQKYAAYFKTLDEHNTGLVSINKCCAAM

KTFGVKEVPVDLMHNVLRDIDCHSQGQVNLYEFLLLMSAIVHGDTAYLRYAKMNLDQKLSAISNRRSKKR

ALPVERSGGGL

>Dmel_Gpo-2_CG2137

MAKIIQMAVMLGSSKLGHGAFGFDERQADSTPVPQMKRELPTRKMCLEAF

KRTHYDILIIGGGAVGSGCALDAATRGLKTfALVEANDFGSGTSSKSSKLL

HGGLRDLEQAISRLDVAAFRRVRCSLQERSHIGKLAPHLSQPVPILLPVH

HWWQAPLYYMKLKIYHLMAPKATRSFQYLSADDALEVFPMLRRQELFGAF

VFYEGQHDDARMCLSVALTAARYGADICNHMKVSRLIKNKDGNVAGAEAV

DQLTGKKYSIRAKVVVNATGHMSDSLRQMEDSEAKPLCTRSWSSIIVMPR

FYCPEEVGVFDPHTPSGRSIFFLPWQGHTLVGSSDEPLNLPEQDTQPTET

DVQSLLDCVKHFVTPNFDVRRCDVLAVWGGYKPLPVQSDTLHHTRFKNHV

IQLGPRNMLSIVGGTWSSFRVLAEKTVNAAIRYGDLSPLRKESVTAESCK

LDGASGWSPKMFIRLVQDFGMERDVAEHLSNTYGSNAFKVAVSADSSGKA

WPIVGKRLHSEFPYIEAEVKQGVRDFACTLEDMVARRLRVAFLNVQVAEE

ILPQVANIMAKELNWSRDQIKKQIRQTRTFLNSQMGQLTKESASQMSIPI

KMSVRQVRKFAGQFRQMDDNNTGYVSIADCCKAMKTMGVKEVPVDLMHDV

LRDIDVHAQGKVNLYEFLLLMSAIVQGNTEYLRYARLYLDHKRQMQGSNF

PTVMQMERAGGGL

>Dmel_Gpo-3_CG7311_AT04695p

MSKLGAILAQSAVHLSEHRQLLSQAFLSSITYQRSRVKVRGNHCCAMKVVPSRAEHVNALKAEEFDVLII

GGGAVGCGCAVDAACRGLKTALIEAEDFASGSSSRTSKLIDGSGSYLGTALREKDVEQLYIMLQMMSERV

TMLKNAPHLNRIQPMIIPIYSFLQMPCTWLGLKVYDWISAASNIRGSHIISREATMYEFPLLKTDGLRGG

VVYYDGQVDDARMCVALVMTAVALGANVGNHMEVVEIMPQEGCCRVVSVKDKISCEQFYIQSKAVINATG

SSTDAIRQMDKEGTAPILLPTLETQVSLPRYFGSGHYGLLSPALKNDDLTVYMVPFENHMVLGSREVDLD

EVSSRGSPTPDPDDVDCLLEAAKKRMDPCVELGRCHVLSAWTAIKASVSCPTDKENDDDKRGTPINSYMI

EVSPDGLITLAGGRWSSYRVMAADAVDLAIKSCGLCDDHVTSSWTQDLKLDGAESWCCMLPLEFVQDYGV

PMDVAHHISDSYGYNGHALFSQAPDLKKRLHPSFPYIEAEIQYAMRNEYACTLVDIIARRLRIAFVDAAA

TLHMLPRILKIMAAENGWDEEEQKEQMVAAQEFLVRQMGLGSIVQPRSTSKSKPKKKGSSEGCCCRAAVR

RGARSYSMEISKKICVQPLVQHRSMSSTFLPLMTSLSYGLGR

>Dvir_XP_002057863

MLAKLRKGLTAVHWKRQPRRLLSAMNTILRRGRNNGKEASDQLPKRKQQVTSLKSENFDVLVIGGGAIGC

GCALEATTRGFKTALIEAEDFASGASCKSSKLIEGSNSYLHAAIQGADLQQIFLLQQVLNERATMLTIAP

HLNRVQPMLIPIYSPLRLPLYWLGLKMYDAMAGMSNVRGSHFLSKEATLNEFPLLRRNGLLGSLVYYDVQ

LDDARMCLALAMTATKHGATVANYVRLHELLPPTSDNGCRTVRVEDTVNDSCFTINSRVVINATGADTDV

VRQLDDSNVSPIAVPKLGTHVALPSYYGSQNCGLLFPSDQAAGQALIMLPFENSMLVGSLDVERPESSEQ

TPTPALEEVECLLAQTRRVLEECVQLSSGHVMSAWTGIKPSIMCPSVENKEEEEANTVPNYLLEVSGNQM

ITLAGGRWSTYRVMALDAINTAIEICQLEPHTDSSITNNLLLDGANDFCCMLSLDLVQAYDLPMDVAQHL

ADSYGTNASHLLANSCRSGRQRLHPKFPYIQAEVTYACQREYACHLVDVIARRLRVAFVNVTAAAEMLPT

VLDIMSEQLFWEKHKKEHELRAAQYFLAEQMGLGSIVKQKKQFKFRESKALEHESTQVNREEYNRHSLNS

NSVQAVLSSVPKQASSSMNSRLSGQMETNATFVGLRGISAPASSEYSPMRVQACIPAPLKSGTIPMNDSA

NQETKTSPSITPSDTVGESVPVSDSSTALSPSTSIQKPNVECAPAESTPTPPIVKKKPKVCSFSPYVIAG

SEPKGPAPSTTIISAGSDSVSGLTESSSSSNQKDTTNSIEPKTNYDSQSMAQCPDLEPQQEQSGNSMLSA

SYVTTDSNDVASTVKQEGTAHIKEDKERPSAECAAIPKNSVVDIVKSELTNSSNKADCGYTTTTNDSATA

LEGISQIAVEAIVLEERPSAESAAIPKKSVVKTELTNWSNKDDCGYTTSTNGSASSLEGSSHSAVEATVL

DKGAAIAKPTSGISRLSVGTDCISPMTGISTAPEGSRPATEKATNPRNSADNFKLAKPAACISIPSVVAA

SENSTSNIVSGTAPEDSSRTKDTTAILESAIASLELLEQTIIKANAGEKSSSCKDTSNTVESSSSLTCPS

ADLHKEDGDKAVVARSNNENDVKKNPTGSDTES

>Dant_Unigene2248

MASTFYKVGVTTLSAAIGSVLATWTLDRVNSTHTVSAEMKAPPKRKRTLVSRAEQMKSLQSGEEFDVLIIGGGATGAGCALDAVTRGLKTALVESDDFASGTSSRSTKLIHGGVRYLQKAILGLDYEQYRMVKEALAERASMLESAPHLTHPLPIMLPVYQWWQVPYYWVGIKAYDFVAGDRNVKSSYLLSKAEALELFPMLKKDKLCGAIVYYDGQQDDARMCLAVALTAARHGATVCNHVEVQELLKKDDGAGNKVLCGAKVKDHMSGKQFTVKAKCIINATGPFTDSIRKMDDPTVKSICCPSSGVHIVLPGYYSPDQMGLLDPSTSDGRVIFFLPWQRQTIAGTTDLPCEITHNPKPTEDEIQFILNEIKNYLNADVEVRRGDVLSAWSGIRPLVSDPNKDDTQSLARNHIVHVSPSKLITIAGGKWTTYRAMAEHTLDAAIKACNLKPERAEAVTSLLKIEGGQGWTPTMYIRLVQDFGLECEVAQHLAKSYGDRAFAVAKMASLTGKRWPIIGNRIHPEFPYIDAEIRYGVREYACNTIDMIARRLRLAFLNVQAAQEALPVIVDIMGEELKWSKDEKEKQIKLATDFLANEMGQMVNRASKEKIPIKLSKEEIQTYIKRFQLIDKDNKGYVSINDIRRALKSFGDADISGEQLHEILREIDTNMNGQVELDEYLQMMSAIKTGDVAYSRFARMAELEEQKHDAANLKTKITVDRSGGGL

>Tdal_Td_comp159562

MTSRLYKIGITTLSAVVGGALATWTFEHRNSAYQVNNAMVRPPKRKRTLPARAEQIKSLQSGEEYDILIIGGGATGAGCALDAVTRGLKTAMVEADDFASGTSSRSTKLIHGGVRYLQKAILGLDFEQYRMVKEALQERAAMLEAAPHLAHPLPIMLPVYQWWQVPYYWVGIKCYDLVAGDRNVKSSYYLSKKDALELFPMLKKDKLCGAIVYYDGQQDDARMCLAVSLTAARHGATVCNHVEVMELLKKDDGTGKKVLCGAKVKDHISGKEFTVKAKCIVNATGPFTDAIRKMDDPDVKIICSPSSGVHIVLPGYYSPDQMGLLDPSTSDGRVIFFLPWQRQTIAGTTDLPCEITHNPKPTEDEIQFILSEIKNYLNADVEVRRGDVLSAWSGIRPLVSDPNKDDTQSLARNHIVHVSKSNLVTIAGGKWTTYRAMAEHTIDAAIKACNLKPERSDAVTSLLKIEGGQGWTPTMYIRLVQDFGLECEVAQHLAKSYGDRAFAVSKMASLTGKRWPIIGNRIHPEFPYIDAEIRYGVREYACNAVDMIARRLRLAFLNVQAASEALPVIVDIMGEELKWSKDEKEKQIKVAQEFLANEMGQMVNRASKERIPIKLSQEEIQTYIKRFQLIDKDKKGYVSINDIRRALKNFGDGDVSGEQLHEILREIDTNMNGQVELDEYLQMMSAIKTGDVAYSRFARMAELEEQKHEAAQLKQKISVDRSGGGL

>Gmor_GMOY010871

MASFVYKLGVAALGTTVGSSLAGWTFDRWNLTRK-VLNPPSRLPIHRSHLISRAMQIQNLQRESEFDVLVIDGGPAGAGCALDAVTSCLKTALVDTDGSDYKQCSIVEEVLRERDSLLESVSHLVHPLPIMLPVYEWWQMPFYWMGTKCYDLAAGYRNVKGSYFLSKQDALELFPMLKKDNLVGAVVYHDGQQDDARMCLAVVVTATRHRAMVCNHVEVLRLLKKDDGKGGYVLYGVEVIDHTTGKKLIIKAKCVVNATGSIRKMDDPTVKSICAPSSCAHIALPGYYSPEQLALLDPSTSDGRVIFFLPWQMHTNVGTIDTPHEVKDKPASCDNGIKYILNEIQDVCVNVDADVRHGDELSARSGIPPSVVYESKEDDTQLWTHSHTIHISPSNLITGTRGKCVATSRAMAERVVDTAVEVCDLKPAWSESIRNTLKTEGGQNWSPTMYRSLVQDYGLECEVAQHLNNLYGERPFEVAKMASLTGKRWPIAGNRIHPDFPCIDAEVRYGAREYACNAVDMMARRLRFSFLNVQATQETLPKICDIMAEELNWYNEEKKKQMKKAKQYLALELGEASQRNRAKHKTYMNLNEEEQEVYTKHLERLDKGEKHSRDFQQRFGDCDISDEQLDDILKDIGANVIGEKNFGDYLKI

>Gmor_GMOY010870

KVGVTALSATIGSVVASWTFDRWNSTHTVKNAVARPPKRKRKLISRDEQIQNLQSGEEFDVLIIGGGATGAGCALDAVTRGLKTALVEGDDFASGTSSKSTKLIHGGVRYLQKAILGLDYEQYRMVKEALAERASMLDSAPHLAHPLPIMLPVYKWWQVPYYWVGIKCYDLVAGDRNVKGSYYLSKRDALELFPMLKKEKLVGAIVYYDGQQDDARMCLAVALTAARHGATVCNHVEVLELLKKDNGKGEQVLCGAKVVDHMSGKKFTVKAKCIINATGPFTDSIRKMDDPTIKSICAPSSGVHIVLPGYYSPEQMGLLDPSTSDGRVIFFLPWQRHTIAGTTDLPCEVTHNPAPSEDEIQFILSEIKHYLNQDVEVRRGDVLSAWSGIRPLVSDPNKDETQALARNHIVHISASNLITIAGGKWTTYRAMAEHTIDAAVKACGLHPERPESVTTLLKIEGGQGWSPTMYIRLVQDFGLECEVAQHLAKSYGDRAFAVAKMASLTGKRWPVIGNRIHPDFPYIDAEIRYGVREYACNAVDMIARRLRLSFLNVQAAQEALPVICDIMAEELKWSKDEKQKQINKAREFLALEMGQAVKRITKDQVNVNLSSEES-----------------------------YGDGDVSGEQLHEILKEIDTNMNGQVELDEYLQMMSAIKTGEVSYSRFARMAELEEQKEEAAQFKRKITVDRSGGGL

>Ccap_XP_004530158

MTSRFYKFGVTALSACIGSLAATWVCTDRNSSPYVVHNEIARPPKRKRTLPPRPEQMKSL

QSGEEYDVLIIGGGATGAGCALDAVTRGLKTALVEADDFASGTSSRSTKLIHGGVRYLQK

AILGLDFEQYRMVKEALQERASMLESAPHLAHPLPIMLPVYQWWQVPYYWVGIKCYDLVA

GDRNVKSSYYLSKKDALELFPMLKKDKLCGAIVYYDGQQDDARMCLAVALTAARHGATVC

NHVEVKELLKK-DENG-KKVLCGAKVKDNITGKEFTVKAKCIINATGPFTDFIRKMDDPN

VKTICCPSSGVHIVLPGYYSPDQMGLLDPSTSDGRVIFFLPWQRQTIAGTTDLPCDITHN

PSPTEDEIQFILNEIKNYLNTDVEVRRGDVLSAWSGIRPLVSDPNKEDTQSLARNHIVHV

SPSNLVTIAGGKWTTYRAMAEHTIDAAIKACNLKPERPEAVTSYLKIEGGQGWTPTMYIR

LVQDFGLECEVAQHLAKSYGDRAFAVSKMASLTGKRWPIIGNRVHPEFPYIDAEIRYGVR

EYACTAVDMIARRLRLAFLNVQAASEALPVIVDLMGEELHWSKDEKERQIKLANEFLAHE

MGQMVNRTSKERIPIKLSKDEIQTYVKRFQLIDKDKKGYVSINDIRRALKSFGDADVSGE

QLHEILREIDTNMNGQVELDEYLQMMSAIKTGDVAYSRFARMAELEEQKHEAAQLKQKIS

VDRSGGGL

>Mdom_XP_005177860

MASRLYQIGVTTLSATIGTVLATWTI-DRWNSPHTAIAEMKRPPKRKRTLVSRAEQMKSL

MSGEEFDVLIIGGGATGAGCALDAVTRGLKTALVEADDFASGTSSRSTKLIHGGVRYLQK

AILGLDFEQYRMVKEALQERATMLESAPHLAHPLPIMLPVYQWWQVPYYWVGIKCYDLVA

GDRNVKSSYYLSKQDALELFPMLKKDKLCGAIVYYDGQQDDARMCLAVALTAARHGATVC

NHVEVLQLHKKNDGTG-KQVLCGAKVKDHMSGKEFNVKAKCIINATGPFTDTIRKMDDPT

VKTICCPSSGVHIVLPGYYSPDQMGLLDPSTSDGRVIFFLPWQRQTIAGTTDLPCEITHN

PKPTEDEIQFILNEIKNYLNADVEVRRGDVLSAWSGIRPLVSDPNKEDTQSLARNHIVHV

SPSNLITIAGGKWTTYRAMAEHTVDAAIKACNLKPERPEAVTSYLKIEGGQGWTPTMYIR

LVQDFGLECEVAQHLAKSYGDRAFAVAKMASLTGKRWPIIGNRIHPDFPYIDAEIRYGVR

EYACNAVDMIARRLRLSFLNVQAAQEALPVICDIMGEELKWSKDEKEKQIKAASDFLAGE

MGLMVNRASKERIPIKLSKDEIQTYIKRFQLIDKENKGYVSINDIRRALKNFGDGDISGE

QLHEILREIDTNMNGQVELDEYLQMMSAIKTGDVAYSRFARLAEMEDLKQEAQALKQKIS

VDRSGGGL

>Agam_XP_562185

MASRLRKFGVTAAGIAIGAALSTYALQHKDTPQYQVQMEEMQRIRRKRTLPSRSEQIKAL

QSDEEYDVLIIGGGATGAGCALDSVTRGLKTALVEADDFASGTSSRSTKLIHGGVRYLQK

AILGLDIEQYRMVKEALHERASMLRSAPHLTRPLPIMLPVYTWWQIPYFWVGIKAYDFVA

GDRNVKSSYYLSRADALELFPMLRGDKLCGAIVYYDGQQDDARMNLAIALTAARHGAAIT

NHVEVLELLKKKGDDG-KDVLCGAKVRDNISKKEWTIKAKCIINATGPFTDSIRKMDNPT

VKEICCPSSGVHIVLPGYYSPQQMGLLDPDTSDGRVIFFLPWLNGTIAGTTDSPCDVTRT

PTPTEDEIQFILSEIKNYLNKDVDVRRGDVLSAWSGIRPLVSDPNKEDTQSLARNHIVHV

SDSKLITIAGGKWTTFRAMAEHTIDAAIKACNLKPERG-CVTDGLWIEGAQGWTPTMYIR

LVQDLGLEVEVAKHLAISYGDRAFAVAKLATLTGKRWPIIGKKLHPEFPYIDAEVRYGIR

EYACTCVDMISRRLRLSFLNVQAAIEALPMIADIMAEELKWSKDEKERQIKQCEHFLQTQ

MGHQANRTLKEKVPINLSKQEVDMYVKRFETIDKEKKGYVSITDIKRAMKSFGDAEVSGE

ELHDILKEIDTNMNGQVELEEYLQMMSAIKSGFVSHSRFAAVAEQEEIRKEQERLKKQIT

IERSGGGL

>Cqui_XP_001845276

MASRLRKLGITAAGVAIGAALSTYALRVSDVSPHHVQMEEMQRIRRKRTLPSRSEQVKTL

QSGEEFDVLIIGGGATGAGCALDAVTRGLKTALVEADDFASGTSSKSTKLIHGGVRYLQN

AIMGLDIEQYRMVKEALHERASMLRSAPHLTRPLPIMLPVYTWWQIPYFWVGIKAYDLVA

GDRNVKTSYYLSREDALELFPMLRGDKLCGAIVYYDGQQDDARMCLAVSLTAARHGAAIT

NHVEVLELLKKKDSEGGKDILCGAKVRDNISKKEWTIKAKCVINATGPFTDSIRKMDNPT

VKTICCPSSGVHIVLPGYYSPQQMGLLDPATSDGRVIFFLPWLNGTIAGTTDAPCEVTRS

PMPSEDEIQFILSEIKNYLNKDVDVRRGDVLSAWSGIRPLVQDPNKGDTKSLARNHIVHV

SESKLITIAGGKWTTFRAMAEHTIDAAIKACKLEPERE-CVTDGLWIEGAQGWTPTMYIR

LVQDLGLEVEVAKHLAISYGDRAFAVAKMAALTGKRWPIIGKKLHPEFPYIDAEVRYGIR

EYACTLVDMVARRLRLSFLNVQAASEALPMVADIMAEELKWSKEEKEKQIAACEHFLQTQ

MGQQVNRQLKEKIPVNLSKSEVDTYTKRFETIDKDKKGYVSITDIKRAMKAFGDAEVSGE

ELHDILREIDTNMNGQVELDEYLQMMSAIKSGNISHSRFAAVAEQEEIRKEQERLRKQIT

VDRSGGGL

>Aaeg_XP_001664283

MASRLRRFGVTAAGVAIGAALSTYALKSSDISPHHVQMEEMQRIRRKRTLPSRSEQVQSL

QSGEEFDVLIIGGGATGAGCALDSVTRGLKTALVEADDFASGTSSKSTKLIHGGVRYLQK

AILGLDIEQYRMVKEALHERASMLRSAPHLTRPLPIMLPVYTWWQIPYFWVGIKAYDFVA

GDRNVKSSYYLSREDALELFPMLRGDKLRGAIVYYDGQQDDARMCLAISLTAARHGASIT

NHVEVLELLKKKN-EAGKEVCCGAKVRDNITKKEWSIKAKCVINATGPFTDSIRKMDNPT

VKSICCPSSGVHIVLPGFYSPPQMGLLDPDTSDGRVIFFLPWLNGTIAGTTDAPCDVTTN

PSPSEDEIQFILSEIKSYLNKDVDVRRGDVLSAWSGIRPLVSDPNKGDTQSLARNHIVHV

SDSNLVTIAGGKWTTYRAMAEHTMDAAIKACNLQPERG-CVTDGLWIEGAQGWTPTMYIR

LVQDLGLEVEVAKHLAISYGDRAFAVAKMAALTGKRWPIIGKKLHPEFPYIDAEVRYGIR

EYACTCIDMVARRLRLSFLNVQAASEALPHIADIMADELKWSKEEKEKQIKACEHFLHTQ

MGQQVNRQLKEKIPVNLSKEEVDLYKKRFDTIDKDKKGYVSIPDIKRAMRTYGDAEVSGE

ELHDILREIDTNMNGQVELEEYLQMMSAIKSGFISHSRFAVVAEQEEIRKEQERLRKQIT

VDRSGGGL

>Llon_LLOTMP008094

KRPLPLRGEQVAKLQSGEEFDVLIIGGGATGAGCALDAVTRGLKTALVEADDFASGTSSRSTKLIHGGVRYLQKAILGLDFEQYKMVKEALHERASMLQSAPHLTHPLPIMLPVYKWWQVPYYWVGIKAYDFVAGDRNVKTSYYLSKKDALELFPMLKGEKLCGAIVYYDGQQDDARMNLAIALTAARHGAAVCNHVQVTELFKKKDASGKDVLCGAKVKDCITNKEWTIKAKCIINATGPFTDHIRKMDDPNVKGICCPSSGVHIVLPGYYSPDQMGLLDPSTSDGRVIFFLPWQRQTIAGTTDLPCDVTHNPKPTEDEIQFILTEIKNYLNKDVEVRRGDVLSAWSGIRPLVSDPNKEDTQSLARNHIVHVSPSNLVTIAGGKWTTYRAMASHTIDAAIKACGLKPESEECQTDGLLIEGAHGWTPTMYIRLVQDFGLDCEVAQHLAKSYGDRAFAVAKLAAITGKRWPIIGKKMHPEFPYIDAEIRYGVREYACTAIDMIARRLRLAFLNVQAAQEALPGIVDIMAEELKWSKEEQQKQLKAASDFLANEMGMLVNRASRDKIPINLTKEEIQMYIKRFQIIDKENKGYVSINDIRRGLKKSGTK-LNSDDMHNILSELDVAYNGRLELSDYLQMMSAIKSGNVAYSRFARMAELEEEHHEKEKLKTKISVERSGGGL

>Ppap_PPATMP000122

MAAKPMKRAKRPLPLRNEQVTKLQSGEEFDVLIIGGGATGAGCALDAVTRGLKTALVEAD

DFASGTSSRSTKLIHGGVRYLQKAILGLDFEQYRMVKEALHERASMLQSAPHLTHPLPIM

LPVYKWWQVPYYWVGIKAYDFVAGDRNVKSSYYLSKKDALELFPMLKGEKLCGAIVYYDG

QQDDARMNLAIALTAARHGAAVCNHVQVTGLIKKKDSSGKEVLCGANVKDCISNKEWTIK

AKCIINATGPFTDHIRKMDDPNVKSICCPSSGVHIVLPGYYSPEQMGLLDPSTSDGRVIF

FLPWQRQTIAGTTDLPCDVTHNPKPTEDEIQFILTEIKNYLNKDVEVRRGDVLSAWSGIR

PLVSDPNKEDTQSLARNHIVHVSPSNLVTIAGGKWTTYRAMACHTMDAAIAGXXXMYIRLVQDFGLDCEVAQHLAKSYGDRAFAVAKLATLTGKRWPIIGKKIHPEFPYIDAEIR YGVREYACTTIDMIARRLRLAFLNVQAAQEALPAIVDIMAEELKWSKDEKEKQLKMATDFLSNEMGMLVNRASRDKIPINLTKEEIQMYIKRFQIIDKENKGYVSINDIRRGLKHFGEADISGEELHEILREIDTNMNGQVELDEYLQMMSAIKSGNVAYSRFARMAELEEEQHEKEKLKKKISVERSGGGL

>Tcas_XP_008194238

MASTLGKIALGGLTTTLGAGLASYLFLTDDKDNRWGAVYAQHSPPKAKRNLPSREEQIKSLQNDSFDVLI

VGGGATGAGCALDAVTRGLKTALVEADDFASGTSSRSTKLIHGGVRYLQKAIMQLDIEQYRMVKEALHER

ASMLQSAPHLAHPLPIMLPVYTWWQVPYYWVGIKMYDVVAGRKTLKSSYLLSKKNALELFPMLRGDKLCG

AIVYYDGQQDDARMNLAIALTATRHGATVANHVSVTGLLKEKDSSGKEIVCGVSVKDEITGNKWNVPAKC

VINATGPFTDSIRKMDNPEVKTICSPSSGVHITLPGYYSPEQMGLLDPSTSDGRVIFFLPWQKQTIAGTT

DLPCDVTHHPKPTEDEILFILEEVKNYLNPDVEVRRGDVLSAWSGIRPLVSDPNKPDTQSLARNHIVHVS

DSKLVTIAGGKWTTYRAMAQETIDAAIAACCLKPVHKECQTDGLLLEGAHGWTPTMYIRLVQDFGLECEV

AQHLSKSYGDRAFTVAKMAALTGKRWPIIGKKIHPEFPYIDAEVRYGVREYAVTAVDMIARRLRLAFLNV

QAAQEALPEIITIMAEELGWSDEEKQKQHKDATIFLQNEMGQNVNRASRDKIPINLSKEEIQLYIKRFQI

IDKDRKGYVSINDIRRSLKRDDGKEVPGEELHEILREIDTNMNGQVELDEYLQMMSAIKSGHVTYSRFAR

MAELEEQKHEKEMLKKKISVERSGGGV

>Amel_XP_006564634

MASLKLLVGGASAIGASALTSYLLMSDNTVHADVCNPRPLKRPLPTREEQVKTLKNTSEYDVLIIGGGAT

GAGCALDACTRGLKTALIEGDDFSSGTSSRSTKLIHGGVRYLQKAIMHVDVEQYKMVKEALQERASMLHS

APHLAHPLPIMLPVYTWWQIPYFWVGIKMYDFVAGRKTVKSSYFLSKRNALELFPMLKGDKLTGAIVYYD

GQQDDARMNLAIALTASRHGATVVNHVKVVNLLKGLDKDGNRVLTGARVKDELTGEQWDVKAKAIINATG

PFTDHIRKMDDQNVKEICSPSSGAHIVLPGYYSPDQMGLLDPETSDGRVIFFLPWQKQTIAGTTDLPCEI

THNPRPTEDEIMFILREVKNYLNPDVEVRRGDVLSAWSGIRPLVSDPNKPDTQSLARNHIVHVSPTKLIT

IAGGKWTTYRAMAEETIDAAIKACDLKPERPCQTNGLLLEGAHGWSPTMYIRLVQDFGLECEVAQHLAKS

YGDRAFAVAKMASLTGKRWPIIGKKLHPEFPYIDAEIRYGVREYARTAIDMIARRLRLAFLNVQAAQEAL

PGIIDIMAEELHWSPEEKNRQTKEASEFLANEMGQMVNRASRDKIPINLTKEEIQLYIKRFGIIDKDNKG

YVSINDIRRGLKLFGDKEVPGEELHEILREIDTNMNGQVELDEYLQMMSAIKSGHVAYSRFARMAEMEEA

QHEKEILKKQISVERSGGGL

>Pcoq_MNCL01000004

LDYEQYRMVREALAERSIMLESAPHLTHPLPIMLPVYTYITFKKENFCISYELSFRWWQVPYYWFGIKCYDLIAGDRNVKSSYYLSKQGALELFPMLRKEKLCGAIVYYDGKPCFPSIGLLYK*SYISKGQQDDARLNLAIALTAARHGAAVANHVEVKELIKKKDKEGKMVLCGAKVIDHISGKEWTIKAKCIINATGPFTDLIRKMDDPKVRSICSPSSGAHIVLPGYYSPDQMGLLDPSTSDGRVIFFLPWQRQTIAGTTDAPCELTHSPSPTEDDIQFILTEIKHYLNDDVEVRRGDVLSAWSGIRPLVSDPNKEDTQSLARNHIVHVSPSKLVTIAGGKWTTYRAMAEHTIDAAIKGIEKFFRIF*PVAN*TLLLTACDLKPERPQAVTSRVKIEGAQGWTPTMYIRLVQDFGLECEVAQHLAKSYGDRAFAVSKMASLTGKRWPVIGNRIHPEFPYIDAEIRYGVREYACNAVDMIARRLRLAFLNVQAAQEALPGIVDIMAEELKWSKDEKE

>Cnas_XP_031636993

MAARFRNIGVTTIGACAGLGLAGWALNPFDKQFANVNAAAIATPRVKRKLPPRSEQVKTLQSGEEYDVII

IGGGATGAGCALDSITRGLKTALIEGDDFASGTSSRSTKLIHGGVRYLQKAILGADIEQYRMVKEALHER

ASMLHSAPHLTHPIPIMLPVYKWWQLPYYWVGIKCYDFVAGDRNVKSSYVLSKKDALELFPMLKGDKLVG

AIVYYDGQQDDARMNLAVALTAARQGATVVNHTEVTALLKKKDASGKEVLCGCKVKDTITNKEWEVKGKC

IINATGPFTDHIRKMDDPNVKTICCPSSGVHIVLPGYYSPQQMGLLDPSTSDGRVIFFLPWQNQTIAGTT

DLPCDVTHNPKPTEDEIEFILTEIKNYLNADVEVRRGDVLSAWSGIRPLVSDPNKEDTQSLARNHIVHVS

PSNLVTIAGGKWTTYRAMAEHTMDAAIKACNLKPEKVDCQTDGMLLEGAHGWTPTMYIRLVQDFGLECEV

AQHLAQSYGDRAFPVAKMASLTGKRWPIIGKKVHPEFPYIDAEIRYGVREYACTAIDMIARRLRLSFLNV

QAAQEALPTIVDIMAEELNWSKDEKERQIKSATEFLASEMGQIVNRASRDKIPINLSKDEIQLYIKRFQI

IDKENKGYVSINDIRRALKNFGDADISGEELHEILREIDTNMNGQVELDEYLQMMSAIKSGHVAYSRFAR

MAELEEEKHEVEKLKKKISVDRSGGGL

>Mdes_AEGA01031562

MAARFRKIGVTTVGGCAGAALATWALNSFDNKSPFAVSVFKKXNVNAAAIATPRVKRKLPPRTEQVKTLQSGEEYDVLIIGGGATGAGCALDSITRGMRGSVXKGLKTALVEGDDFASGTSSRSTKLIHGGVRYLQKAILGXSLFQADIEQYRMVKEALHERASMLHSAPHLTHPIPIMLPVYKXFRWWQLPYYWVGIKCYDFVAGDRNVKSSYVLSKKDALELFPMLKGDKLVGAIVYYDGQQDDARMNLAVALTAARQGATVVNHTEVTELLKKKDENGKMVLCGAKVRDTLTNKEWEVKAKCIINATGPFTDHIRKMDDPSVKTICCPSSGVHIVLPGYYSPQQMGLLDPSTSDGRVIFFLPWQNQTIAGTTDLPCEVTHNPKPTEDEIEFILTEIKNYLNADVEVRRGDVLSAWSGIRPLVSDPNKEDTQSLARNHIVHVSPSNLVTIAGGKWTTYRAMAEHTLDAAIKACNLKPIKEESQTDGMLLEGAHGWTPTMYIRLVQDFGLECEVAQHLAQSYGDRAFPVAKMASLTGKRWPIIGKKVHPEFPYIDAEIRYGVREYAMTAIDMIARRLRISFLNVQAAQEALPTIVDIMAEELGWSKEEKERQIKSATEFLATEMGQIVNRASRDKIPINLSKEEIQLYIKRFQIIDKENKGYVSINDIRRALKXVNDDSLHELLKEIDVSYNGRLELWDYFQVXMMSAIKSGHVAYSRFARMAELEEEKHEVEKLKKKITVDRSGGGL

>Smos_VUAH01006169

MAARFRKVGVTTLGACAGLGLAGWALNPFDRQYANVNAAAIATPRVKRKLPPRSEQVKTLQSGEEYDVIIIGGGATGAGCALDSITRGMQXGLKTALIEGDDFASGTSSRSTKLIHGGVRYLQKAILGXADIEQYRMVKEALHERASMLHSAPHLTHPIPIMLPVYKXPLSRWWQLPYYWVGIKCYDFVAGDRNVKSSYVLSKNNALELFPMLKGDKLVGAIVYYDGQQDDARMNLAVALTAARQGATVVNHTEVTSLLKKKNEEGKEVLCGVKVRDTITNKQWEVKGKCIINATGPFTDYIRKMDDPNVKTICCPSSGVHIVLPGYYSPQQMGLLDPSTSDGRVIFFLPWQNQTIAGTTDLPCDVTHNPKPTEDEIEFILTEIKNYLNSDVEVRRGDVLSAWSGIRPLVSDPNKEDTQSLARNHIVHVSPSKLVTIAGGKWTTYRAMAEHTMDAAIKACNLQPEKESCQTDGMLLEGAHGWTPTMYIRLVQDFGLECEVAQHLAQSYGDRAFPVAKMASLTGKRWPIIGKKVHPEFPYIDAEIRYGVREYACTAIDMIARRLRLSFLNVQAAQEALPTIVDIMAEELNWSKEEKERQIKSATEFLTNEMGQFVNRASRDKIPINLSKDEIQLYIKRFQIIDAENKGYVSINDIRRALKXSFGDAEVSGEELHEILREIDTNMNGQVELDEYLQVXMMSAIKSGHVAYSRFARMAELEEEKHEVEKLKKKITVDRSGGGL
