# Supplementary material for: Genomic signatures of globally enhanced gene duplicate accumulation in the megadiverse higher Diptera fueling intralocus sexual conflict resolution
Source: PeerJ. 2020 Oct 12;8:e10012. doi: 10.7717/peerj.10012 (PMC7560327; doi:10.7717/peerj.10012)
Supplement: Supplemental Information 9 [file peerj-08-10012-s009.zip › Trxr protein sequences 2020.docx]

>Dmel_Trxr-1

MAPVQGSYDYDLIVIGGGSAGLACAKEAVLNGARVACLDFVKPTPTLGTK

WGVGGTCVNVGCIPKKLMHQASLLGEAVHEAAAYGWNVDEKIKPDWHKLV

QSVQNHIKSVNWVTRVDLRDKKVEYINGLGSFVDSHTLLAKLKSGERTIT

AQTFVIAVGGRPRYPDIPGAVEYGITSDDLFSLDREPGKTLVVGAGYIGL

ECAGFLKGLGYEPTVMVRSIVLRGFDQQMAELVAASMEERGIPFLRKTVP

LSVEKQDDGKLLVKYKNVETGEEAEDVYDTVLWAIGRKGLVDDLNLPNAG

VTVQKDKIPVDSQEATNVANIYAVGDIIYGKPELTPVAVLAGRLLARRLY

GGSTQRMDYKDVATTVFTPLEYACVGLSEEDAVKQFGADEIEVFHGYYKP

TEFFIPQKSVRYCYLKAVAERHGDQRVYGLHYIGPVAGEVIQGFAAALKS

GLTINTLINTVGIHPTTAEEFTRLAITKRSGLDPTPASCCS

>Dvir_XP_002056889

MKPINTALPDKGARRTSQSGNRNPGKTLPQGRNSLESSTSDSGINVPPFNHPHCDRQAMYQQPVRKVKPL

RGNYDYDLVVIGGGSGGLSCAKEAVAHGARVACLDFVKPTPIGTKWGVGGTCVNVGCIPKKLMHQASLLG

EAVHEAAAYGWNVDDKIKPDWNKLVSSVQNHIKSVNWVTRVDLRDKKVEYINGLGSFVDPHTLSAKLKSG

DRTITAQTFVIAVGGRPRYPNIPGAVEYGITSDDLFSLDREPGKTLVVGAGYIGLECAGFLKGLGYEPTV

MVRSIVLRGFDQQMANLVAASMEERGIPFLRKTVPLSVEKQSDGRLLVKYENTETGEIDSDVFDTVLWAI

GRKGLVEDLNLHNAGVLTHKDKIQVDCEETTNVPHIYAVGDIIYGKPELTPVAVLAGRLLARRLYADSDL

RMDYADVATTVFTPLEYACVGLSEEDAVKTYGAEEIEVFHGYYKPTEFFIPQKSVRYCYVKAVAQRSGEQ

RVYGLHYLGPVAGEVIQGFAAALKSGLTIPTLMNTVGIHPTTAEEFTRLSITKRSGLDPTPASCCS

>Dmel_Trxr-2

MSTIKFLRSSTHNALRSSLGWCRLAASRPRYDYDLVVLGGGSAGLACAKE

AAGCGARVLCFDYVKPTPVGTKWGIGGTCVNVGCIPKKLMHQASLLGEAV

HEAVAYGWNVDDTNIRPDWRKLVRSVQNHIKSVNWVTRVDLRDKKVEYVN

SMATFRDSHTIEYVAMPGAEHRQVTSEYVVVAVGGRPRYPDIPGAVELGI

TSDDIFSYEREPGRTLVVGAGYVGLECACFLKGLGYEPTVMVRSIVLRGF

DRQMSELLAAMMTERGIPFLGTTIPKAVERQADGRLLVRYRNTTTQMDGS

DVFDTVLWAIGRKGLIEDLNLDAAGVKTHDDKIVVDAAEATSVPHIFAVG

DIIYGRPELTPVAILSGRLLARRLFAGSTQLMDYADVATTVFTPLEYSCV

GMSEETAIELRGADNIEVFHGYYKPTEFFIPQKSVRHCYLKAVAEVSGDQ

KILGLHYIGPVAGEVIQGFAAALKTGLTVKTLLNTVGIHPTTAEEFTRLSITKRSGRDPTPASCCS

>Dvir_XP_002047939

MLMFKALRNSRLPRSVLLLRFASSATTTSNCKYDYDLVVLGGGSGGLACAKEAVEYGAHVLCFDFVKPTP

AGTKWGVGGTCVNVGCIPKKLMHQASLLGEAVHEAVAYGWNVNDQNIKPDWKKLVKSVQDHIKSVNWVTR

VDLRDKKVEYVNSIGRFVDPHTIEYIQKQGERKQLTAKYVVVAVGGRPRYPSIPGATEFGITSDDIFSYE

KEPGRTLVVGAGYVGLECACFLKGLGYDPTVMVRSIVLRGFDRQMSEFLAAMMLERGIRFLNTTIPLAVE

RRADGKLLVKYQNTTTKTDGSDVFDTVLWAIGRKGLTEDLNLGAAGVQTYNDKIIVDHTEATNVPHIFAV

GDIIYGRPELTPVAILSGRLLARRLFAGSTQLMDYTDVATTVFTPLEYSCVGMSEEMAIEKMGAENIEVF

HAYYKPTEFFIPQKSVRHCYLKAVAEVSGDQKILGLHYIGPVAGEVIQGFAAALKSGLTVKTLLNTVGIH

PTTAEEFTRLSITKRSGRDPTPASCCS

>Aaeg_AAEL002886

MSLLVARRVGNLISVRYTRLLSTNTTLLAKENFDYDLVVIGGGSGGLACAKEAVQFGAKV

AVLDFVKPSPRGTKWGLGGTCVNVGCIPKKLMHQASLLGEAIHDAQPYGWKFAEPESVKH

DWATLTESVQNHIKSVNWVTRVDLRDKKVEYVNGLGYFKDAHNVVAVMKNQTERVLNTKN

VVIAVGGRPRYPNIPGALEHGITSDDIFSLPHEPGKTLVVGAGYIGLECAGFLKGFGYDA

TVMVRSILLRGFDQQMATMVGDAMVEKGIKFLHKTQPQSVEKQADGRLLVKYRSDDGTEG

SDVYDTVLFAIGRTACTDDLKLDQAGVVTAEGGKSDKLDVDSFETTNVPNIFAVGDVLYK

RPELTPVAIHAGRLLARRLFNNQTDIMDYADVATTVFSPLEYGCVGMSEENAEAKFGKDK

VEVYHAYYKPTEFFVPQRSVRYCYLKAVALLEGDQKVLGLHFLGPVAGEVIQGFAAALKS

GLTMKILKNTVGIHPTVAEEFTRLLITKSSGLDPTPATCCS

>Cpip_CPIJ005552

MSSPAVTTLNQLGIFHHLLLLLSNVCPLAMSDSGDHQNLTPHRHEENYDYDLVVIGGGSG

GLACAKEAIQFGAKVAVLDFVVPSPRGTKWGLGGTCVNVGCIPKKLMHQASLLGEAIHDS

QPYGWKFAEPASVKHDWATLTESVQNHIKSVNWVTRVDLRDKKVEYVNGFGYFKDAHNVV

AVMKNKTERVLSTKYVVIAVGGRPRYPNIPGAEEYGITSDDIFSLPQEPGKTLVVGAGYI

GLECAGFLKGLGYDATVMVRSILLRGFDQQMATMVGDAMVEKGIKFMHKTQPTSVEKQED

GRLLVKYASDEGVEGSDVYDTVLFAIGRTACTKDLKLDQAGIVTAEGNKSDKLDVTVQEQ

TNVDNVFAVGDVLYKKPELTPVAIHAGRLLARRLFNNQSDVMDYTDVATTVFSPLEYGCV

GLSEEDAEAKYGKENVEVYHAYYKPTEFFVPQRSVRYCYLKAVALLEGDQKVLGLHFLGP

AAGEVIQGFAAAVKSGLTMKILKNTVGIHPTVAEEFTRLLITKSSGLDPTPATCCS

>Agam_AGAP000565RA

MATAVLARPARSLINVVQCVRLIRTQATVMFAKENYEYDLVVIGGGSGGLACAKQAVQLG

AKVAVLDFVKPSPRGTKWGLGGTCVNVGCIPKKLMHQASLLGEAIHDSQPYGWQLPDPAA

IRHDWATLTESVQNHIKSVNWVTRVDLRDQKVEYVNGLGYFKDDHTVVAVMKNQTERELR

AKHVVIAVGGRPRYPDIPGAAEYGITSDDIFSLPQAPGRTLLVGAGYIGLECAGFLKGLG

YDVSVMVRSILLRGFDQQMATMVGDSMVEKGIRFHHRSRPLAVEKQPDGRLLVRYETVDE

AGTATNGEDVFDTVLFAIGRQAETGTLKLANAGVVTAEGGKSDKLEVDETDHRTNVPHIY

AVGDVLYRKPELTPVAIHAGRIIARRLFGGSEERMDYADVATTVFTPLEYGCVGLSEEAA

EAAHGKDGIEVYHAYYKPTEFFVPQRSVRYCYLKAVALREGNQRVLGLHFLGPAAGEVIQ

GFAAALKCGLTMQVLRNTVGIHPTVAEEFTRLAITKRSGLDPTPATCCS

>Tcas_XP_008191172

MALRLCFSTVIFRGVCKVKNSKFLVLQNVNYINSNIRWYSKSDTAEHDLNMTQCDGPEYDLVVIGGGSGG

LAAAKEAAGLGAKVAVLDYVTPSPKGTKWGLGGTCVNVGCIPKKLMHQAALLGEAIEDAKSYGWQFPQPE

NIKHDWASLRQAVQNHIKSVNWVTRVELRDKKVEYINGLGVFKDPHTVHTVTKQGERTLTSKYFLIAVGG

RPRYPNIPGAVEYGITSDDIFSLEEAPGKTLVVGAGYIGLECAGFLRGLGYDATVMVRSVVLRGFDQQMA

KLIASAMEEKGVKFLHKCLPKSVEKRSDNKLLVKWSNETGQEFEDVFDTVLFAIGRRALTRELHLDKAGV

KVAGDGEKIDAMNEQSNVPHIFAVGDVLYKKPELTPVAIHAGRLLARRLFGNSTVQMDYDNVATTVFSPL

EYGSVGISEETAIQRFGENNIEIYHAYYKPTEFFIPQRSIAHCYLKVVAKREGPQQVLGMHFIGPQAGEV

IQGFAAAMKCNLTVNALMSTVGIHPTIAEEFTRINITKRSGKDPNPASCCS

>Tcas_XP_969619

MAFSDKVLDLFQNFYKAKLLQLTHFLKDIRHSFRFYASARPEFDLIVIGGGSGGLAAAKEAAELGAKVAV

FDFIVPSARSLKWGLEGTCINIESIPKKLMHRVAILGEAVQDARSYGFQFPKMESLKHNWKGLRETVQNH

IKSINWVTKIELRDKRVEYINSMGVFHDPYTIEAKIKNEWKTFKAKYFLISVGGRPKYPDIPGAELGISS

DEVFGLENAPGKTLIIGAGYVGVECAGFLKGLGYDITVMVRSVVLRAFDQQMAKLVTESMVAKGVRFLHK

CVPTSIERSNGKMLLVKWIDETREEGCDEFQTVLFAIGREACIRALRLDKAGVSVVADGDKIETINEQTN

VPHIYAVGDVLYKKPDLTQVAIHAGKLLARRLFAKSTVLMDYDNIATTIFTPLEYGSVGLCEETAIERYG

EDNIEIYHAYYKPTEFFIPQKTNAHCYLKVVAKRGNQQKVLGMHFVGPQAGEVIQGFSAAIKCNLTVDNL

RNTVGIHPAIAEEFSRINLTKRLAKDPFPVASNS

>Amel_NP_001171496

MKSKKKISLCLNCRKKSQTMDSDLSDQETEDYAKSLNNLTEEQNNLTADQKFMYDLIVIGGGSGGLAAAK

EAVNFGAKVAVLDFVTPSPRGSTWGLGGTCVNVGCIPKKLMHQAALLGESIHDSVSYGWQLPDPKTIKND

WEALRTAVQNHVKSVNWVTRVELRTKKIEYFNALGYFKDQHTILGKLKNGEEKEFTAQNILIAVGGRPRY

PDIPGALEYGITSDDIFSLEKAPGKTLIVGAGYIGLECAGFLNGLGYDATVMVRSIVLRGFDQQMASTVA

QEMERRGVHFIYEAKPSKIEKQADGRLLVHWVDKDRQTHQDTFDTVLFAIGRKPLTEELKPENIGLKLVP

ETAKIDAIDEQTNVPNVYAVGDVLHKKPELTPVAIHAGRLLARRLFGNSTEQMDYVNVATTVFSPLEYGC

VGLSEEAAIAIHGNDKIEIYHAYYKPTEFFIPQKDVSNCYLKVIAFRNGDQRVLGMHFIGPNAGEVIQGF

AAAIKCNLTFPKLKDTVGIHPTVAEEFTRISVTKRSGLDPKPQSCCS

>Ccap_comp59608_c3

DYVKPSPVGSKWGLGGTCVNVGCIPKKLMHQAASLGEAVHESVAYGWQIPEPEKIKPDWSKLVQAVQNHIKSVNWVTRVDLRDKKVEYINGLGYFKDQHTIIAKMKNGTERSITAQNILIAVGGRPRYSPIPGAVEYGITSDDIFSLGREPGKTLIVGAGYIGLECAGFLKGLGYDATVMVRSVVLREFDQQMAEIIKDSMVERGIKFLFTTLPNSVEKQSDGRFQVKWTNSKTGEEGSDIFDTVLFAIGRKGLVDDLNLDAAGVEVKNDKIVASESEQTNIPHIYAVGDILHGKPELTPVAIHAGRLLARRLFSGSTQFMDYTNVATTVFTPLEYSCVGLSEEVALQKYGEENIEVFHGFYKPTEFFIPQKSVRYCYLKAIAERSGDQKVLGLHYVGPVAGEVMQGFAAAVKAGLTMKILLNTVGIHPTTAEEFTRLSITKRSGLDPTPATCCS

>Tdal_Td_comp162833

DYVIPTPIGTKWGIGGTCVNVGCIPKKLMHQAALLGESIHEAESYGWEIPDSQKIKPDWGKLVQSVQNHIKSVNWVTRVDLRDKNVEYVNGLGSFVDPHTISAKLKNGSERLLTGQNIVIAVGGRPRYPEIPGADLGITSDDLFSLDHAPGKTLVVGAGYIGLECAGFLNGLGYDATVMVRSIVLRGFDQQMANIVADAMVERGIKFLHKTIPKVVCRNGNGRYLVKYYNTETDVESSDEFDTVLWAIGRKGLVNELNLSNVNVETKADKIIVNNAEETNVPNIYAVGDIIHGRPELTPVAIHAGRLLARRIFAGSTQIMDYTDVATTVFSPLEYACVGMAEEDAIRQHGEDNIEVFHGFYKPTEFFVPQKSVRYCYLKAVALRSDDQKVLGLHYIGPVAGEVIQGFAAAVKSGLTMKILLNTVGIHPTTAEEFTRLSITKRSGLDPTPATCCS

>Dant_CL2228_Contig3

DYVTPTPLGTKWGIGGTCVNVGCIPKKLMHQASLLGEAIHEASAYGWEIPDKEAIKPDWNKLVQAVQNHIKSVNWVTRVDLRDKKVEYLNGLGSFKDPYTVVVKMKNNSERLITAKNIVVAVGGRPRYPDIPGAVEYGITSDDLFSLDRAPGKTLVVGAGYIGLECAGFLKGLGYDATVMVRSIVLRGFDQQMANIVADSMVERGIPFLHKTIPKSVEKTADGRFLVKYVNTETQEEGSDIYDTVLWAIGRKGLVDDLNLSAAGVKVKNDKILVNKAEQTNVANIYAIGDIIHGRPELTPVAIHAGRLLARRLFGNSKQIMDYTDVATTVFSPLEYASVGMAEEDAIKEFGEDNVEVFHGFYKPTEFFIPQKS

>Gmor_GMOY004864

MMTQTQPAAAAANDEEEDEHAKYGYDLIVIGGGSGGLACAKEAIVNGAKVACLDFVKPTP

HGSKWGLGGTCVNVGCVPKKLMHQASLLGEALSDAQAYGWEIPDAKNIKPDWGKLVQSVQ

NHIKSVNWVTRVDLRDKKIEYLNGLGSFRDSHTILVKMKNNTERTVSARNIVIAVGGRPR

YPNIPGAELGITSDDLFSLDKPPGRTLIVDIGLECAGFLRGLGYDATVMVRSIILRGFDQ

QMANMVADSMVERGVKFIHKTIPKSVEKTGHNTYLVKYINIETKEEGSDEYDTVMWAIGR

KGLLEDLNLTSIGIELKNDKILVNDAEQTNVSNVYAVGDITYGRPELTPVAIHAGRLLAR

RLFGNSTQLMDYCNVATTVFSPLEYACVGMSEEDAAQEYGEDNIEVFHGFYKPTEFFIPQ

KSVRYCYVKAVAERSGDQRVLGLHYLGPVAGEIIQGFAAAVKCGLTMKILMNTVGIHPTT

AEEFTRLNITKRSGADPTPASCCS

>Mdom_MDOA001306

MGIVLSRCRPQPRAIVDTNEYDYDLIVIGGGSGGLACAKEAVANGAKVACLDYVKPTPLG

TKWGIGGTCVNVGCIPKKLMHQASLLGESIHEATAYGWEIPNKEAIKPKWENLVQAVQNH

IKSVNWVTRVDLRDKKVEYINGAGSFKDPHTVVAKMKNGSERTLTGRNVVIAVGGRPRYP

DIPGAVEYGITSDDLFSLDKEPGKTLVVGAGYIGLECAGFLKGLGYDATVMVRSIVLRGF

DQQMANMVADSMVERGIPFLHKTIPKSVEKTPDGRLLVKYVNTETQEEGSDVYDTVLWAI

GRKGLVDDLNLGAAGIEVKADKIAVNEAEQTNVPHIFAVGDIIHGRPELTPVAIHAGRLL

ARRLFGGSKQIMDYTDVATTVFSPLEYACVGMAEEDAIQKFGEDNIEVFHGFYKPTEFFI

PQKSVRYCYVKAVAERSGDQKVLGLHYLGPVAGEVIQGFAAAVKSGLTMKILLNTVGIHP

TTAEEFTRLAITKRSGLDPTPASCCS

>Gmor_GMOY003575

MAKSRSDLQSWEYDLICIGVGSGGLACAKEAVKLGAKVACLDFVRPTPAGTRWGIGGTCV

NVGCIPKKHMHQAALLGNAIEDAPHFGWNVPKDYAINWKKMVTEIQNNIRSTNWLVKVEM

RTQNMTYFNGIGSFIDQNTIRVRMANGTEQQIRGKYILIAIGNRPVYPKIPGALEYGITS

DDLFSLSKPPGKTLCVGAGYVSMECAGFLNELGFNVTVMARAEILSTFDQEMATIVKEYM

ASRGVKFLQYRLPKSVELTANKQLLVKFRNLKNENIEEQGIFDTVLWAMGRRALLDDINF

SAVNITIDDHEIVVNDFEQTSVPNIFAVGDVVKGRPKLTPVAIKAGVSVARRLFGGEDSC

VNYNNIPTTLFTPLEYSFVGVTEEEALRQFDENDIDVHHGYYNPIEFILSKRPTAYCYIK

VITKRDESCRILGIHFIGPNAGEILQGFAVAMNCGLTLDTLFSTVSVNITNAEEVTKVFI

TKRSGLDPSKPLYFRIYSYLNKCKKKKIFKNMKKIQVGKHDNNYEYDLVCIGGGCGGLAC

ALKAVELGVKVACIDPQRGAAGIYTNVGITYKKHMHQACLLGREVCHAACYGWNVRNPRK

RSMNWQKLISKIKIATERVAKTDDRMKYIQFFNGFGSFVDAHSVKVKMTNKEELLVTGKY

ILVATGTRPVYPKIPGSLEYGITSDEFFSLPSPPTGKTLCVGGGVVCMEIAGILIGLGFN

VTVMARSKVLQAFGQEMVKLLMENMASRGVVFLQHCLPKSIALARCKRLLVSYNHSQKKD

DEEFGVFDRVIWAIGRRAPLDDINISVINPALNDSEIIVNEYEQTTVPNIYAIGDIASGR

PKLFPVAVAAGTLLAQRLFSGVRQAMNYENVAQAIYTPLEYSFVGLTEEEAEKRFDKNGI

VVHHGYYEPLEYALTHHSTNFCYMKVIAKCDEHRPVLGMHITGPNAGEIMQGFSAAINSG

LTLHTLFDTVGVCFTNAQNFTQLLNKKK

>Mdes_JXPD01005603

NYEYDLVVIGGGSGGLACAKEAVSHGAKVAVLDYVTPSPR-GTKWGLGGTCVNVGCIPKKLMHQAALLGEAVHEAAAYGWNVDPKSLKLDWSVLRQNVENHIKSVNWVTRVDLRDKXRKVEYINGLGYFKDKNTVVAVMKNKSERVLSTKYVVIAVGGRPRYPDIPGALEYGITSDDVFSLQNEPGKTLVVGASYISLECAGFLNAFGYDTTVMVRSIFLRGFDQQMAELVAGAAAEKGVKFLQKTVPDSIEKQDDGRLLVRFTSAE-GQQGSDVFDTVLFAIGRKALTDDLQLQAAGVKLAPGSHKIAVDDEERTNVENIFAVGDVLEGKPELTXARRLFTDSQQKMDYADVATTVFTPLEYGCVGLSEEAAIAKYNEKNITVYHAYYKPTEFFIPQKSVRYCYLKTVALASGDQKILGMHYCGPVAGEVIQGFAAALK

>Cnas_VYII01001024

AKEAVAHGAKVAVLDFVKPSPQ-GTKWGLGGTCVNVGCIPKKLMHQAALLGEAVHEAAAYGWNVDKDNLKLDWSVLRQNVENHIKSVNWVTRVDLRDKXRKVEYINGLGYFKDKNTVVAVMKNKTEKVLTTKYVVIAVGGRPNYPDIPGALEYGITSDDVFSLKTEPGKTLVVGAXSYIALECAGFLNAFGYDTTVMVRSIFLRGFDQQMAEMVAAAAAEKGVKFLQKTVPSAVEKTEDGRLLVRYTSAE-GQQGSDVFDTVLFAIGRKACTDDLQLQAAGVKLHPGSHKLAVNDIEQTNVENIYAVGDVLQGKPELTAVAIHAGRLIARRLFAGATQKMDYADVATTVFSPLEYGCVGLSEEDAIAKFGEDNVEVYHAYYKPTEFFIPQKSVRYCYLKAVAFKSGDQKVLGLHYVGPVAGEVIQGFGAAVKXRAGLTIKILLNTVGIHPTTAEEFTRLSITKSSGLDPTPATCCS

>Smos_VUAH01000003

EENFEYDLVVIGGGSGGLAAAKEAVAHGAKVAVLDFVKPTPK-GTKWGLGGTCVNVGCIPKKLMHQAALLGEAVHEAAAYGWNVDKDNLKLDWSVLRQNVENHIKSVNWVTRVDLRDKXRKVEYINGLGYFKDKNTVVAVMKNQTEKVLTTKYVLIAVGGRPNYPDIPGAIEHGITSDDVFSLKNEPGKTLVVGAXSSYIALECAGFLNAFGYDTTVMVRSIFLRGFDQQMAEMVAAASAEKGVKFINKAVPSAVEKTEDGRLLVRYSNAE-GQQGSDVYDTVLFAIGRKACTDDLQLQAAGVKLAPGSHKIAVDDEERTNVENIFAVGDVLAGKPELTXATQKMDYADVATTVFSPLEYGCVGLSEEAAVAKFGEDNVEVYHAYYKPTEFFIPQKSVRYCYLKAVAFRSGDQKVVGLHYIGPVAGEVIQGFGAAVKXAGLTIKTLLNTVGIHPTTAEEFTRLSITKRSGLDPTPATCC

>Pcoq_MNCL01000081

IQEAVSYGWQVDNQENIKPDWNALTEAVQNHVKSVNWVTRVDLRDKXKVEYINGLGAFKDPHTVVATLKNNTTRELTAKHIVIAVGGRPRYPDIPGAVEYGITSDDIFSLKKEPGKTLVVGAGCXIGLECAGFLKGLGYDATVMVRSIILRGFDQQMANIVRDAMIEKGIRFLNHCIPECVEKTADGKLLVKYMNNETMDVEYEEFDTVLWAIGRKGQIENLKLENAAVDLKADKVVVNEKDQTNVPHIYAIGDIIYGKPELTPVAIHAGRLLARRLFTSSTQIMDYTDVATTVFSPLEYGCVGLSEEIAIEKFGEDNVEVYHAYYKPTEFFVPQKSVRYCYLKAIALRSGDQKVVGLHYVGPVAGEVIQGFAVALKYVFLPNSKRN
