# Supplementary material for: Genomic signatures of globally enhanced gene duplicate accumulation in the megadiverse higher Diptera fueling intralocus sexual conflict resolution
Source: PeerJ. 2020 Oct 12;8:e10012. doi: 10.7717/peerj.10012 (PMC7560327; doi:10.7717/peerj.10012)
Supplement: Supplemental Information 9 [file peerj-08-10012-s009.zip › Tom20 protein sequences 2020.docx]

>Dmel_Tom20

MIEMNKTAIGIAAGVAGTLFIGYCIYFDKKRRSDPEYKKKVRERRRRNKK

TGTAKSGVPNLNDHEAIERYFLQEIQLGETLIARGDFESGVEHLANAIVV

CGQPARLLQVLQSSLPAQVFAMLIVKMQEFGNRAAEGNDGPIVLGQSSEQ

QLDGAKIIECSSGNASIDDLE

>Dvir_XP_002048011

MIEINKTAIGIAAGLAGTLFIGYCIYFDNKRRSEPDYKKKVRDRRRRNRKVGSAKQGMPNLNDHEAIERY

FLQEIQLGETLIARGDFESGVEHLANAIVVCGQPARLLQVLQSSLPAQVFAMLIIKMQEFGNRATESSDG

LVSGPRSSQNETSSNILETSGNGAIDDLE

>Dmel_Tomboy20

MIGVSGTFKVLAAISGILFMGYCVYFDKQRRSDPDFKRKLHERRIQRSLA

SVKSTASVSMSERDVEVYFMTQIHKGETLITNGDVEAGVEHLINAILVCG

QPSKLLQLLQSTLPMDIFTTMLIKMHAYEASQRCLPVLVDDEATSSL

>Dvir_XP_002053611

MIGILVNKTSLGVVAGLASAAFLGYCVYFDSKRRNHPEFKKNLYERRRRNRKSDENEGVPVLTDQRSIER

YFMQEIHKGELLITEGNFERGVDHLVNAIVVCNKPGKLLSVLQSTLPVEVFSLLVYKLNNYNINAQRPPS

MTDNLITGLVADDGSVE

>Dvir_XP_002057047

MLSISCRSLLAFAVGTAGAFILGFCIYFDQKRRSDPEYKKKVHARRQREQDKFKYRISSGDDIDPNSCML

DANNHTSLERCFLNEINLGEHLLIRGNMSEGLSHLANAIMMCAQPLPVLQTLKESLPERVFMPLIIKLQE

LQSSGSNTMPSSNKDNSTSSPDFS

>Dvir_XP_002057046

MSRNSLLALTAGTAGALLLGYCIYYDRKRRSDPNYKKKVHERRQKTNPLPFVCDYRGESTSDDTFELNDH

EVVQHYFQNEIKMAEDLFRQAKLDAGLVHLANAIMVCAQPVALLEAMKVALPERIFNLLLTKLPELQLPN

PLGETTGVDLALD

>Dvir_XP_XP_002055407

MLKFSGHKLILFGLAALAFVSYCIYFDHKRCKDPQYRRKVHERRQLEIPQSANCKLPLNAEVVEYFLRHV

YLGEAYVRRDDWDRAVHYFANAIMICADPHILLCKLQTVVPLELYKRIMDRVRLLVRTSDNWSKLSSSMA

ETYTTSPERTLDKTSSRTSCIRDCSQDN

>Ccap_XP_004537692

MNAINKTAIGIAAGVAGTLFIGYCIYFDSKRRADPEYKKKVRERRRRTRKNGNAGRSGIPNLNDHEAIER

YFLQEIQLGETLIARGEFETGVEHLANAIVVCGQPARLLQVLQTSLPAQVFAMLILKMQEFGNRANAEMS

DTPKIVAANSIGAADMPLGLDGAQSSVLIDDLE

>Ccap_XP_004531038

MNTINRTTIGIAAGVAGTLFIAYCIYFDGRRRADPDYKKKVHERRRRLRKNRHTAGHGIPNLSSQQAIER

YFLQELKMGEELIERQEYENAVAHFANAVLVCGDPGRLLEMLQHTMPAQVFAILIIKMQEIGNRARAEMN

EAAKAAGVKRMENADEDEDATDAALIDDLE

>Dant_Unigene1020

VMDVNKTAIGIAAGVAGTLFIGYCIYFDSKRRSDPEYKKKVRERRQKCKTGSTTRSGMPNLNDHEAIERYFLQEIQMGETLIARGDFESGVEHLANALVVCGQPARLLQVLQTTLPAQVFAMLILKMQEYGNRSNAETSDAPKIVASNPLVSEISSGGGISIDDLE

>Tdal_Td_comp147136

MIELSKTVIGIAAGVAGTIFIGYCIYFDSKRRNDPDYKKKIRERRRQHHTSGVTRGGLPNLNDHKAIERYFLQEIQLGETLISKGDYERGVEHLANAIVVCGQPAELLQVLQSSLPAQVFAMLIIKMQELGNRSNTESNEASKMISLDTAGDTSSTKFENSSDAVLIDDLE

>Ppap_FK813889

MEINKTTLGIAAGVAGTLFLGYCIYFDHKRRSDPDYKKKIRDRRRKAKKAAAGNRPEIPNLLDKDAVQRFFLQEIQMGEMLIANGHIEQGVEHLANAVVVCGQPTQLLQVLQQTLPAQVFTLLIHKMKEYRNKMEPQGTEGRV

>Aaeg_AAEL007392PA

MEISKTTIGIAAGVAGTLFLGYCIYFDHKRRKDPDFKKKLRERRKAKKAASASGGPRTTMPNMADHEEVQRFFLQEIQMGEALISSGDIENGVEHLANAVIVCGQPAQLLQVLQQTLPAQVFTLLITRMRQYGGQS

>Llon_EW987750

MEINKTTLGIAAGVAGTLFLGYCIYFDHKRRSDPEYKKKVRERRRKAKKAAAGRRPEIPNLVDKDAVQRFFLQEIQMGEMLISRGHIEQGVEHLASAVVVCGQPTQLLQVLQQTLPAQVLTLLIHKMKEYGNK

>Llon_EX212033

AGVAGTLFLGCFICFDHMRRSDPEYRKKVRERRRLVKKAPAGRRPEIPNLVHKDAVHRFFLQEIQMGEMLISSGHIERGVEHLTRAVVVCGQPTHLLPVLHQTLPAQVFTLLIHELRDY

>Gmor_GMOY002684PA

MLEINKTALSIAAGVAGTLFIGYCIYFDNKRRSDPDYKRKVRERRRKNRKTGTARSGLPSLNDHEAIERYFLQEIQLGETLIARGDFESGVEHLANALVVCGQPARLLQVLQTTLPAQVFGMLIMKMQEFGNKANNENNETPKIATSDKIAPDMSSVGLESSSDAVIIDDLE

>Gmor_ctg10012869

MLNINKTVLRIVAGAIGVACIGYCIYFDKKRRQDPDYKKKIRQRRKRNVKQDSARDGGMPNLNDHEAIERYFLQEIQLGETLIARGEFDAGVEHLANALIVCGQPARLMHVLQTTLPGQVFGMLLMKMQETSNENSEAPKNTNSGKLRPDPSSVALETTSEAIIIDELE

>Mdom_XP_005192265

MLEINKTAIGIAAGLAGTLFIGYCIYFDNKRRSDPDYKKKVRERRRKNRKNGNNARGGLPNLNDHEAIERYFLQEIQMGETLIARGDFGSGVEHLANALVVCGQPARLLQVLQTTLPPQVFAMLILKMQEFGNRSSGETEEVPKIVSANQSDMAPVGMNSSAGGVIIDDLE

>Agam_AGAP012339PA

MEISKTIGIAAGVAGTLFLGYCIYFDHKRRKDPDFKKKLRERRKAKKAAASAGPRTTIPNLTDHEEVQRFFLQEIQTGEALISAGDIENGVEHLANAIIVCGQPTQLLQVLQQTLPAQVFALLITRMRQYGNQAGESERSKI

>Cqui_CPIJ007967PA

MEISKTTIGIAAGVAGTLFLGYCIYFDHKRRKDPDFKKKLREICSSENILFWVEQVVEIQMGEALISSGDIENGVEHLANAVIVCGQPAQLLQVLQQTLPSQVFTMLINRMRQYGNQSADSD

>Rpro_RPRC003750PA

MTMVSKTALGIAAGIFGTLFVGYCVYFDRQRRKDPNFKKKLRERRKANKVKCAAAKEKTKYPDLRDHEAVQQFFIHEIQLGEELLTQGDVEGGVEHLGNAVSVCGQPNQLLQVLQQTLPPNVFHLLLERLPVVTQRIMDQPTGGPVGSSGNLVEDDVE

>Tcas_XP_968248

MEMITSRVALGIAAGICGTLFLGYCIYFDHQRHSDPDFKKKLHERRRAKKMAASSNKRTTVFPDMKDHEAVQRFFLQEIQLGEELLAVGDLENGVDHIGNAVAVCGQPNDLLNLLQQTLQPQAFHLLIQRLPAVAPRIVKATPTMQEEDVE

>Amel_XP_006565977

MTMISKAAVGIAVGIAGIFVGYCFYFDQKRRSDPDFKKKLRERRKAKKQAQNATSKIQDLKDHEVVQRFF

LQEVQLGEEMLSCGDIEGAVEHLGNAVAVCGQPAQLLQVLQKTLPPQIFHLLLQRLQPISQKLSTQIAMA

EEDVE

>Pcoq_ MNCL01000003.1

IEMNKTaigiaagvagTLFIGYCIYFDKKRRSDPEYXGTAKSGVPNLNDHEAIERXRYFLQEIQLGESLVATGDFERGVEHLANAIVVCGQPARLLQILQTTLPAQVFALLIQKMQXEYGTRSEETKE

>Mdes_gbAEGA01003790

MSLDISRTTIGIAAGIAGTIFLSYCIYFDKKRRNDPEYKKKVRERKXRRRTRQRKIGNSGSRVELPNLNDHEAVQRXRFFLQEIQAGEALISSGDIEQGVIHLANAVVVCGXQPTQLLQVISISILQVLQQTLPSQVFTLLIQRMREYGNKAPNATEDTSSRNKITEELDYDLE

>Cnas_XP_031629449

MNLDISRVTLGIAAGIAGTIFISYCIYFDKKRRGDPGFKKRLYERRQKAKECPTGKSGSRTELPNLNDHEAVQRFFLQEIQAGEALISSGDFEQGVTHLANAVVVCGQPTQLLQVLQQTLPSQVFTLLISRMREYGNNKQPAPVEVHNEQRHTFDLDDDLE

>Smos_VUAH01006149

MNLDISRTTLGIAAGIAGTIFLSYCIYFDKKRRSDPEYKKKIRERXRFFLQEIQAGEALISSGDIEQGVTHLANAVVVCGQPTQLLQVSTMTLSTNEXFTLLIQRMREYG-NKAAAAEESAPRNIITEDIDDDLEMNLDISRTTLGIAAGIAGTIFLSYCIYFDKKRRSDPEYKKKIRER
