# Supplementary material for: Genomic signatures of globally enhanced gene duplicate accumulation in the megadiverse higher Diptera fueling intralocus sexual conflict resolution
Source: PeerJ. 2020 Oct 12;8:e10012. doi: 10.7717/peerj.10012 (PMC7560327; doi:10.7717/peerj.10012)
Supplement: Supplemental Information 9 [file peerj-08-10012-s009.zip › porin protein sequences 2020.docx]

>Dmel_porin_AAF53022

MAPPSYSDLGKQARDIFSKGYNFGLWKLDLKTKTSSGIEFNTAGHSNQES

GKVFGSLETKYKVKDYGLTLTEKWNTDNTLFTEVAVQDQLLEGLKLSLEG

NFAPQSGNKNGKFKVAYGHENVKADSDVNIDLKGPLINASAVLGYQGWLA

GYQTAFDTQQSKLTTNNFALGYTTKDFVLHTAVNDGQEFSGSIFQRTSDK

LDVGVQLSWASGTSNTKFAIGAKYQLDDDASVRAKVNNASQVGLGYQQKL

RDGVTLTLSTLVDGKNFNAGGHKIGVGLELEA

>Dmel_Porin2_AAF53020

MAAKTPTYPDLGKLARDLFKRGYHPGIWQIDCKTLTNSGIEFFTTGFASQ

DNSKVTGSLQSKYKIEDQGLTLTERWNTENWLFGEIMHRDKLAQGLMLAV

EAKFQPGSNEADGKFKMGYAQDNFNFLADIGLNSEPILNCSLVVGHKEFL

GGVGTEFDVGNTELKGWKVALGWTNETATLHGELKNGDTWLASLFYKASE

KIDAGIEVTKGAGGGEAAEGEQQGGDVVVNLGMIYHLEEDALVRAKVNNL

VELGLGYEQKLRDGITASISAVLDCNNFKDGNHRFGVGIALQC

>Dvir_XP_002052597

MTDHGKKYWTYNVLREKLRRKEMAPPSYSDLGKQARDIFSKGYNFGLWKLDLKTKTSSGIEFNTAGHSNQ

ESGKVFGSLETKYKVKDYGLTLTEKWNTDNTLFTEVAVQDQLLEGLKLSLEGNFAPQSGNKNGKFKVAYG

HENVKADSDVNIDLKGPLINASAVLGYQGWLAGYQTAFDTQQSKLTTNNFALGYSTKDFVLHTAVNDGQE

FSGSIFQRTSDKLDVGVQLSWASGTSNTKFAIGAKYQLDDDASVRAKVNNASQVGLGYQQKLRDGITLTL

STLVDGKNFNAGGHKIGVGLELEA

>Dvir_XP_002052598

MAPPPVYTDLGKLARDLFRRGYHPGLWQLDCKTMTSSGIEFFTTGFASQDASKVMGSLQSKYNIEDYGLT

LTERWNTDNLLYGEIAQKDKLVEGLLLALEGRFQPSSGDKEGKFMARYAQESYNILGKIDIKSDPLVGLS

LVLGHKEFLGGAAVDCDINGGNVSWKVALGWTNESTTLHAELINAEGWLLSLFHKANEQIDAAVEIGKAA

AEAAAEEGQEPTESELNIGVGMIYHLAGDALIRAKINNNAELGLGYQQKLREGITMSISTVLDCKNITEG

NHKFGVGLSLEC

>Ccap_XP_004524766

MAPPSYPDLGKQARDVFSKGYHFGLWKLDCKTKTPSGIEFNTAGHSNQESGKVFGSLETKYKVKDYGLSL

TEKWNTDNTLFTEVSVQDKLLEGLKLAFEATFAPQSGNKTGKFKAAYGHENVKVDSDVNVDLNGPLINAS

AVLGYEGWLAGYKTAFDTQNNSLKTNNFALGYAAKDFVLHTAVNDGQEFTGSIFQKCTPKLDVAVQLSWT

SGGNNTKFGLGGKYLLDDDISVRAKVNNASQVGLGYQQRVRDGITVSLSALIDGKNFNAGGHKIGVALEL

EA

>Aaeg_gi_78216392

MAPPAYADLGKQARDVFNKGYHFGLWKLDVKTKTNSGVEFNTSGSSNQDNGKVFGSLETKYKVKEYGLNFSEKWNTDNTLTSEVSVENQLVKGLKLSFDGSFAPQTGSKTGRFKTAYSHDKVRVDADVNVDLAGPLVNASGVFNYQGWLAGYQVAFDSQKSKVTANNFALGYSTGDFVLHTNVNDGREFGGLIYQRCNDRLETAVQLSWASGSNATKFGLGAKYDLDKDACVRAKVNNQSQIGLGYQQKLRDGVTLTLSTMIDGKSFNTGGHKIGVALELEA

>Agam_AGAP009833

MAPPSYSDLGKQARDVFNKGYHFGLWKLDVKTKTNSGVEFSTSGHSNQDTGKVFGSLETKYKVKEYGLNFSEKWNTDNTLTSEVSVENQLVKGLKVSFDGMFVPHTGSKTGRFKTAYSHDRVRVDADFNVDLSGPLVNASGVAAYQGWLAGYQVAFDSQKSKITANNFALGYSAGDFVLHTNVNDGREFGGLIYQRCNDRLETAVQLSWASGSNATKFGMGAKYDLDKDACVRAKVNNQSQIGLGYQQKLRDGITLTLSTLVDGKNFNAGGHKIGVALELEA

>Cpip_gi_145648988_gb_DS231823

MAPPAYSDLGKQARDVFNKGYHFGLWKLDVKTKTSSGVEFNTAGHSNQDNGKVFGSLETKYKVKEYGLNFSEKWNTDNTLTSEVSIENQLVKGLKLSFDGSFAPQTGXXXSKTGRFKTAYSHDKVRVDADVNVDLAGPLVNASGVFGYQGWLAGYQVAFDSQKSKVTANNFAVGYSTGDFILHTNVXXXFSLPLSSNDGREFGGLIYQRCNDRLETAVQLSWASGSNATKFGLGAKYDLDKDACVRAKVNNQSQIGLGYQQKLRDXXXGITLTLSTLIDGKSFNTGGHKIGVALELEA

>Llon_LLOTMP006374

MAPPQYSDLGKQARDIFNKGYHFGLWKLDVKTKTNSGVEFSTSGNSNQDSGKVFASLETKYKLKEYGLTFTENWNTDNTLLTEVTLQDKLLEGLKLTLDCSFAPQSGXXXNKTARVKAAYCHDYVHLNSDVNVDLAGPLVNAALVFGYQGWLAGYQSSFDTQKSRLTANNFALAYSTGDFVLHTNVNDGQEFGGSVYQKVNDKLDAAVQLSWSSGSNATKFGIGAKYNLDKDAAVRAKVNNSSQIGLGYQQKLRDGITLTLSTLIDGKNFNAGGHKVGLALELEA

>Gmor_GMOY003090

MAPPSYSDLGKQARDIFGKGYHFGLWKLDCKSKTSSGIEFSTSGHSNTESGKVFGSLETKYKVSDYGLTLTEKWNTDNTLFTEVAVQDKLLEGLKLAFEGSFAPQSGNKSGKFKVGYGHDNVKIDSDMNVDLSGPLVNASAVLGYEGWLAGYQTAFDSQNTKLTTNNFALGYATKDFVLHTAVNNGEEFSGSIFQKCNDNLDIGVQLSWASGSNNTKFGLGAKFQLDKDAALRAKVNNACQVGLGYQQKLRDGITLYLSTLVDGKNFNAGGHKIGVALELEA

>Mdom_MDOA014768

MAPPSFSDLGKQARDIFGKGYHFGLWKLDCKTKTPSGIEFSTAGHSNQESGKVFGSLETKYKVNDYGLTLTEKWNTDNTLFTEVAVQDKLLEGLKLAFEGTFAPQTGNKTGKFKVAYGHENVKIDSDVNVDLSGPLINASAVLGYEGWLAGYQTAFDTQHAKLTTNNFALGYTTKDFVLHTAVNDGQEFSGSIFQKCSDKLDVGVQLSWTSGSNNTKFGLGGKFQLDKDAALRAKVNNACQVGLGYQQKLRDGITLTLSTLIDGKNFNAGGHKIGVALELEA

>Ppap_PPATMP003321

MAPPAYSDLGKQARDIFNKGYHFGLWKLDVKTKTNSGVEFNTSGNSNQDSGKVFASLETKYKLKEYGLTFTENWNTDNTLLTEVTLQDKLLEGLKLTLDCSFAPQSGXXXNKTARVKAAYCHDYVHLNSDVNVDLAGPLVNAAAVFGYQGWLAGYQTSFDTQKSRVTANNFALAYSTGDFVIHTNV

>Tcas_gi_158703262_gb_CM000283.2

MAPPPYSDLGKKAKDVFGKGYHFGLIKLDCKTKTGSGVEFNTGGVSNQESGKVFGSLETKYKVKEYGLTFSEKWNTDNTLATEVAIQDQLLKGLKLSSDLTFSPQTGSKSARVKTAFTNDRVALNCDVDLDSSGPLIQAAAVVGHQGWLAGYQTAFDTQKSKLTKNNFALGFSTGDFILHTNVDDGQEFGGSIYQKLSPKLETGIQLAWSAGSNNTKFGIGAKYDLDQDAAIRAKVNNSSQIGLGYQQRLREXXXGVTLTLSALIDGKNFNNGGHKIGLAVELEA

>Amel_gi_318074899_gb_GL630235

MAPPSYNDLGKSARDLFSSGYHFGLIKLDVKTKTKSGVEFSSGGVSNQDTGKVFGSLETKYNIDDYGLKFSEKWNTDNTLATDITFADKLLKGLTLGYGCTFSPQTGKTGKLKTAYKHDNVSAAADFDLSLSTGPLVNASTVVGYQGGWLAGYQACFDTQRNKLTKNNFALGYTASDFTLHAAVNNGCDFSGLIYHKVKPELEGAINLEWNSSNNVTQFGIATKYNLDNDASIRAKVXXXNSNLQVGLGYQQKLRDGXXXGVTLTLSTNIDGKNFGSGGHKIGLALDLQA

>Tdal_comp143986_c0

MAPPSYSDLGKQARDIFGKGYHFGLWKLDCKTKTASGIEFNTVGHSNQESGKVFGSLETKYKVNDYGLTLTEKWSTDNTLFTEVSVQDQLLEGLKLSFAGTFAPQTGNKEGKLKASYGHENVKFDSDVNIDLKGPLINSSLVLGYQGWLAGYQTAFDTQNSKLAANNFALGYSTKDFVLHTAVNDGQEFSGSIFQRCSDKLDCGVQLSWATGGNNTKFGIGGKYQLDKDASVRAKVNNASQVGLGYQQKLRDGVTLTLSTLIDGKNFNAGGHKIGVALELE

>Dant_Unigene694

MAPPSFSDLGKQARDIFGKGYHFGLWKLDCKTKTASGIEFNSGGQSNQESGKVFGSLETKYKVSDYGLTLTEKWNTDNTLYTEVAVQDKLLEGLKLAFEGSFAPQSGNKSGKFKVAYGHENVKIDSDVNVDLNGPLINASAVLGYEGWLAGYQTAFDTQNAKLTTNNFALGYTTKDFVLHTAVNDGQEFAGSIFQKCSDKIDVGVQLSWTSGTNNTKFGLGTKYQLDHDAALRAKVNNNCQVGLGYQQKLRDGITLTLSTLIDGKN

>Mdes_gi_309241399_gb_GL501425

MAPPTYSDLGKQARDVFNKGYNVGVWKLDVKTKTNTGVEFTTSGHSNQESGKVFGSLETKYKVSEYGLTFSEKWNTDNTLSTDVTHTDKLLKGLKLTFEGTFAPQSGNKNGKVKVAYGHDLVQVNSDVNLDLAGPVVNASAVVGYEGWLAGYQTAFDTQKGKLVTNNFALGYTNNDLAVTTNVXDGQEFNGSIFHKVNSDLNCGVQLAWTSGSNVTKFGIGAQYALDKDASVRAKINNDSHIGLGYQQKLRPGVTLSLSTLLDGKNFNAGGHKIGLGLELEA

>Cnas_XP_031629667

MAPPSYADLGKQARDVFNKGYNFGVWKLDVKTKTNTGVEFSTSGHSNQESGKVFGSLETKYKVPEYGLTF

SEKWNTDNTLFTDITHNDKLLKGLKLTFEGTFAPQSGNKTGKVKASYGHDLVTVNSDVNLDLAGPVVNAS

AVVGYEGWLAGYQTAFDTQKTKLVTNNFALGYTNKELAVTTTVKDGQEFNGSIFHKVNDDLNAGVQLAWT

SGSNATKFGIGAQYCLDKNSSVRAKISNDSLIGLGFQTKVRPGVLLALSTLVDGKNFNTGGHKVGIALEL

EA

>Smos_VUAH01000005

MAPPTYADLGKSARDVFNKGYNFGVWKLDVKTKTDTGVEFSTSGHSNQETGKVFGSLETKYKVPEYGLTFTEKWNTDNTLFTDVSHNDKFLKGLKLTFEGTFAPQSGNKTGKVKASYGHDLLQVNSDVNLDLAGPVINASAVVGYEGWLAGYQTAFDTQKTKLVTNNFALAYTNKELAVTTTVXKDGQEFNGSIFHKVNDDLNAGVQLAWTSGSNATKFGIGAQYSLDKNSSVRAKISNDSLIGXGLGFQTKIRPGVLLALSTLVDGKNFNAGGHKVGIALELEA

>Pcoq_MNCL01000088

MAPPSYSDLGKQARDIFSKGYHFGLWKLDCKTKTDSGIEFTTSGHSNQDSGKVFGSLETKYKVKDTGFTFTEKWNTDNTLFTEVAVQDQFLEGLKLSFQGTFAPHTWYDFCVNVLGYNXNINSSYSIKTGKFKIGYGHENVKVDSDVDVDLSGPLINAAAVFGYQGWLAGYQTAFDTKNAKLTTNNFALGYTTGDFTLHTNVXDDGQEFAGSIYQKCSPQLDCGVQLSWSSDSNETKFGIGAVYSLDEDASLRAKVDNSSQIGXGVVLALSTLIDGKNFNAGGHKIGVALSLEA
