# Supplementary material for: Genomic signatures of globally enhanced gene duplicate accumulation in the megadiverse higher Diptera fueling intralocus sexual conflict resolution
Source: PeerJ. 2020 Oct 12;8:e10012. doi: 10.7717/peerj.10012 (PMC7560327; doi:10.7717/peerj.10012)
Supplement: Supplemental Information 9 [file peerj-08-10012-s009.zip › SdhC protein sequences 2020.docx]

>Dmel_SdhC

MYALSSSLIRSPALRQGLQMAAASRPVSMKVVSVAETQKDESFFEKNERLGRELSPHLTIYQPQLTSMLSICHRGTGLALGVGVWGLGLGALISSHDISHYVTMVEGLQLSGATLTALKFIIAYPAGYHTANGIRHLLWDTGRFLKIKEVYSTGYAMVATSFVLSAILALL

>Dmel_CG6629

MNTCRSLARGLNICSPQLRHLLAQKPFNSARLLATKAPKDHKASGPTTIGPSGDILVPPVTLKVIPFRMP

PDLPYDDRNMLLGRQLSPHLSIYKIQLTSTLSAFLRISGFVLAVFVWFVGISGLCLQGDMEGFIKKVEKC

DCHGMVTMAKVMVTMPFAYHTVAGTRHLIWYLNKFLTIPEIYATGYVAVALTIALSAFLLAVKVGEKVKE

EVVDLTKTKKGQKAKKEAPKDAKKDAPKDTKKEPKKDAKDKKKDEEGKSAK

>Dvir_XP_002053422

MYALSSTLVRSPALRQGLRVAAATRQVSLKVVSVGSTIKDESFFEKNERLGRQMSPHLTIYKPQLTSMLS

IMHRGTGLALGIGVWGLGLGALIASQDIGHYVTMVEGLQLSGAALTAIKFIIAYPVGYHSANGIRHLIWD

TGRFLKIKEVYSTGYVMVATSVVLTAILALL

>Dvir_XP_002053434

MYSVYRNFGQLQRGAYRAHITLCNHRINVKPLILQTVHFSKSKGSDGEVRLGNDGKKLADGKKPPMSKKG

PVGPPKIDMRIIPASYHNLSYEELNKKLGRAMSPHLSIYKKQLTSVMSIFLRISGFILGIGIWTIGLTGL

LCDIDVNAMAEKIEKCDCSRTVFNMLKLFIIIPFAYHIVAGTRHLIWHLNVFLSKREIYATGYAAIVLTF

ILAAALAGIDVQEKTNELSKVSNDGEITLELKTLLNEETEEIDEHELDNEMTEADAKEV

>Mdom_MDOA011810

MFAITRSLVRSPALRQGLQMAQAQNLRDVSMKVVPAASTVKNETFFDKNNRLQREMSPHL

TIYKPQLTSMLSITHRGTGIALTAGVWALGLAALTSPQDIANYASVIEGLHLSAGTLTAL

KFMIAYPLAFHTANGVRHLLWDTGRFLKIKEVYSTGYAMVGVSFALAAILAML

>Gmor_GMOY011667

MYSFLRFLFFRYAITRSLTRSTVFRQSLSLSEACTGRHVAMKIVPVDTVTKKETFFEKNARLGREMSPHLTIYQPQLTSLLSVTHRGTGMVLTAGVWALGLAALISPQDIGNYASVIEGLHLGGGTLTFLKFMFAFPLAFHTANGVRHLLWDTGRFLKIKEVYSTGYVMVGMSFVLAAMLAMI

>Gmor_GMOY011672

MNSVKIKSRGSKVEKAKRMSLLTRACFLKRAIVCCPFKGISRWTKSGGATLIKMKVVPAPKTVWKTYEQKNKDVKRDLSPCLSIYRPQVTSSLSIILRVSGVALGFLFWGLGLFSLISDHQAEELVQYAEDLKWPGWFWATARWFVAFPFAFHYCNGIRHMCFMAGSFLDIRQIYATGYIVFIIATMLTVYLAMYNEIEEFKVKHSEE

>Ccap_XP_004518508

MYAITRSLIRSSALRQGIQKMQIAAPARQVSLKVVPAAEAIQAETFDEKNQRLGRELSPHLTIYKPQLTS

MLSITHRGTGFALTGYAWALGLGALMSSHDISHYVTIIEGLHLGSATLVALKFALAFPLAFHSCCGVRHL

LWDTGRFLKITEVYLTGYVTLGISIVLSAILACL

>Dant_Unigene1196

MYAVTRSLVRSPALRQGLQMAQVQNSRQVTMKVVSVGSTIKDETFTDKNIRLGREMSPHLTIYKPQLTSLLSVTHRGTGCALSVAVWGLGLAALTSPQDIANYASVIEGLQLSAGTLVALKFIIAYPLAFHSANGVRHLLWDTGRFLKIKEVYSTGYAMLGASFVLAGILAML

>Tdal_comp158719

VTIKIKPATSLKKESYDEKNTRLGREMSPHLTIYKPQLTSMLSITHRGTGFALGIMAWGLGLGALISSHDACHYFTMMEGLHLSGFTLGTVKALIAFPFAYHTCNGVRHLLWDGGLFLKLSEVYTTGYAMLG

>Tcas_XP_972413

MAAIFRLGNRQILSQLRNEKLGMLSLVRPVTLKAQPATKEIDIGHDERNMQLGRPQSPHLTIYSFQLTSM

LSITHRATGMMLAGYAIMWGTGAVVLPDTIPHYLDALQQAHVGGFVLSMGKFMLAFPMCYHFWNGIRHLA

WDLGRFLTIKEVYATGYAMLALTVASAIALTSM

>Tcas_XP_972464

MSAIFRLANRQTALKFKTNFGLLSLVRPVTLKPQPAPKEEDLGHDARNMKLGRPQSPHLTIYAPQMTSML

SISHRATGMILTFYTIVLGTGAIILPDSIETYLNALEEYHVGGVVLSVGKFILAFPLTYHYWNGIRHLAW

DIGLFLSLKEVYMTGFAMLGLAYGSAAVLSVI

>Amel_XP_006564913

MALCYMRLLSRRCIDPCTFRNFYTCSSRNIAVSKPLFKETTICETHDEKNLRLKRPLSPHLTIYQIQLTA

FLSITHRTTGMILSSYAMLFGIGTLLIPGGIPCLIEIISELGLSAPVLFVGKTLLALPATYHTFNGLRHL

AWDLGMFLTIKEVYSTGYAVIALSAISAIALAAL

>Cqui_XP_001859594

MAGLILRSACRRALLQSQNAASPMLAARTVVLKTVQADNRPGESHDDRNARLQRPQSPHLTIYSFQLTAV

LSITHRMTGMALAGYATALGLGALAMPHDATHYLTMLEGLSAPTLMALKFTMAYPFSYHTVNGVRHLFWD

LGKFLSIKEVYTTGYTMLLVSGVLAAGLTFL

>Agam_XP_311387

MAASLLLRNACRRTLLQGYQSTNAALPLLAARTIVLKPVQADVRPGESHDDRNARLKRPQSPHLTIYSFQ

LTSMLSITHRFTGLALTGYITALGLGALAMPHDATHYLTMLEGLSAPTLIALKFTMAYPFAYHTVNGVRH

LFWDMGKFLTIKEVYTTGYTMLGVSGVLAGLLTAL

>Pcoq_MNCL01000221_1

LSIKVVP-APAPPQESHDERNMRLKREMSPHLSIYKPQLTSVLSITHRXHDFSHYITMIEGLHLGGGSLLAIKFILAFPVSFHTCNGVRHLFWDMGKFLKIRDVYSTGYMMLASSIGLTALLAIL

>Pcoq_MNCL01000221_2

KNQCYKRPMSPHVEIYKYELHMAASFLHRXIILSFYFLTFGIGTLVLPSEFECYVIWLENMNLPGWFIYFGKFLLALPFTIHSFGGLRHLIWDTGAMLNTMSSVWTLDNATFALGLGISLILPLI

>Mdes_AEGA01015301

NFIRLLGSRSPWLLNAGYNLNRNALFTTSAQNCIKMKIVQPNDLDKLSYDEKNALLKREQSPHLTIYKFQLTSMMSISHRITGLILTGYAGALCLGAILPPNDVSYWVTAIESMQLSSPTLAFIKFYIALPATYHTFNGIRHLAWDNGKNLQLKDVYRTGYIVIALSVISALGLA

>Cnas_XP_031641004

MAMALNFGRILSSRTPLLFKLNVGQNLYQNALFTTSANKCVQLKIVKPTNHDNLSYEEKNSLLKREQSPHLTIYKPQLTSMISISHRISGLALTGFAACLCIGAVLPPNDISYWLTAIEGMQLNAATLAFIKFYLALPVTFHTLNGVRHLFWDNGKFLNLNEVYKTGYTVLGLSVVSALILAAM

>Smos_VUAH01006165

FCRVLSSRAPLLFKVNAGQKLYQNALFTTSAQKYVQLKIVKPNDLEKLSYEEKNSLLKREQSPHLTIYKPQLTSMLSISHRISGXIAGLILSGFATALWFGAVLPPNDISYWVTAAEGVQLSAATLAFIKFYVALPVTFHTLNGVRHLLWDNGKFLQLTQVYKTGWTVVG
