# Supplementary material for: Genomic signatures of globally enhanced gene duplicate accumulation in the megadiverse higher Diptera fueling intralocus sexual conflict resolution
Source: PeerJ. 2020 Oct 12;8:e10012. doi: 10.7717/peerj.10012 (PMC7560327; doi:10.7717/peerj.10012)
Supplement: Supplemental Information 9 [file peerj-08-10012-s009.zip › Ucp4 protein sequences 2020.docx]

>Dmel_Ucp4A_AAF48769

MAAKTDESSPAVASSTSSNPAPSSGRHQLRPVKFDYADSFACTYIVSVVA ASIAELATYPLDLTKTRLQIQGEGAAHSAGKSNMQYRGMVATAFGIAREE GALKLWQGVTPALYRHVVYSGVRICSYDLMRKEFTQNGTQALPVWKSALC GVTAGAVAQWLASPADLVKVQIQMEGRRRLMGEPPRVHSAGHAFRQIVQR GGIKGLWKGSIPNVQRAALVNLGDLTTYDTIKHLIMNRLQMPDCHTVHVL ASVCAGFVAAIMGTPADVVKTRIMNQPTDENGRGLLYRGSVDCLRQTVSK

>Dmel_Ucp4B_AAF52314

MGKGVNTVFRPAEWDNSEEKERPKLEYLVTNKKTPPVELYLTAFASACSA EIVGYPFDMCKTRMQIQGEIASRVGQKAKYRGLLATAMGIVREEGLLKLY GGISAMLFRHSLFSGIKMLTYDYMREKMIVPDEDGRPQLSFLGSCISGVL AGATASVLTNPTELIKIQMQMEGQRRLRGEPPRIHNVLQALTSIYRTGGV VGLWKGTVPNTWRSALVTIGDVSCYDFCKRFLIAEFDLVDNREVQFVAAM TAGVADAILSLPADVVKSRIMNQPTDEQGRGIHYKGSLDCLSRLVREEGF LAMYKGFIPYWMRVGPASVVFWMTFEQIRRFRGSEGY

>Dmel_Ucp4C_AAF52313

MDKAERDYWHLRSLEIEEEPRFPPTNVADPLTARNLFQLYVNTFIGANLA ESCVFPLDVAKTRMQVDGEQAKKTGKAMPTFRATLTNMIRVEGFKSLYAG FSAMVTRNFIFNSLRVVLYDVFRRPFLYQNERNEEVLKIYMALGCSFTAG CIAQALANPFDIVKVRMQTEGRRRQLGYDVRVNSMVQAFVDIYRRGGLPS MWKGVGPSCMRACLMTTGDVGSYDISKRTFKRLLDLEEGLPLRFVSSMCA GLTASVLSTPADVIKSRMMNQPVDESGKNLYYKNSLDCVRKLVREEGVLT LYKGLMPTWFRLGPFSVLFWLSVEQLRQWEGQSGF

>Dvir_XP_002059558

MPAPQVIAAAAAAITTASVPSSDASANAATNATAANSSSSSSSSGSSSSAVNSGRHQLRPVKYDYADSFA

CTYIVSVVAASIAELVTYPLDLTKTRLQIQGEAASVATIASSSISSSSAKANMQYRGMVATAFGIVREEG

AIKLWQGVTPALYRHVVYSGVRICSYDLMRKEFTKNGSQALPVWKSALCGVTAGAVAQWLASPADLVKVQ

IQMEGRRRLMGEAPRVHSAGHAFRMIVQRGGIKGLWKGSIPNVQRAALVNLGDLTTYDTIKHLIMRRLQM

PDCHTVHVLASICAGFVAAIMGTPADVVKTRIMNQPTDELGRGLLYRGSLDCLRQTVAKEGFVALYKGFL

PCWIRMAPWSLTFWLSFEQIRKMIGASSY

>Dvir_XP_002051379

MASRDELETTEKLLSAQRSRLYYLSTNRKTPFVELYASSVLSACSAEMFCYPLDVLKTRMQIQGENASKT

YSNIKYSGMLGTARSIIREEGLAKLYGGVSAMVLRHAIYTGLKMYMYDTLREALIIDKDGKLELTFLRGA

ICGIVAGAGATLLTSPTDLIKVQMQMESKRRLMGEPPRIHNVYQALTSTYKAGGIVALWKGTLPNAWRSG

LVTLGDVSFYDLSKRQLMDILNMPDNLLIQFLGAMIAGLSGAVLSTPADVVKSRMMNQPVDKAGRGLHYR

GTMDCFTKLVQQEGFMAMYKGFLPYWLRVGPWTLIFWLTFEQIRSLNGDAGY

>Dvir_XP_002051833

MGKPDRWPLEGYSAVEVNNVVNYETSRLVEICITSFLSAVNADLIVYPLDVTKTRLQIQGEHGNPYANMA

KYRGLFGTALGVIKEEGFLKLYSGFSALVLRHSFVSGLKIGSYDYLRSKWSVRTDDKVTISMPCTMLAGI

VSGALSTIASNPLDLVKLQMQMESKRILLGMPPRSTGIMQALQFIYSQGGLRSLYRGLGPNIMRASLFSL

GGISFYDLGKRNIKKLLNSEENLLVQFLAAMVAGFFCSALSCPADVVKSRIMNQPVDDQGRPLRYKNSID

CLQQLVKEEGPMAIYKGFMPYWIRCGPWFLVFWMSFEGIRRFNGYGTF

>Dvir_XP_002051381

MNKRAVFQDYPNRQSWPESFEPATQSHQLLLVYINTFLGATIAELVSYPLDVTKTRLHLQGEAADKLAAG

KPIRGMFGTLFGMMREEGFRGTYGGLSAMVIRNLMFNAPRVVVYDYVRQQLIYVDENGNQVLSMMRGFFA

GCLAGCMCQAIANPLDIVKIRMQMEGRQRSLGYPVRVSNVKQALESIYAQGGVKSLWKGVGPSCLRATLM

TAGDTACYDLSKRHLIALLHLEDGRCLQFLASVSAGLAASILSTPADVVKSRIMNQPYNDEGQGQHYKNA

FDCYHKLITQEGFLAMYKGFLPCWLRIGPWSIIFWIAFEQLRRVQGQTGF

>Dvir_XP_002051380

MDRDENNYWHMKPLYDKAILRSVDNSQEQLSVRNLLQLYVNTFIGANFAEACMYSLDVSKTRMQVHGEEA

KRTGSKPRNMFRTLYGIWVEEGPRNLYAGFSAMVVRNFIFNSLRVMLYDVFRRRFIYEDAQNVQSIKIHH

AFLCGSAAGCIAQALANPFDIVKVRMQMEGRRLLMGMEPRTTNFVSDLAEIYRKSGVVGMWRGVGPSCTR

ACLMTAGDVGAYDLCKRNLKKYLGMEEGIPLRFASSMVAGLVASVLSNPADVIKSRMMNQPIDENGKGLY

YKNSVDCVVKLVRDEGFLNLYKGLIPCWLRLGPWSVLFWLSVEQLRVWEGQTGF

>Ccap_XP_004536134

MQSQTQSKSTAEPPSPPPPPPTGSTTRNQLRPVKYDYADSFACTYVVSVVAASVAELTTYPLDLTKTRLQ

IQGEAAASELKTGNGTKTKYRGMVATAFGIVREEGTMKLWQGVTPALYRHVVYSGVRICSYDFLRRKLGS

DNNGIITLPLWKSALCGVSAGAVAQWLASPADLVKVQIQMEGKRRLMGEPARVHGAAHALKEIVRRGGVA

GLWKGSIPNVQRAALVNLGDLTTYDTIKHMIMIKLNMPDCHTVHVLSSICAGFVAAIMGTPADVVKTRIM

NQPTDEKGRGLLYKGSVDCLRQTVNKEGFGALYKGFLPCWIRMAPWSLTFWLSFEQIRSMIGVAGVVASL

HAFFK

>Dant_Unigene15766+Unigene19127

KPQLQYRGMMATALGIVREEGTLQLWQGVTPALYRHIVYSGVRICTYDYLRKEMAKNEGGKDIALPLYKSALCGVCSGGLAQWLASPADLVKVQIQMEGxxxKVQIQMEGKRRLMGEAPRVHGAGHAFKEIIRRGGAKGLWKGSIPNVQRAALVNLGDLTTYDTIKHMIMNKLHMPDCHTVHVLASICAGFVAAVMGTPADVVKTRIMNQP

>Tdal_Td_comp158093

RNQMRPVKFDYADSFACTYIVSVFAASIAEVATYPLDLTKTRLQIQGEAVTETMKIQNATIQPKMQYRGMLATAFGIIKEEGPLKLWQGITPALYRHVVYSGVRICSYDFLRKEYGQTENSNNITLPLWKSAICGVLAGAIAQWLASPADLVKVQIQMEGKRRIMGAPPRVLSVAHAFRQIVHQGGLKSLWKGSIPNVQRAALVNLGDLTTYDTIKHSIMHKLNMPDCHTVHVLASICAGFVAATMGTPADVVKTRIMNQSTDDKGRGLLYKRSIDCLQQTIVK

>Aaeg_AAEL007235PA

STRTEMRPVKYHYADSFWCTYLISVFAASIAETVTYPLDLTKTRLQIQGEATAVTGAIKKLKYRGMLATASGIIREEGALKLWQGVTPALYRHIVYSGVRIVTYDNLRKKLRNGNNDFALWQSALAGVGAGGLAQWLASPADLVKVHIQMEGKRRLLGLEPRVHGAAHAFREIVSRGGIAGLWKGSVPNVQRAALVNLGDLTTYDTVKRFVMKKSGLPDCHLVHIISSICAGLVAATMGTPADVVKTRVMNQPTDINGKGLLYKGSLDCLQQTIGK

>Llon_LLOTMP010089PA

RTEMRPVKYHYADSFLCTYVVSVIAANMAELVTYPLDLTKTRLQIQGEATAGGARAISSPQYRGMLATALGIVKEEGALKLWQGVTPALYRHVVYSGVRIVTYDTLRKSMMGHGDTSLPLHKSALCGVTAGALAQWMASPADLVKVQCQMEGKRRLQGLEPRVHGAAHAFREIVKRGGVRGLWKGSVPNVQRAALVNLGDLTTYDTVKRAIMRRTGLPDCHLVHIASSVCAGLVAATMGTPADVVKTRIMNQPIDTTGRGLLYKGSVDCLVQTVSK

>Cqui_CPIJ004342PA

RTEMRPVKYHYADSFWCTYLISVFAASIAETVTYPLDLTKTRLQIQGEAAATAAAGGLKKTKYRGMFATASGIIREEGALKLWQGVTPALYRHVVYSGVRIVTYDGLRRKLRNGNNDFALWKSAVAGVGAGGLAQWLASPADLVKVHIQMEGKRRLMGLEPRVHGAAHAFREIVARGGIAGLWKGSIPNVQRAALVNLGDLTTYDTVKHIVMKRTGLPDCHMVHVISSICAGLVAATMGTPADVVKTRVMNQPTDLHGNGLLYKGAIDCLQQTIGK

>Ppap_PPATMP006241

RTEMRPVKYHYADSFLCTYVVSVIAANMAELVLPDDCVVAVTYPLDLTKTRLQIQGEAAVSEGSRIVTRTQYRGMLATALGIVREEGALKLWQGVTPALYRHVVYSGVRIVTYDTLRKSMMAANHETNASLPLHKSALCGVTAGALAQWMASPADLVKVQCQMEGKRRLLGLEPRVHSAAHAFREIVKRGGVRGLWKGSVPNVQRAALVNLGDLTTYDTVKRAIMRRTATMGTPADVVKTRIMNQPVDSAGRGLLYKGSVDCLMQTVSK

>Mdom_MDOA013911

VQYRGMMATAAGIVKEEGPLKLWQGVTPALYRHIVYSGVRICTYDFLRKELGKNESGQNVALPWKSALCGVSSGGLAQWLASPADLVKVQIQMEGKRRLMGEPPRVHGAAHAFREIVRRGGIKGLWKGSIPNVQRAALVNLGDLTTYDTIKHMIMNKLHMPDCHTVHVLASICAGFVAAFLGTPADVVKTRVMNQPTDEKGRGLLYKGSIDCLRQTVNK

>Agam_AGAP009603PA

TGEAAAAASDGDTTMQQTAAATGRTEMRPVKYHYADSFWCTYLVSVFAASIAETVTYPLDLTKTRLQIQGEAAATAVDAEGALKYRGMFATATGIIREEGALKLWQGITPALYRHLVYSGVRIVTYDALRKKLRNGKETFSLWQSALSGVGAGALAQWLASPADLVKVHVQMEGKRRAMGLEPRVHSAAHAFREIVSRGGVFGLWKGSVPNVQRAALVNLGDLTTYDTVKHFIMHKTGLPDCHVVHIMSSICAGLVAATMGTPADVVKTRIMNQPTDSSGRGLLYKGSIDCLQQTIGK

>Tcas_XP_008196933

MPQIPPPKEHKYVDSLWCMYMVSVVGAWNAELVTYPLDLAKTRLQIQGEVANTKDASMVKAPYRGLFRTAVGIVSEEGFLKLWQGAYAALYRHLFYSGTRIVTYKHLKDKFFDSGTEQYFPVWKSALCGVTAGAFAQYIASPADLLKVQLQMEGKRKIMGLPPRVNGLFDAFRKTVETAGVRGLWKGSVPNVQRAALVNLGDLTTYDSAKRFILRNTTLEDNHLVHCLASSMAGLVAALMGTPADVIKTRVMNQPMDEQGRGLLYKSSIDCFKKSVQNEGFGALYKGFLPIWLRMAPWSLTFWLSYEEVLRLLGAEQF

>Pcoq_Ucp4_3_119-223_369103_369417

YSGVRIVTYDTLRKKMGATVTTSLPIWQSAICGVTAGALAQWMASPADLVKVQCQMEGKRRLMGLEPRVHGAGHAFKEIINRGGIKGLWKGSVPNVQRAALVNLG

>Pcoq_Ucp4_2_141_229_361411_362019

PLKVWEAAICGAIAGGVAQWIATPTELVKVQLQMEGKRVLMGLEPRYRSVGDAFRQIYIKSGIRGLWAGAAPGVIRAMCVNVGK

>Pcoq_Ucp4_1_130_298_358814_359437

LYQQFRGTIDRPLKVWEATLCGISAGAIAQWICTPAELVKVQYQMEGRRVLLGEKPRFKSFTDACAQIYRTGGIRGLWLGASPAVIRAMCVNIGDLTAYDVSKRFIHEKTGLHPRSVVLHFLASSVAGFVGAILTTPADLVKSRIMNQPVDEHGKRGLIYKGPLDCLEKTL

>Gmor_GMOY007874

MVEKKPIVTDTKTPLTENVEKRFIDTIPCTYILSVLSATAAELITYPLDLTKTRLQIQGELAKTQEVKAMHRGMLATAFGVVKEEGPFKLWCGMSSIIYRHTIYSGVRVCTYDYLRTTFGHDNLPVWKAALFGVLSGCFGQWLSNPADLVKVQMQMEGKRRLMGEAPRFLSFHQAFVDIYRRGGIVGLWQGSVPGLQRAALVNLGDLTAYDMTKRFLIHKMEMEEGPLAHILASLASGFVAAVAATPADVVKSRIMNQPTDERGRQVSGSLDCFKKTVEAEGYMGLYKGFVPHWMRLGPWTLTFWITFEQLRSILGGAAF

>Gpal_GBRX01008108_GLOS_DERE_GG19134_1758

MVEKKPIVTDTKTSLTENVEKRFIDTIPCTYILSVLAATNAELITYPLDLTKTRLQIQGELAKAQEVKVNLHYNYNVLSVIHVTLIMLA-AKYRGMLATAFGIVKEEGPLKLWYGVSSVIYRHMIYSGVRVCTYDYLRTTFGHDNLPVWKSALFGLLSGCFAQWLANPADLVKVQMQMEGKRRLMGEPPRILSSHQAFVDIYKRGGIVGLWQGSVPSVQRAALVNLGDLTAYDLAKRFLIHKMEMKEGPLAHMLASFVAGFVAAVAATPADVVKSRVMNQPTDERGRGIIYKGSLDCLKQTVSAEGYMGLYKGFVPHWIRLGPWALTFWVTFEQLRSILGGAAF

>Mdes_gb_AEGA01022594

STANTMTISRNEMRPVKYHFADTFWCTYCRYFMKVKVVLLEIFVFLTKKIMLRLKXISVVSANIAEMVTYPLDLTKTRLQIQGESNKLKA-GNSATNKARYVNMIIERCXQQIPYRGMVSTAVGIVREEGTLNLWRGVLPALYRHVLYXSGVRIAIYDQFRKSLDVSENHSGLPLWEAALCGVVAGGVAQWXITNRMASPADLVKVHVQMEGRRRLLGFEPRVHGASDAFKQIVARGGINSLWKGSIPNVCRAALVNLGDLTTYDFTKRKIMQKTKLPDCHTVHIMSSVCAGLIAATMGTPADVVKTRIMNQPTDEYGRGISYKNSMDCLRKTVGKEGFLALYKGFLPVWIRMAPWSLTFWLSFESIRKNLGANGY

>Cnas_XP_031634097

MPAPLATAAPPSISRNEMRPVKYHYADTFWCTYIISVVSANIAELATYPLDLTKTRLQIQGEGSKLNGNG

LAAAKKIQYRGMVATACGIVREEGATKLWQGVTPALYRHVVYSGVRIAIYDRMRKNLDVSEDHSGLPLWE

AALCGITAGGIAQWMASPADLVKVHVQMEGRRRLMGLKPRVHGASDALKQILHRGGVKALWKGSIPNVYR

AALVNLGDLTTYDFAKRNIMNKTKLPDCHTVHIMASIMAGLVAATMGTPADVVKTRIMNQPTDEMGRGLL

YKNSLDCFRQTVSKEGFFALYKGFLPVWIRMAPWSLTFWLSFETIRKQLGANGY

>Smos_VUAH01000011

MPAPLATTAPPSITRKEMRPVKYHYADTFWCTYIISVVSANIAELXATYPLDLTKTRLQIQGEGKKLKANDLATTNKVRFRRLFAPLLFISISEIYIIYWLGSIQRNGCHRLWHXVQYRGMVATVCGIVREEGAMKLWQGVTPALYRHVVYXIFSGVRIAIYDRMRKTLDVSEDHSGLPLWEAAICGVTAGGLAQWXMASPADLVKVHVQMEGKRRLMGLKPRVHGSSDALKQILKRGGVKALWKGSIPNVYRAALVNLGDLTTYDFAKRNIMNKTKLPDCHTVHIMASICAGLVAATMGTPMDVVKTRIMNQPTDEKGRXRGILYKNSLDCFRQTINKEGFFALYKGFLPVWIRMAPWSLTFWLSFESIRKTLGANGY
