# Supplementary material for: Genomic signatures of globally enhanced gene duplicate accumulation in the megadiverse higher Diptera fueling intralocus sexual conflict resolution
Source: PeerJ. 2020 Oct 12;8:e10012. doi: 10.7717/peerj.10012 (PMC7560327; doi:10.7717/peerj.10012)
Supplement: Supplemental Information 9 [file peerj-08-10012-s009.zip › Hsp60 protein sequences 2020.docx]

>Dmel_Hsp60

MFRLPVSLARSSISRQLAMRGYAKDVRFGPEVRAMMLQGVDVLADAVAVT

MGPKGRNVIIEQSWGSPKITKDGVTVAKSIELKDKFQNIGAKLVQDVANN

TNEEAGDGTTTATVLARAIAKEGFEKISKGANPVEIRRGVMLAVETVKDN

LKTMSRPVSTPEEIAQVATISANGDQAIGNLISEAMKKVGRDGVITVKDG

KTLTDELEVIEGMKFDRGYISPYFINSSKGAKVEFQDALLLLSEKKISSV

QSIIPALELANAQRKPLVIIAEDIDGEALSTLVVNRLKIGLQVAAVKAPG

FGDNRKSTLTDMAIASGGIVFGDDADLVKLEDVKVSDLGQVGEVVITKDD

TLLLKGKGKKDDVLRRANQIKDQIEDTTSEYEKEKLQERLARLASGVALL

RVGGSSEVEVNEKKDRVHDALNATRAAVEEGIVPGGGTALLRCIEKLEGV

ETTNEDQKLGVEIVRRALRMPCMTIAKNAGVDGAMVVAKVENQAGDYGYD

ALKGEYGNLIEKGIIDPTKVVRTAITDASGVASLLTTAEAVVTEIPKEDG

APAMPGMGGMGGMGGMGGMGGMM

>Dvir_XP_002058167

MFRLPVSLARTSISRQLAMRSYAKDVKFGAEVRAMMLQGVDVLADAVAVTMGPKGRNVIIEQSWGSPKIT

KDGVTVAKAIELKDKFQNIGAKLVQDVANNTNEEAGDGTTTATVLARAIAKEGFEKISKGANPVEIRRGV

MLAVDTVKDNLKTMSRPVKTPEEIAQVATISANGDQAIGKLISDAMKRVGRDGVITVKDGKTLIDELEVI

EGMKFDRGYISPYFINSSKGAKVEFQDALLLLSEKKISSVQSIIPALELANSQRKPLVIIAEDIDGEALS

TLVVNRLKIGLQVAAVKAPGFGDNRKSTLTDMAIASGGIVFGDDADLVKLEDVKISDLGQVGEVVITKDD

TLLLKGKGKKDDVQRRVDQIKEQITDTTSEYEKEKLQERLARLASGVALLRVGGSSEVEVNEKKDRVHDA

LNATRAAVEEGIVPGGGTALLRCIEKLDSVATTNEDQNLGVDIVRRALRMPCMTIAKNAGVDGAMVVAKV

ETQSGDYGYDALKGEYGNLIEKGIIDPTKVVRTAITDAAGVASLLTTAEAVVTEIPKEDAAPGMGGMGGM

GGMGGMGGMGGMM

>Dvir_XP_002052822

MFRSYVREAVRSSRAFARAYSKDVAFGAEARARMLRGVDMLTDAVAVTMGPKGRSVILERPWTSPKITKD

GVSVARAISLKDQHMQLGARLVQDVADNTNQAAGDGTTTATVLARCIAKEGFQHITRGANPVEIRRGIML

AVDHVKRKLKAMSRAVETREEIEQVATISANGDAEIGRLIADATDRVGRTGTITVKEGKRLKDEMEVLQG

LQFDKGYISPFFVNTPKGAKVEYANAYVLITLKKIKSLKQIVRGLEQSLRQRRPLLIIAEDIDGEALNAL

VLNRLKVGLQVCAVKAPAYGEYRKEMLGDIAAATGATVFGDDINYAKIEEAKIEDFGQVGELVVSKDSTM

LLQSQGKPELLKRRIQELEDELRDPATKPEQKERLRARISVLTNGVAVIHIGGTSEVEVGEKRDRVNDAL

NATRAAIEEGIVPGGGTALLRCIPSLQRLEPDNMDLKNGINIVCSAMRIPCMTIAANAGVDAATVVARVL

NGEGDFGYDAMKGEYGNLFEKGIIDPTKVVRTAIQDAAGVASLLSTTEVVITEVRNQDPLGALAGDGGGL

EEALGGLDLGGMGGMEALSALGGMGGMGGMGDMGMGGMGMGMGGMSKAEEMNQAVQSIAGMEDVTVHDID

SSQL

>Dmel_Hsp60D

MLSRLGRSGRAGVRTFANDIRFGAEARCLLMQGVNVLANAVATTLGPKGR

NVLIEQLLISPRITKDGITVANNVQLGNRRQDMGVQLLRQATNNTNNKVG

DGTTTATILARGIACQGMHVLRQSKVNVQLLREGILEGSRAVCDALGEMS

QSVDTIGQVEAVAKVALNGDERLAELIGDIILELGDSGVILLKESHSPFD

EAKIQEGITIASGYYSPFFAKQSHTLELENCLLLLTLAKIDQVEQILPAL

ELARLKERPLLIIAKNFGSDLLKILVLNNLQGRVQVCAVKAPSFGDEQCE

EMEDIAFATGGHLLEDASSLADLSEEDLGEVMEAVVDAKETHLLQPINVN

EEQVQCRIQDIRELIDEAFTDVELDRLKTRLGRLQGHLATIFVGGTSELE

VSERKDRFNDALHAVRVAISDGVVPGGGTAYLRCIPVLDELPPTDIMELQ

VGREIVKDALRLPCYTIARNAGVDPNEVLRRVLKGSGNYGYDAAAGEFGD

LVVRGIVDPTKVLQSAMTSAAGIASLLATTEVLITKQPTKPKIPKNQVTK

DLAKLVGM

>Dmel_Hsp60B

MFRSCVPKAITSSRCFARMYSKDVRFGSGVRAMMIRGVDILADAVAVTMG

PKGRSVIVERPWTSPKITKDGFTVARSIALKDQHMNLGAKLVQDVADNTN

ESAGDGTTTATVLARAIAKEGFNQITMGANPVEIRRGVMLAVDVVKDKLK

EMSKAVETREEIQQVATLSANGDTEIGRLIGEATDKVGPRGTITVKDGKR

LKDELNIIQGLRFDNGYVSPFFVNSSKGSKVEFANALVMISLKKITGLSQ

IVKGLEQSLRQRRPLIIIAEDISGEALNALVLNKLRLGLQVCAVKSPSYG

HHRKELIGDISAATGATIFGDDINYSKMEEAKLEDLGQVGEAVISKDSTM

LLQGKPKTGLLEMRIQQIQDELAEKQIKPEQRDRLRQRLSALTKGVAVLH

IGGGSEVEVNEKKDRVVDALNATRAAIEEGIVPGGGTAFLRCIPYLQELK

TESADLQKGVDIVCNALRMPCQTIAQNAGVDGPMVVAKVLNGSEDYGYDA

MGDEYCRLVEKGIIDPTKVLRTAITDAAGVASLLSTTEVVITDSRNDDLL

SKLSGAGGGMDDGLDMNMGGLEELAALSGLGGMGGMGGMGGMGGMGGMGG

GFGGMGAGGGMSASASNDGPTAEEMNEMVKAIPGMEQVEVRDIDSGMM

>Dmel_Hsp60C

MMRMFRYTNTLQRTAKISHVLWARNYAKDVRFGPEVRAMMLQGVDVLADA

VAVTMGPKGRNVIIEQSWGSPKITKDGVTVAKSIALKDKFQNIGAKLVQD

VANNTNEEAGDGTTTATVLARAIAKEGFEKISRGASPVEIRRGVMLAIET

VKDNLRRLSRPVNTPEEICQVATISANGDKSVGNLISEAIKKVGRDGVIT

VKDGKTLCDELEVIEGMKFDRGYISPYFINTSKGAKVEFQDALLLFCEKK

IKSAPSIVPALELANAQRKPLVIIAEDLEAEALSTLVVNRLKVGLQVCAV

KAPGFGDNRKENLMDMAVATGGIVFGDEANMVRLEDIKMSDFGRVGEVVV

SKDDTMLLKGKGQKAEVEKRVEGLREAIKESTSSYEKEKMQERLARLSSG

VALLRVGGSSDVEVSEKKDRVIDALNATRAAVEEGIVPGGGTALLRCIQK

LNDLKGANEDQNMGIEIIRRALRMPCLTIAKNAGVDGAMVVAKVEILDGD

YGYDALKGEYGNMIERGIIDPTKVVRTAISDAAGVASLLTTAEAVVTELP

LEEAAAAGAAAGLGALGGMGMGGMGM

>Dvir_Hsp60C

MLRMLTNSTLPRGSRIACQLAGRELWRGYAKDVKFGPEVRAMMLQGVDVLADAVAVTMGPKGRNVIIEQS

WGSPKITKDGVTVAKSIALKDKFMNIGAKLVQDVANNTNEEAGDGTTTATVLARAIAKEGFEKISRGANP

VEIRRGVMLAIDSVKVNLRKMSRPVNTPEEIAQVATISANGDKSVGNLISEAIKKVGRDGVITVKDGKTM

NDELEVIEGMKFDRGYISPYFINSSKGAKVEFQDALLLFCEKKIKTAASIVPALELANAQRKPLVIIAED

VEGEALSTMVVNRLKVGLQVCAVKAPGFGDNRKETLADMAIATGGLVFGDEANMVRLEDIKASDFGRVGE

IVVTKEDTMLLKGHGQRTMIEKRLENLREAIKESTSNYEKEKMQERLAKLSSGVALLRVGGSSDVEVGEK

KDRVNDALNATRAAIEEGIVPGGGTALLRCITKLNDLKGINEDQNMGIEIIRRALRMPCLTIAKNAGVDG

AMVVAKVEILDGDYGYDALKGEYGNMIERGIIDPTKVVRTAIIDAAGVASLLTTAEAVVTELPLEDANPM

GGMGGMGGMGGMGGMGGMGGLGM

>Ccap_XP_004529412

MLRLPLRFARANIHRQLAFRGYAKDVKFGPEVRALMLQGVDVLADAVAVTMGPKGRNVIIEQSWGSPKITKDGVTVAKSIELKDKFQNIGAKLVQDVANNTNEEAGDGTTTATVLARAIAKEGFEKISKGANPVEIRRGVMLAVDSVKDHLKAMSRPVNTPEEIAQVATISANGDHEVGNLISEAMKKVGRDGVITVKDGKTLSDELEVIEGMKFDRGYISPYFINSSKGAKVEFQDALVLLSEKKISSVQSIIPALELANSQRKPLVIIAEDIDGEALSTLVVNRLKIGLQVAAVKAPGFGDNRKSTLTDMAIASGGIVFGDDANLVKLEDVQINDLGKVGEVVITKDDTLLLKGKGTKEDITRRVDQIKDQISETTSEYEKEKLQERLARLASGVALLRVGGSSEVEVNEKKDRVNDALNATRAAVEEGIVPGGGTALLRCIPILEGLKGSNEDQNMGIEIVRRALRMPCMTIAKNAGVDGAMVVAKVETKDGDFGYDALKAEYGNLIEKGIIDPTKVVRTAITDASGVASLLTTAEAVVTEIPKDDAAPGMGGMGGMGGMGGMGGMGGMM

>Dant_Unigene1143

MFRLPVTLARSSIARQLAVRSYAKDVKFGAEVRAMMLQGVDVLADAVAVTMGPKGRNVIIEQSWGSPKITKDGVTVAKSIELKDKFQNIGAKLVQDVANNTNEEAGDGTTTATVLARAIAKEGFEKISKGANPVEIRRGVMIAVETVKDNLKAMSRPVSTPEEIAQVATISANGDKAIGDLISEAMKKVGRDGVITVKDGKTLIDELEVIEGMKFDRGYISPYFINSSKGAKVEFQDALLLLSEKKISSVQSIIPALEMANQQRKPLVIVAEDIDGEALSTLVVNRLKIGLQVAAVKAPGFGDNRKSTLTDMAIASGGIVFGDDANLVKLEDININDLGKVGEVVITKDDTLLLKGKGKKEDIQRRVDQIKDQIADTTSEYEKEKLQERLARLASGVALLRVGGSSEVEVNEKKDRVHDALNATRAAVEEGIVPGGGTALLRCIAKLETLKGQNEDQNMGIEIVRRALRMPCMTIAKNAGVDGAMVVAKVENKEGDWGYDALKGEYGNLIEKGIIDPTKVVRTAITDAAGVASLLTTAEAVVTELPKED

>Tdal_Td_comp155730

MLPLYRSYLRAVINRHIIVRHFVKNIRFVPEIRKLMSKEVGIFAKPVQLTSDRRGRNVIIKKPGKFPIITSSDHTVNKSIALAAKQNAGELLVQNMVNSANGKLDDKTFTTTVADQVKEKMGLKKLCTAVEPVKMHRGFILAVNTAKQILKAISHPISIKKGIAEVVTISVSGDKSIASLIYEALAKVKSNGAIIVEYGDSLTDKLELTKCLKFESGHKSMVYKNSVKGDRIQMQDEFILFGEKDGIVPALKLPYKQRQPFDSIAEGICGQAVRRLAINRLQYPMPQSAVRASCSGNAMREMLIYVAVFCAGIDFGHKARLYTLEAILFNDLCKIGKVIVSLLKSKGNKVEFQDHIGDKKQQIILTKPILEIDKLQGRLRSPTNDVAFLRICDSSEVNNNGKMIYGINALNMTCLCTEKPIVSDEGSELLSYIPTLNGLKGLNEDQKLGMEIVHLALRIASTRIAKNTGADGAIVVALAENGFADYGHDALYDEYGDVIEKGRIDPTKVLRTVFPVATGMGSLLAKAEAVVVGHPE

>Tdal_Td_comp159314

MLLTSFRFARTIISRQMVLRHYAKDVRFGPNVRALMMQGVGVLADAVEVTLGPKGRSVIIEEPWGPPKITKDGVTVAKSIALQDKFQNIGVMLVQDVATNTNEKAGDGTTTATVLARAIANEGFEKISKGANPIEIRRGVLLAINSAVDSLKAMSRPIKTNVEIEQVATISANGDHSIGVLISKAMQKVGRDGAITIKNGTSLSDELEFIEGLKFDKGYLSPFFINSSNGTKVEFQNALVLFSAKKIRAMSSIVPALELANQQKKPLLIIATDIDGDALSTLVLNRLQLGLPIAAVKAPLFGDATKELLIDMAVASGGAVCGDEEHFFKIENVMINDLGKVGEVIVTKNNTLLMNSGGNKAEIQRRIDSIKGQLSDTKSTYEKEKLQERIANLSSGVALLRVGGSSEVEVNEKKDRVTDALNATRAAIEEGIVPGGGTALLRCIPTLQALRGQNQDQNMGIEIICAALRKPCMTIAKNAGVDGATVVARVEMGAGDFGYDALKNEYGNLVDAGIIDPTKVVRTALMDAAGVASLLTTVEAVVTEFP

>Tdal_comp147828

MIRNSVFALRLLISRQVTLRQYAKDVKFGPEVRALMLQGVDVLADAVAVTMGPKGRNVIIEQSWGSPKITKDGVTVAKSIELQDKFQNIGAKLVQDVANNTNEEVGDGTTTAAVLARAIAKEGFEKISKGANPVEIRRGVMLAVDAVKDHLRLLSRPVRNKDEIAQVATISANGDKTIGNLISEAMKKVGRDGVITVKDGKTLTDELEVITGMKFDQGFKSPFFINSTKGAKVEFNDAYVLFSEKKITTVKSLIPVLELTHRLRKPLVIIAEDIDGEALSTLVVNRFKIGLQVAAVKAPGFGDKKKAMLTDMAIASGGIVFGDDTNVTKIEDVLINDLGKVGEVIITKNDTLLLKGKGSQDEIQRRIDQIKDQINDTTSYYDREKLKERLARLASGVALLRVGGSSEIEVNEKKDRVNDALNATRAAVEEGIVAGGGTALLRCIPKLENLQGVNADQSIGIDIVRRALRMPCMTIARNAGVDGAMVVAKVEQGADDFGYDALKGEYGFLIAKGIIDPTKVVRTAITDASGVASLLTTAEAVITDFPKDE

>Tdal_comp124036

MFRLPLSCSRLLISRQLGVRQFAKDVRFGPEVRALMLQGVDVLADAVAVTMGPKGRNVIIEQSWGSPKITKDGVTVAKSIELKDKFQNIGAKLVQDVANNTNEEAGDGTTTATVLARAIAKEGFEKISKGANPVEIRRGVMLAVDTVKENLKAMSRPVSTPEEIAQVATISANGDKAVGTLISEAMKKVGRDGVITVKDGKTLIDELEVIEGMKFDRGYISPYFINSTKGAKVEFQDALVLLSEKKISSVQSIIPALELANQQRKPLVIIAEDIDGEALSTLVVNRLKIGLQVAAVKAPGFGDNRKSTLMDMAIASGGIVFGDEANLTKLEDVLINDLGKVGEVVITKDDTLLLKGKGSKDEVQRRIDQIKDQISETTSEYEKEKLQERLARLASGVALLRVGGSSEVEVNEKKDRVNDALNATRAAVEEGIVPGGGTALLRCIPNLDNLKGQNDDQNMGVEIVRRALRMPCMTIAKNAGVDGAMVVAKVEIGKGDFGYDALKGEYGNLIEKGIIDPTKVVRTAITDASGVASLLTTAEAVVTEVPKED

>Aaeg_AAEL011584

MFRLPTVLRCTAARQVAAGYRGYAKDVRFGPEVRALMLQGVDVLADAVAVTMGPKGRNVILEQSWGSPKITKDGVTVAKGIELKCKFQNIGAKLVQDVANNTNEEAGDGTTTATVLARAIAKEGFEKISKGANPVEIRRGVMLAVDAVKDHLKTMSRAVTSPEEIAQVATISANGDRAIGDLISEAMKRVGKDGVITVKDGKTLHDELEIIEGMKFDRGYISPYFINSSKGAKVEFQDALVLFSEKKISSVQSIIPALELANSARKPLVIIAEDVDGEALSTLVVNRLKIGLQVAAVKAPGFGDNRKSTLSDMAISTGGIVFGDDANLVKLEDVQMSDLGQVGEITITKDDCMMLKGKGDSKHVEARVEQIRDQIAETTSEYEKEKLQERLARLSSGVAVLKIGGSSEVEVNEKKDRVNDALCATRAAVEEGIVPGGGTALLRCIKTLENLKGSNEDQKAGIDIVRRALHQPCTQIAKNAGVDGSVVVAKVLDQQDDFGYDALNGEYVNMIEKGIIDPTKVVRTALTDASGVASLLSTAECVVTEEPKPEGAGGMPGMGGM

>Gmor_GMOY002421PA

MLRLSATLARSGIRHQLAVRGYAKDVKFGPEVRAMMLQGVDVLADAVAVTMGPKGRNVIIEQSWGSPKITKDGVTVAKSIELKDKFQNIGAKLVQDVANNTNEEAGDGTTTATVLARAIAKEGFEKISKGANPVEIRRGVMMAVDTVKDHLKTMSRPVSTPEEIAQVATISANGDHNIGNLISEAMKKVGRDGVITVKDGKTLSDELEVIEGMKFDRGYISPYFINSSKGAKVEFQDALVLFSEKKISSVQSIIPALELANQQRKPLVIVAEDIDGEALSTLVVNRLKIGLQVAAVKAPGFGDNRKSTLTDMAIATGGIVFGDDANLVKLEDVNINDLGKIGEVVITKDDTLLLKGKGKKEDVQRRVEQIKDQIAETTSDYEKEKLQERLARLAAGVALLRVGGSSEVEVNEKKDRVNDALNATRAAVEEGIVPGGGTALLRCITKLDTLKGQNEDQNMGIEIVRRALRMPCMTIAKNAGVDGAMVVAKIETKEGDYGYDALKGEYCNLIEKGIIDPTKVVRTAITDASGVASLLTTAEAVVTELPKEESGPA

>Gmor_GMOY006640PA

MFRLPVTLACSRITSQLAVRGYAKDVKMGPEVRAMMLQGVDVLADAVAITMGPKGRNVIIEQPWGSPKITKDGVTVAKSIDLKDKYQNIGAKLVQDVANNTNEEAGDGTTTATVLARAIAKEGFEKIFKGANALEIRRGVMMAVDIVKDHLKSMSRPVNTPEEIAQVATISANGDHNIGNLISEAMKKVGRDGVITVKDGKTLTDELEVIEGMKFDRGYISPYFINSSKGAKVEFQDAFVLFSGKKISSVQSIIPSLELANQHRKPLVIIAEDIDGEALSTLVVNRLKIGLQVAAVKAPGFGDKRIAALTDMAIATGGIVFGDDANIVKLEDVNINDLGKIGEVVITKDDTLLLKGKGKKEDVQRRIEQIKDQIAETTSDFEKEKLQERLARLAAGVALLRVGGSSEVEVNEKKDRVRDALNATRAAIEEGIVPGGGTALLRCIAKLEALKVNNHDQNMGIEIVRRALRMPCMTIAQNAGVDGAMVVATIESKEGEYGYDALKGEYCNLIEKGIIDPTKVVRTALVDASGVASLLTTAEAVVTEIPKEGGDPA

>Ppap_PPATMP003142PA

MLKGVDVLADAVAVTMGPKGRNVILEQSYGSPKITKDGVTVAKGIELKDKFQNIGARLVQDVANNTNEEAGDGTTTATVLARAIAKEGFEKCSKGANPVEIRRGVMLAVDSVKASLKTMSKNVTTPEEXXXMKKVGREGVITVKDGKTLHDELEVIEGMKFDRGYISPYFINSSKGAKVEFQDALVLLSEKKISSIQSIIPALELANSQRKPLVIIAEDVDGEALSTLVVNRLKIGLQVAAVKAPGFGDNRKSTLQDMAIATGGIVFGDDANLVKIEDVQVADFGQVGEIVITKDDTLILKGKGKKDDIDRRSEQLRDQIQETTSEYEKEKLQERLARLASGVAVLKVGGSSEVEVNEKKDRVNDALNATRAAVEEGIVPGGGTALLRCIPGLNQLKGANSDQ

>Mdom_MDOA007155PA

MFRLPVALARSNIARQLAVRGYAKDVKFGPEVRALMLQGVDVLADAVAVTMGPKGRNVIIEQSWGSPKITKDGVTVAKSIELKDKFQNIGAKLVQDVANNTNEEAGDGTTTATVLARAIAKEGFEKISKGANPVEIRRGVMLAVETVKDNLKAMSRPVSTPEEIAQVATISANGDKAVGNLISEAMKKVGRDGVITVKDGKTLTDELEVIEGMKFDRGYISPYFINSSKGAKVEFQDALLLLSEKKISSVQSIIPALELANQQRKPLVIIAEDIDGEALSTLVVNRLKIGLQVAAVKAPGFGDNRKSTLTDMAIASGGIVFGDDANLVKLEDVTMADLGKVGEVVITKDDTLLLKGKGKKEDVDRRVDQIKDQIAETTSEYEKEKLQERLARLASGVALLRVGGSSEVEVNEKKDRVHDALNATRAAVEEGIVPGGGTALLRCIAKLEGLKGQNEDQNMGIQIVRSALRMPCMTIAKNAGVDAAMVVAKVESQEGDFGYDALKGEYGNLIEKGIIDPTKVVRTAITDASGVASLLTTAEAVVTELPKEDAPA

>Agam_AGAP004002

MFRLPTVLRVAAARQVAASRGYAKDVRFGPEVRALMLQGVDVLADAVAVTMGPKGRNVILEQSWGSPKITKDGVTVAKGIELKCKFQNIGAKLVQDVANNTNEEAGDGTTTATVLARAIAKEGFEKISKGANPVEIRRGVMLAVDTVKEHLKTLSRKVNTPEEIAQVATISANGDRAIGDLISEAMKRVGKEGVITVKDGKTLNDELEVIEGMKFDRGYISPYFINSSKGAKVEFQDALVLFSEKKISTVQSIIPALEMANQQRKPLVIIAEDVDGEALSTLVVNRLKIGLQVAAVKAPGFGDNRKSTLSDMAISTGGIVFGDDANLVKLEDVQLSDLGQVGEITITKDDCLLLKGRGKQEDVNRRADQIRDQIAETTSEYEKEKLQERLARLSSGVAVLKVGGSSEVEVNEKKDRVNDALCATRAAVEEGIVPGGGTALIRCAPALANLKGANEDQNTGIEIVRRALRMPCTQIAKNAGVDGSVVVAKVEELKGDFGYDALNNEYVNMIEKGIIDPTKVVRTALSDASGVASLLTTAEAVVTEIPKEEPAGP

>Cqui_CPIJ008889

MFRLPAVMRCAAARQVAYRGYAKDVRFGPEVRALMLQGVDVLADAVAVTMGPKGRNVILEQSWGSPKITKDGVTVAKGIELKCKFQNIGAKLVQDVANNTNEEAGDGTTTATVLARAIAKEGFEKISKGANPVEIRRGVMLAVDAVKDHLKSLSRTVTSPEEIAQVATISANGDRAIGDLISEAMKRVGKDGVITVKDGKTLIDELEVIEGMKFDRGYISPYFINSSKGAKVEFQDALVLFSEKKISTVQSIIPALELANAQRKPLVIIAEDIDGEALSTLVVNRLKIGLQVAAVKAPGFGDNRKSTLSDMAISTGGIVFGDDANLVKLEDVQLSDLGQVGEISITKDDCMLLKGKGNADHVSARAQQIRDQIEETTSEYEKEKLQERLARLSSGVAVLKVGGSSEVEVNEKKDRVNDALCATRAAVEEGIVPGGGTALLRCIKALDNLSGANDDQKAGIDIVRRALHQPCTQIAKNAGVDGSVVVAKVLDLQGDFGYDALNSEYVNMIEKGIIDPTKVVRTALTDASGVASLLSTAECVVTEEPKAEGA

>Tcas_XP_971630

MYRLPSTMRSVALSKANRLSQIQRWYAKDVRFGPEVRALMLQGVDVLADAVAVTMGPKGRNVIIEQSWGSPKITKDGVTVAKGVELKDKFQNIGARLVQDVANNTNEEAGDGTTTATVLARSIAKEGFENLGKGANPVEIRKGIMLAVEKITETLKTLSKPVTTPEEICQVATISANGDQSVGNLIADAMKKVGKEGVITVKDGKTLHDELEVIEGFKFDRGYISPYFVNTSKGAKVEYQDALILLSEKKISSVQSIVPALELANMQKKPLIIVAEDIDGEALTTLVVNRLRIGLQVAAVKAPGFGDNRKATLQDMAIATGGIVFGDEANIVKLEDVQLSDLGQVGEIVITKDDTLILKGKGKKDDISKRAEQIKDQIENTTSEYEKEKLQERLARLASGVALLKVGGSSEVEVNEKKDRVTDALNATRAAVEEGIVPGGGTALLRCSGSLDGLKPGNNDQAIGIEIVKRALKVPCMTIAKNAGVDGATVVAKIEQQQGDYGYDALNNEYVNMFERGIIDPTKVVRTALIDASGVASLLTTAEAVITEIPKEEPPIPSGGMGGMGGMGGMGGMM

>Amel_XP_392899.2

MHRLPTILRSAALRQLQTRSYAKDVRFGAEVRALMLQGVDILADAVAVTMGPKGRNVILEQSWGSPKITKDGVTVAKGVELKDKFQNIGAKLVQDVANNTNEEAGDGTTTATVLARAIAKEGFEKISKGANPVEIRRGVMLAVDKVKDELKALSKPVTTPEEIAQVATISANGDKAIGNLISDAMKKVGKEGVITVKDGKTLHDELEVIEGMKFDRGYISPYFINSSKGAKVEFQDALLLFSEKKISSVQSIIPALELANSQRKPLVIIAEDIDGEALSTLVVNRLKIGLQVAAVKAPGFGDNRKATLQDMAILTGGIVFGDDANLVKLENVQLCDLGEVGEVVITKDDTLFLKGKGKKSDIDHRADVIRDQIANTTSDYEKEKLQERLARLASGVAVLRVGGSSEVEVNEKKDRVHDALNATRAAVEEGIVPGGGTALLRCIPALRNLKASNNDQETGIKIVANALRMPCLQIAQNAGVDASLVVAKVSDGNLGYDALNDEYVDMIEKGIIDPTKVVRTALTDAAGVASLLTTAEAVVAELPKEEPQMPMGGGGMGGMGGMGGMGGMGM

>Dwil_XP_002065107

MLTRFRHNKFLRSFANDVRFGPEARELLMRGVNILADAVATTLGPKGRNVLIEQLLRSPKITNDGITVANNVHLEDRRINMGAQLIRLATNNTNNNVGDGTTTTTILARSMASQGMELLQNGSLNVQELREGIIQGSKAVCKELRSMSQAIERIDQVRSVANNALNNDQNLAQLIGKGVLELGEGGVFLLKESMSAKDELVIQEGISLAEGFASPLFAHKSENGRLEFSQMLVVLTLAEIEKLSDILPLLELARNANQPLLLIAKEFSDEVLTALILNHLENRLQVCAVRAPYFGAEQKEQMEDISLALGLQLFENLSWLRNIKAEDLGTVREVIIDSKATHLIQQHTEKAEQVENRIQHIKSLIKEAATDEEIERLNERLGRLMGHVAIVHVGGDSELEIDEKKDRLNDALHSVKVAISDGILPGGGTAYLRCINALKYLHLKDIPEHRMGVDIVRNALKLPCYIIAQNSGANPDEVIHKILNEQGNFGYDAANGEFCDMIDRGIVDPTRVMCSAITDAAGIASLLTTTDVLITELKKKPNIPKNQVTKDLASLIGM

>Dyak_XP_002088604

MLSRLGRSGGTGVRSYAKDIRFGAEARCLLMQGVNVLANAVATTLGPKGRNVLIEQLLISPRITKDGVTVAHNVQLRNRRQDMGVQLLRQATNNTNNKVGDGTTTATILARGIACQGMHVLRQSQVNVQLLREGILEGSRTVCKALGEMSQSVDTIGQVEAVAKVALNGDERLAELIGDVILELGEDGVILLKESHSPFDEVKIQEGVTLASGYCSPFFAKKGGALELENCLLLLTLAKIDQVEQILPALELARLKERPLLIVAKNFGSDLLKILVLNHLQGLVQVCAVKAPSFGEEQCEEMEDLAIATGGHLLEDASRLADLSEEDLGEVLEAVVDAKETHLLQPINVNEEQVQCRIQHIRELIEEAFTDEELDRLKTRLGRLQGHLATIFVGGTSDLEVSERKDRFNDALHAVRVAISEGVVPGGGTAYLRCIPALDELPPTEVMEHQVGREIVKDALRLPCYTIARNAGVDPNVVLGRVLEGSGSYGYDAAAGEFEDLVLRGIVDPTKVMQSAMTAAAGIASLLATTEVLITNQPTKVKIPKNQVTRDLAKLVGM

>Mdes_gbAEGA01018086

MYRLPNMLRSSVARQVAVRAYAKDVRFGAEVRALMLQGVDVLADAVAVTMGPKXXXGRNVIIEQSWGSPKITKDGVTVAKSIELKDKFQNIGAKLVQDVANNTNEEAGDGTTTATVGRNVILEQSWGSPKITKDGVTVAKGIELKDKFQNIGAKLVQDVANNTNEEAGDGTTTATVLARAIAKEGFEKISKGANPIEIRRGVIMAVDAVKENLKKLSRQVTSPEEIAQVATISANGDRAIGDLISEAMKRVGKDGVITVKDGKTLHDELEVIEGMKFDRGYISPYFINSSKGAKCEFQDALVLISEKKISSIQSIIPALELSNAQRKPLVIIAEDVDSEALSTLVVNRLKIGLQVVAVKAPGFGDNRKSTLKDIAISTGAVVFGDEGNLVKLEDIKVSDLGQIGEVVVTKDDTLLLKGKGKQSDIERRVQQIRDQIEDTTSEYEKEKLQERLARLSSGVAVLKVGGSSEVEVNEKKDRVNDALCATRAAVEEGIVPGGGTALIRCIPILDNIKGKNEDQXXXNTGIGIVKRALRMPAHTIATNAGVDGAVVVAKIEGSAAEIGYDAMNDEYVNMVEKGIIDPTKVXVVRTALTDASGVASLLTTAECVVTEIPKEE

>Cnas_XP_031621812

MYRLPNVLRSAAARQLAVRSYAKDVRFGAEVRALMLQGVDVLADAVAVTMGPKGRNVILEQSWGSPKITKDGVTVAKGIELKDKFQNIGAKLVQDVANNTNEEAGDGTTTATVLARAIAKEGFEKISKGANPIEIRRGVILAVDAVKENLKKLSKPVTSPEEIAQVATISANGDKAIGDLIGEAMKKVGKDGVITVKDGKTLHDELEVIEGMKFDRGYISPYFINSSKGAKCEFQDALVLVSEKKISSIQSIIPALELANAQRKPLVIIAEDVDSEALSTLVVNRLKIGLQVVAVKAPGFGDNRKSTLQDIAISTGALVFGDDANLIKLEDVKVSDLGQIGEVVITKDDTLLLRGKGQKTDIDRRVEQIRDQIEETTSEYEKEKLQERLARLSSGVAVLKVGGSSEVEVNEKKDRVNDALCATRAAVEEGIVPGGGTALIRSIPVLDGLKGKNEDQNTGIAIVKRALRMPAMTIASNAGVDGAVVVAKIETSPPEIGYDALNNEYVNMVEKGIIDPTKVVRTALTDASGVASLLTTAECVVTEIPKEEPAGGAPGMGGMGGMGGMGGMGGMGGF

>Smos_VUAH01000001

MYRLPNVLRSAAARQLITRSYAKDVRFGSEVRALMLQGVDVLADAVAVTMGPKVRTALTDASGVASLLTTAEAVVVEIPKEEXKGRNVILEQSWGSPKITKDGVTVAKGIELKDKFQNIGAKLVQDVANNTNEEAGDGTTTATVLARAIAKEGFEKISKGANPVEIRRGVILAVDAVKDNLKKMSKPVTSPEEIAQVATISANGDSAIGQLISDAMKKVGKDGVITVKDGKTLHDELEVIEGMKFDRGYISPYFINSSKGAKCEFQDALVLISEKKISSIQSIIPALELANAQRKPLLIIAEDVDSEALSTLVVNRLKIGLQVVAVKAPGFGDNRKSTLQDIAISTGALVFGDDSNLIKLEDVKVSDLGQIGEVVVTKDDTLLLRGKGNKTDIDRRSQQIRDQIEETTSEYEKEKLQERLARLSSGVAMLKVGGSSEVEVNEKKDRVNDALCATRAAVEEGIVPGGGTALIRSIPVLDNLKGKNEDQXNIGIGIVKRALRMPALTIATNAGVDGSVVVAKIETSAPEIGYDALNDEYVNMVEKGIIDPTKV

>Pcoq_Hsp60

MLRLATILNRSPIAKSMVVRSFAKDVKFGPEVRALMLQGVDILADAVAVTMGPKGRNVILEQSWGAPKITKDGVTVAKAIELKDKFQNIGAKLVQDVANNTNEEAGDGTTTATVLARAIAKEGFEKISKGANPVEIRRGVMIAVDTVKDHLKTMSRPVSTPEEIAQVATISANGDRNIGNLISEAMKKVGRDGVITVKDGKTLNDELEVIEGMKFDRGYISPYFINSSKGAKVEFQDALVLFCEKKISSVQSIIPALELANSQRKPLVIIAEDIDGEALSTLVVNRLKIGLQVAAVKAPGFGDNRKATMQDMAISTGGIVFGDEANLVKLEDVQLSDLGKVGEVVITKDDTLLLKGKGKKEDIERRAEQIRDQITETTSDYEKEKFQERLARIASGVALLRVGGSSEIEVNEKKDRVNDALNATRAAVEEGIVPGGGTALLRCIPVLDKLKAANDDQ
