# Supplementary material for: Genomic signatures of globally enhanced gene duplicate accumulation in the megadiverse higher Diptera fueling intralocus sexual conflict resolution
Source: PeerJ. 2020 Oct 12;8:e10012. doi: 10.7717/peerj.10012 (PMC7560327; doi:10.7717/peerj.10012)
Supplement: Supplemental Information 9 [file peerj-08-10012-s009.zip › Men protein sequences 2020.docx]

>Dmel_Men-b

MFSRPSLCGTVGKLCRCGTSATGKTAVAAATVPTARHYHEVVGDIICPSQVRGIDHIRDPRLNKGLAFTLEERQTLGIHGLQPARFKTQEEQLQLCKIAVNRYTEPLNKYLYLSDLYDRNERLFFRFLSENIEDLMPIVYTPTVGLACQRFGLIYRRPHGLFITYNDRGHIFDVMKNWPEPNVRAICVTDGERILGLGDLGACGMGIPVGKLALYTALAGIKPHQCLPIVVDVGTNNIDLLEDPLYVGLRQKRVVGREYDEFIDEFMEAVVQRYGQNTLIQFEDFGNHNAFRFLDKYRNTYCTFNDDIQGTASVAVAGLYASKRITGKSFKDYTFLFAGAGEAAIGIADLTVKAMVQDGVPIEEAYNRIYMVDIDGLLTKSRKVGNLDGHKIHYAKDINPMSDLAEIVSTIKPSVLIGASAAAGIFTPEILRTMADNNERPVVFALSNPTSKAECTAEDAYKHTDARVIFSSGSPFPPVQIGDKTFYPGQGNNAYIFPGVGLGVICTGTHHIPDEMFLIAAQELANFVEPSDIERGSLYPPLSSIRNVSMNIAVGVTKCAYDRGLASTYPEPQDKRKWLENQLYNFNYESSMPASWVWPRMPYIKTREESPLIAAIK

>Dmel_Menl-1

MSFFRLLNFGITRGSSISKTHQSNCECSPNLIKIRPSSSHDTIRVPIEVVFSDKCNKALAFTLEERQRLCIHGLMPACVRTYDEQMLAIESNFHSFESNVGRYRYLRALRQGYERLYFQFVSKNVHAVLPIIYTPTVGLACTVYGMLYRGMTGIHITKHDRGHMKQILSNWPMRRSVKAICVTDGQRILGLGDLGANGMGIAVGKMELYTALAGIPPSMLLPICLDVGTNNKSLHEDPLYIGLRDERLKGDEYVCFVDEFMEAVVSTFGDQTLIHFEDFATPNAFMFLNRYQHCYCHFNDDIQGTAAVGLGGLLGIQRITKKPLEEHVIVFAGAGSAAMGIAYLLKMELMSRGLSEADAAKNIYFYDQDGVLTTARKSIPDILCVFAKNMKETKSLETLVEQVKPSIIMGATSAPGLFTEKIIRTMAASHERPGIFAFSNPTIKSECTAEQAYKFSDGKAIYSAGSPFPPVEFNGKRLTPGQANNCFAFPALVLATMTVLATRMPDEIFLLAAHELAEFPTNEEMQSGRIYPLVKQANEVAYKIGVKVAKYLIENGYAKRNLEPEDVEEYIEKNSYKLTYGSSLAETWAYPKMKPHPSTAGHENKHQKKK

>Dmel_Menl-2

MEFRRLHPLLRLASRPLLSSSRRFSVYDDEMEQITRPDWHVVMNGKYNKGLAFTIKERQRLGIMGLMPCSVRSMDDQMNAALANFEARPTDIARFTYLSAVHHRHRRLYYRFIKENIEKSLPIVYTPTVGDVVATYGLNFQQAISLFISIHDKGHIRDLMHNWVDEGVKAICVTDGGRVLGLGDMGANAMGISLGKMILYTALGSIPPSTLMPVCLDVGTDNQALLQDPLYVGARIPRVKGPEYDELVDEFMESAVKCFGNNTFIHFEDFATPNALKFLEKYQYKYCCFNDDIQGTGATGLAAFINVERITGRKLEDTVFLFVGAGSAALGIANMLAMELEVRGIPAEEATKNIYLMDVNGILTPESPNPPEMGKIFIKSMEPMKDMMAVLKKLKPSVLVGATGVGGIFNEEVLKTMAKNHERPAVFPLSNPTANSECTAEQAFTHTEGRVLFGSGSPFPPVVINGKRYRPAQANNCLTFPGIALAAITAKARYLPNEVFSVVSHELARNTPQELLDEGTLFPPIKDAHNVAFNVGVAMTQYLIDNDLSNVYPKPDDICEFVKRSLYKFDYRNSLPTTWDYPVEPPTPKPKTKPKTKPKE

>Dmel_CG7848

MQKSPRMSRLSAEKKKKKDDEKKDNAEEELQKNKELKCVSNYDKTEILDYMPRYMDTFWKMDTRLSGSKVDGLWMLNQSNYNKGLAFTLNERRVLSIHGLLPVAVRTIDEQAEICSNLLESFTNNVQQYIYLTYLSRRNRRLFYYLLLSNPDRFVPMTDASGSIDLLMVHRMIHSMGQGLYICIKDLGHVSQILSNWPFRCVRCLLVSNGASVLSVGDLGVDEMPILFSNLHQNVVYGGIHPAYCLAVMLDVGTNNEELLNDPMYTGLRERRCSEKLYDQLFEEFTLSVMQQYGPRALILCKDFEAQKAKKQLELYRERQCIVDVDFQCFAAVALAGVIVCNRLKRVFFSSNVFLFYGAEAINIGMARLCMVLLKREGLIEMKAREKIWFFDANGLVVLGRKDIPEELLEFANQRDPILDLVEAIQELKPNILVGGSSLPNTFTPDVLRAMEKSADQPVIFALSRPLEQTECSAEDAFSYTKGHCIFISGSKLPPLKYANKWYQPGHCTSSYLVAGISCGVMLAGFTNIPDEAFCVAAERLASLVWPCDLEKRNVYPPMRKIQCISLQIAEAIFSYAFGRGLATLYPQPENPMEYIKNSMYNPEYRMNIVDVYCMQNRSIATTESRKYYTLDI

>Dmel_Men

MYSILRRCSGIRKTFGPTPVYPTANNSQSPSSYSRGKERELGCYTKRNSNSNNNNSHERESQSCCSSRVCKNHTTTTTTTLEYELSNFAKLTTRITTQSAAEVDTSPHTDTETHRDRDSNPGNIALATDLELPKGLPLSLSSRHHWNQLQSSLHALHHQQQQQQQQLRSYSSTSETNLEDKMSKPDSKLDKYAQRDRLGLWGTGDNEVVGSLSGFTRLLDKRYSKGLAFTHEERQQLGIHGMLPYVVREPSEQVEHCRALLARLDQDLDKYMYLISLSERNERLFYNVLSSDIAYMMPLVYTPTVGLACQRYSLIHQNAKGMFISIKDKGHIYDVLKNWPETDVRAIVVTDGERILGLGDLGANGMGIPVGKLSLYTALAGIKPSQCLPITLDVGTNTESILEDPLYIGLRERRATGDLYDEFIDEFMHACVRRFGQNCLIQFEDFANANAFRLLSKYRDSFCTFNDDIQGTASVAVAGLLASLKIKKTQLKDNTLLFLGAGEAALGIANLCLMAMKVEGLTEEEAKARIWMVDSRGVITRDRPKGGLTEHKLHFAQLHE

PIDTLAEAVRKVRPNVLIGAAAQGGAFNQEILELMADINETPIIFALSNPTSKAECTAEEAYTYTKGRCIFASGSPFAPVTYNNKKFYPGQGNNSYIFPGVALGVLCAGMLNIPEQVFLVAAERLAELVSKDDLAKGSLYPPLSSIVSCSMAIAERIVEYAYKNGLATVRPEPVNKLAFIKAQMYDLDYPRSVPATYKM

>Dvir_XP_002056217

MGNSSSICADRNAIRNFDENGTPIYPTANNSHSPSNGQITPYHHQQQQQQQQLNEHGYYKTTIPVGVGTGSAVNSSSSKR

CSHCQTADLCSRALVQESSGNNNNNNNKINAAQPTRIHNHTTHTCESTSSCDSDCAAAATAKWRSSRVCKNHTTTTITTL

EYELSNFAKLTTQISTQRDVDEATARAVEAEALPSPPPPPPILSSSHLQQLQQSLHELHQQQTQQLIGKPSNNLQQIRRY

SCDMSKTNDLHRDRLGLWGTGDNEIVGNVSGLERLQNKRYNKGLAFTHEERQVLGIHGLLPYVVRSDDQQVEHCRILLNR

LENDLDKYVYLIGLSERNERLFYKLLSSDIAHMMPLVYTPTVGLACEKYSLVFHKPKGMFISIKDKGHVYDVLKNWPEMD

VRAIVVTDGERILGLGDLGANGMGIPVGKLSLYTALAGIKPHQCLPITLDVGTNTQSILDDPLYVGLRQARATGDLYDEF

VDEFMRACVRRFGQNCLIQFEDFGNANAFRLLSLYRDKYCTFNDDIQGTASVAVAGLLASLKIKKTLLKDNVLLFLGAGE

AALGIATLCTMAMKEEGLSDEEAKARIWMVDSRGVIVRDRPKGGLTEHKLHFAQVSTPIDTLMEAVIKVRPNVLIGASAQ

GGAFTKEILEKMAEINETPIIFALSNPTSKAECTAQDAYTYTNGRCIFASGSPFAPVTYNGRKFYPGQGNNSYIFPGVAL

GVLCAGMLTIPEEVFLISAQCLAELVNKEDLNKGSLYPPLNSIVQCSLAIAERIVCYAYQNGLATVQPEPESKLAFIKAQ

MYDIDYPRALPATYPL

>Dvir_XP_002050845

MRAKKQQPVHKCIDEDAKLLTLEGMPRELDTFWRTDSQLPRSQLSGHRMYRLSSLNKSSAFSHRERQLLSIHGFMPAAVFTVKQQLEACTQHFATLTSNFQKYIFLTELEGFNRKLFFNLLISDPETFMSVFKSSEFYFS

VKNFSILYTSTRGMYLTIKDRGHIYDVLRNWPRRHDVRYLVVTNGDSVLSMGDYGVNAAPVVFFKLYQNVAYGGVNPDSCLPVMLDVGTNNEELLRDPMYLGLPQRRVTGAEYEAFFEEFTVAVLRLFGPRAIIQTKNFGALDSIKQLERYRKRQCFMDVSLQALGACGLAGLLAANKITEGTFKANKLLFYGNGTFNIGMARMCLAYLKRLRLDESAARERIWFCDAHGLIVHGRCDHKVPTELLEFKHRHEPVGSLLDAINLLKPNVLVGCSSEPNVFTKDIIRAMEQSAEQPIIYAMSTPLELAECCADDAFVYTKGHCIFISAAQLPSLKYANKVYQPGYCNVQYM

LPGLTLGVMLSGMTSVPDETFLVAADRLANLVWPNDMAKRDVFPPMRKLKCINLQITEAVFAYAYRRNLA

TLWPEPTNPKHYIESMLYDPEYVEVCQPIYCITDQQIGTTESIQYYKQKI

>Dvir_XP_002050844

MAYSQLLVGIKRNFGVLLCAKAQKCKINECPRLPRRYRHANVIKAEYRKVTDPHYNKGMGYTHDERQKLNVIGLFPSAYRSEAEQISAVNANFHAQQSDLARYLYLRTLRSRQERLYYRFVIEKIEEVMPIIYTPTVGAV

CQAFSLIYHSTMGLYVSKYDKGYMTDVLSNWPNTDIRAVCVTDGERILGLGDLGAGGMAISVGKLDLYTALAKVPPQYLIPVVLDVGTNNQQLLSDPLYIGVREKRCKGKEYEDLVQEFMDAVVKVWGYQTLIHFEDFSTPNAFKFINMYQDKYCNINDDIQGTASCGLAGFLAVEHITKKPLKDHTVLFVGAGSAALGISKLLVKELIS

RKLSEEEAVKNIYITDVEGLITKDRQKFDIPDLKLFAKDRPPVKDLEQLVKEIKPSILLGATGQGGIFTE

NILRIMGSENEHPAIFACSNPTNKAECTAEQAYNFTEGRALFASGSPFPPVVINGKRLIPTQANNAFAFP

GIALGVMCTHPRTISDDVFLVAAHEIAKYCREFYPNDAALYPPIKEAANVAFSVGVAVAKFLIDERKANV

YPIPTNVCEFVQSYQYYTERHATLPLTWEYPNMSAPLPKGDDKDCKN

>Dvir_XP_002054324

MFSRPSLCGNLGKLCRSGTAAVTSTPTTTPPALSALALCDARGYHEVVGDIICPSQVRGIDHIRDPRLNKGLAFTLEERQ

VLGIHGLQPARFKTQEEQLQLCKIAVNRYTEPLNKYLYLSDLHDRNERLFFRFLSENIEDLMPIVYTPTVGLACQRFGLI

YRRPHGLFVTFNDRGHIFDVMKNWPEPNVRAICVTDGERILGLGDLGACGMGIPVGKLALYTALAGIKPHQCLPIVVDVG

TNNIDLLEDPLYVGLRQKRVVGREYDDFIDEFMEAVVKRYGQNTLIQFEDFGNHNAFRFLDKYRNTYCTFNDDIQGTAAV

AVAGLYASKRITGKSFKEYTFLFAGAGEAAIGIADLVVKGMVAEGVPIEEAYSRIFMVDIDGLLTTSRNVGNLDGHKVNY

AKDIEPMQDLEQIVSTVKPSVLIGASACAGLFTPKILRTMADNNERPIIFALSNPTSKAECTAEEAYHNTDARVIFSSGS

PFPPVVVGNKTYYPGQGNNAYIFPGVGLGVICTGTHHIPDDMFLIAAQELANFVEPADIERGSLYPPLSSIRDVSMNIAI

GVTKCAYDKGLASTYPEPQDTRKWLEDQLYNFNYECSMPVTWTWPRMPYIKTREESPLIAAIK

>Mdom_MDOA008701

MDHCNGNTKSLDFAPRDRQGLWSANGDNEVPGSVSGLQRLQQKKFNKGLAFTLEERQLLGIHGLLPAVVKSEEQQIQHCLLLLDRLESDLDKYIYLNSLAERNERLFYKILATDISKMMPLVYTPTVGLACQKFSLIFQYPKGLYITIKDRGHVYEVLKNWPEFDVRAIVVTDGERILGLGDLGANGMGIPVGKLSLYTALAGIKPSQCLPITLDVGTNTESLINDPLYIGLRHKRIVGKQYDEFIDEFMHAAVRRFGQNCLIQFEDFANANAFRFLAKYRNDFCTFNDDIQGTASVAVAGLLASLKIKNTKLKENKILFLGAGEAALGIASLCLMALIKEGLTEAEAKKLIWMVDSKGLIVQNRPAGGITEHKQNFAQAHASIDSLEEAVKRIKPTVLIGAAAIGGAFTKSILEMMADYNENPIIFALSNPTSKAECTAEQAYQYTKGKCIFAAGSPFKPVLYDSKTYYPGQGNNSYIFPGVALGVLCTGMLTIPEEVFLMSAECLANLCKPEDLAKGSLYPPLNRITGCSVEIAAYIMEYAYKNGLATVRPEPNDKRAFIRSQMYDLSYPPAVPEVYMWEQKL

>Mdom_MDOA007993

MISKVRQPFLFSIQISRYFSRSPVYPSAVNSYSPSHGGQRTPNNEHHYYKTTIPINEQKQQQQCKCNSNGTIQTATADGKDTPAAAAASIASMRNACHGLHDVTTNVVQSGVPSSMSTLATATPPVCSCMGMRSGQANENHTTATTNTANTTTTTTTTIEYELTNGIAKLSKISTSRVAAEQPSNANAASANVPKAESQSRLNCDTVQSQEETTSKVPLLSQADLDDLMRNVSAINAVNRTLRHTFSNQQGHLLHHQQQQQQHQLSALSNLQQRSNIHMDFSSMNGSSNNSPLKKVPRDRLGLWGAGGDNDVPGNVSGLQRLQQKKYNKGLAFSLEERQVLGIQGLLPVRVKTEEEQVEHSLLLLDRLENDLDKYMYLNTLAERNERLFYKVLSSDVAKMMPLVYTPTVGLACQKFSLIFQYPKGMYININDKGHVYEVLRNWPETDIRAIVVTDGERILGLGDLGANGMGIPVGKLSLYTALAGIKPSQCLPITLDVGTNTESILNDPLYIGLRQKRVTGQEYDEFIDEFMNAVVRRFGQNCLIQFEDFANANAFRLLKKYRDGFCTFNDDIQGTASVAVAGLLASLKIKNTQLKENKILFFGAGEAALGIANLCLMALMKEGLSEAEAKERIWMVDSKGLIVKDRPAGGLTEHKLHFAQDHVPINSLAEAVETLQPSVLIGAAAIGGAFTKEILEKMAEYNDVPIIFALSNPTSKAECTAEEAYKYTNGKCIFASGSPFDPVEYNGKTFYPGQGNNSYIFPGVALGVLCAGMLTIPEEVFLMSAERLADLCEPEDLERGSLYPPLKKITECSVEIAAYIMEYAYKNGLATVRPEPDDKREFIKSQMYDLHYSSAIPEVYSWNHKL

>Mdom_MDOA007103

MFSRPLLYGNFSKICASAAARQPHQSVTQPTTASTAVANNNTQIREYHEVVGDILCPSQVMGIDHIRDPRLNKGLAFTLEERQVLGIHGLQPARFKTQEEQLELCKIAVNRYSESLNKYLYLSDLQDRNERLFFRFLSENIEEMMPIIYTPTVGLACQRFGLIYRRPRGLFVTINDRGHVFDVIKNWPEPDVRAIVVTDGERILGLGDLGASGMGIPVGKLALYTALAGIKPHQCLPILIDVGTNNIDLLEDPLYVGLRQKRVVGREYDDFIDEFMQAVVKRYGQNTLIQFEDFGNHNAFRFLDKYRNTYCTFNDDIQGTASVAVAGLYASKRITGRSFAEYTFLFAGAGEAAIGIADLVVKAMVAEGVSLEEARSKIWMVDIDGLLTTTRKDGNLEGHKFNYAKEVEPMKNLEDIVDKIKPNVLIGASAAAGIFTPKILQTMAANNERPVVFALSNPTSKAECTAEQAYQNTEGRVIFSSGSPFPPVTIGDKTFYPGQGNNAYIFPGVALGVIATGTHHIPDDMFLIASQELANYVSDADLERGSLYPPLSSIREISMRIAMGVTKCAYDKGLASTYPEPQDKRKWLEEQLYNFNYESSLPVTWVWPRMPYIKTRDESPLLAAIK

>Ccap_XP_004523446

MQLKALSPLIIKSRLFSVGMCYGARSPLYPTVANSYNPSNGQVTPYDQYRQQQQPQQQLQTQMYKTTIPV

NEQCHQKQQQQQQQCQVTATANSCHCSSTSGAFAQSAVIGTQLSNNNTNTNDDAMNVKQLAIGIPTSGQY

CCQATTNNNGNRTTFASTQAHCAHRNSSQASNNHHTTTLTTTTTTVEYELSNGIAKLSKISTSRVAAEQS

SLPSQLSQTQQTATPSKEEKEVDDLTEITTTSSNQNLKENQKENETNLLPPLLSSKALLDLNKSIEALNS

YNNFNRNIQQQQLRNYSTTMSQCLKQKTTAVLADDAEVPGKVSGLERLHYKKYNKGLAFTVEERQLLGIQ

GLLPAVVKTEEEQLKHARILLDRLENDLDKYMYLSSLAERNERLFYKVLASDISNMMPLVYTPTVGLACQ

KFSLIFQNPKGLYITIRDKGHVYDVLKNWPETDIRAIVVTDGERILGLGDLGANGMGIPVGKLSLYTALA

GVKPHQCLPITLDVGTNTQSILDDPLYIGLRHKRITGQEYDAFIDEFMQAVVRRFGRNCLIQFEDFGNSN

AFRLLDKYRDNYCTFNDDIQGTASVAVAGILASLRLTETTLMENKILFFGAGEAALGIANLCKIAMIRLG

LTDKEALDRIWLVDSRGLIVKNRPSGGLSEHKLHFAHQHEPVDTLLDAVKTVKPTMLIGAAAVGGAFTPE

ILQLMAELNERPVIFALSNPTSKAECTAEQAYQNTDGRVIFSSGSPFAPVTYKGKTFYPGQGNNSYIFPG

VGLAVIAAGIKTIPEEIFLMAAQELSLMSQEEDLAKGSLYPPLEKITECSIGIAVAIVKYAYDEGLATVF

PKPENICEFIKSQMYNVNYSPAVPEVYSWCNKL

>Ccap_XP_020713613

MKLPERLLAKIVKDSESLHGNLSKICAASRPAAAAAASPVARQITSTDSRHFHDVVGDVVCPSGVRGIDH

LRDPRLNKGLAFTLEERQTLGIHGLQPARFKTQEEQLELCKIAVSRYTEPLNKYLYLVDLQDRNERLFYR

FLAENIEHLMPIVYTPTVGLACQRFGLIYRRPRGLFISFNDRGYVFDVIKNWPESDVRAICVTDGERILG

LGDLGANGMGICVGKLALYTALAGIKPHRCLPILLDVGTNNIDLLEDPLYVGLRQKRIVGKEYDDFLDEF

MQAIVKRYGQNTLIQFEDFGNHNAFRFLDRYRDSYCTFNDDIQGTASVALAGIYASTRITGKSITDYTFM

FAGAGEAAIGIADLIVNAMVAEGVPKEEARQKIWMTDINGLLTTTRQEGSLSDHQKNYAKEVAPMKNLQE

IVEKVKPNVLIGASAAAGIFTPEILRLMATNNERPVVFALSNPTHKAECTAEQAYTNTEGRVIFSSGSPF

PPVEYNGKTYNPGQGNNAYIFPGVALGVITTGTHHISDDMFLIAARELANFVDQSDLDRGSLYPSLNAVR

EVSMRIAESVTKCAYDKGLASTYPEPSDKRKWLEDQLYNFNYQSSMPVTWPWPRMPYVKTRPLEPTILFS

DSK

>Tdal_comp152369

RQYHEVVGDTLCPSQVMGIDHIRDPRLNKGLAFTLEERQTLGIHGLQPARFKTQEEQLALCKIAVNRYTDSLNKYLYLTDLSDRNERLFYRFMAENIKDMMPIVYTPTVGLACQRFGLIYRRPRGLFITINDRGHVFDVIKNWPEPDVRAIVVTDGERILGLGDLGANGMGIPVGKLALYTALAGIKPHQCLPILIDVGTNNKDLLEDPLYVGLRQKRVSGREYDDFIDEFMEACVRRYGQNVLIQFEDFGNHNAFRFLDKYRNSFCTFNDDIQGTASVAVAGLYASKRLTNMSFSDYTFLFAGAGEAALGIAELVVKAMVEEGLGLEEARNKIWMCDIDGLLTKDRPDEKLEGHKKNFAKDHKPTKNLQEIVTEIKPNVLIGASAATGLFTPEILRLMGQNNKRPIVFALSNPTSKAECTAQQAYENTDARVIFSSGSPFPPVKIKGKTFYPGQGNNAYIFPGVALGVIATGTHHIPDEMFLIAAQELANMLEDKDLEHGSLYPDLSNIREVSIRIAMGVTKSAYDLGLASTYPEPQDKRKWLEKQLYNFNYDSSMPVTWSWPRMPYIKTRELVP

>Tdal_comp160968

HGTAGESDVPSKISGLEHLKNKKYNKGLAFCLEERQLLGIHGLLPAVVKTEEEQVTHSLILLNRLENNLDKYMYLSDLAERNERLFYKVLASDVSKMMPIVYTPTVGLACQNFSKIFTHPKGMYITLNDSGHIYDVLKNWPETDVRAIVVTDGERILGLGDLGANGMGIPVGKLSLYTALAGIKPSQCLPITLDVGTNNQSLRDDLLYVGLRRNRLSGVVYDEFIDEFMHAVVRRFGQNCALGIAGLCLMALIDNGLTEEEAKKRIWMVDSKGLIVVDRPKGGLTSHKLRFAQNHEPIDCLAEVVKAVKPTVLIGAAAVGGAFTQDILESMALYNDIPIIFALSNPTDKAECTAEEAYTHTKGTCIFASGSPFQPVEYNGKLLHPGQGNNSYIFPGVALGVICAGMLTIPEEIFLIAAEKLASLCTPSDLAKGSLYPPLHKITKCSVEIAISVMDYAYKNGLATVRPEPADKAKFIRAQMYNPNYQPTFPKTWL

>Dant_CL1299_Contig4

GDNDVPGNVSGLQRLQQKKFNKGLAFTLEERQILGIQGLLPARVKTEEEQVQHSLILLDRLENDLDKYMYLNNLAERNERLFYKVLSSDVAKMMPLVYTPTVGLACQKFSLIFQYPKGMYINIQDKGHVYEVLRNWPETDIRAIVVTDGERILGLGDLGANGMGIPVGKLSLYTALAGIKPSQCLPVTLDVGTNTESILNDPLYIGLRQKRTTGPEYDEFIEEFMTSVVRRFGQNCLIQFEDFANANAFRLLEKYRHNYCTFNDDIQGTASVAVGGLLASLKIKNTDLKDNKILFLGAGEAALGIANLCLMALMKEGLTKAEAKSRIWMVDSKGLIVKDRPAGGISEHKLHFAQDYPPVDTLAEAVEQLKPSVLIGAAAIGGAFTKEILEQMADFNEMPIIFALSNPTSKAECTAEEAYKYTNGKCIFASGSPFEPVEYMGKTFHPGQGNNSYIFPGVALGVLCAGMLTIPEEVFLMSAERLADLIEPEDLARGSLYPPLQKITECSIEIASFIMEYAYKNDLATVRPEPEDKREFIKSQMYDLSYSSSIPEVYSW

>Dant_CL1299_Contig3

YMYLNNLAERNERLFYKVLAYDVAKMMPLVYTPTVGLACQKFSLIFQYPKGLYITIKDKGHVYDILRNWPEMDVRAIVVTDGERILGLGDLGANGMGIPVGKLSLYTALAGIKPNQCLPVTLDVGTNTELLLNDPLYIGLRERRTTGCVYDEFIEEFMVSVVRRYGQNCLIQFEDFANANAFRLLAKYRNNYCTFNDDIQGTAAVAVGGLLASLKI

>Dant_CL2262_Contig1

DDFIDEFMKAVVKRYGQNTLIQFEDFGNHNAFRFLDKYRNTYCTFNDDIQGTAAVAVAGLYASKRITGKSFVDYTFLFAGAGEAAIGIADLVVRAMVAEGVPIDVARSKIWMVDINGLLTTTRQVGNLEGHKLNYAKDVEPMTNLEDIVTQIKPNVLIGASAATGIFTPKILQTMAANNERPVIFALSNPTSKAECTAEQAYQNTDGRVIFSSGSPFPPVTINGKTFSPGQGNNAYIFPGVALGVIATGTHHIPDDMFLIAAQELANFVDQADLDRGSLYPPLNTIQEISMRIAMGVTKCAYDTGLASTYPEPQDKRKWLEDQIYNFNYESSLPVTWVWPRMPYIKTRKESPLLAAIK

>Tcas_XP_969151

MFALIPRSLVTNVSSRSAVSLSGQKATVQEAVKWLIDSKKDYHTVSGDTITPSKVMGIDHLRDPRLNKGLAFTLEERQAL

GIHGLQPARFKTQEEQLELCRISVMRYQENLNKYLYMTELHDRNEKLFFRLLSENIEMLMPIVYTPTVGLACQRFGLIYR

RPRGLFITINDRGHVYDVLKNWPESDVRAIVVTDGERILGLGDLGACGMGIPVGKLALYTALAGIKPHHCLPIVLDVGTN

NQTLLEDPLYVGLRQKRLSGPEYDSFVDEFMQAAVKRYGQNVLIQFEDFGNHNAFRYLDKYRGVYCTFNDDIQGTAGVAV

SGLLASARVTGKKISENKFLFLGAGEAAIGIADLCVKAMQVEGLSLEEARGKIWMMDIDGLLAKGRPEGKLDGHKAYYAK

EHKPVKDLASVVNEVKPSVLIGASAASGAFTPEILKSMASFNDRPIIFALSNPTDRAECTAEQAYNNTDGRCIFSSGSPF

PPVTYKGTTFYPGQGNNAYIFPGVALGVILARIHHIKEELFLLAAQAVADHVNDSDIQKGSLYPPLSCIRECSVDIATRI

LDYSYQEGIATVYPEPQDKKAYVMQHLYNYNYESALPTTWPWPNAPEIKTRPIEPTKLMA

>Amel_XP_006563718

MFVLQRSILSPSSRSCTGSWARMLPPTHPAVKDIQQKEIHEVSGDVIPINMVKGIGHLRDPRLNKGLAFTLKERISLGIH

GLQPPRFKTQEEQLALCKASVMKYTEDLNRYLYLVELQERNERLFFRLLSENIEQMMPIVYTPTVGLACQKFGVIYRRPR

GLFITIYDKGHIYEILNNWPEQAVRAICVTDGERILGLGDLGACGMGIPVGKLALYTALAGIKPHQCLPITIDVGTNNEQ

LRNDPHYIGLNKPRSHGAEYDELIDEFMAACVKKYGQNVLIQFEDFGNHNAFRFLDKYRDKYCTFNDDIQGTAAVAVAGI

LASKRITKKRISENKFVFLGAGEAAIGIANLCVKAMEVDGCSTQQARDNIWMMDIDGLLVKDRPEGNLEGHKIWYAKKYK

VMKSLFEVVKEIKPSVLIGASAAAGAFTTDVLKEMARNNERPLIFALSNPTSKAECTAQQAYDHTNGKCIFSSGSPFGDV

HYGGKIYKPGQGNNAYIFPGIALGVIATGCHHITEDLFLISAQAVADHVKDEHLEVGSVYPPLGTIRECSIDIAVRIAEY

AYAKSGLASEYPEPKDKRQFIVSKMYDANYDSPLPNVYDWPGDYAKPRVLPDKDTVEIRHDLKHL

>Amel_XP_395280

MKIANMSSKHLLKRLDCHFTSFFHQSSHLRYAIRELEQDAIHNRQLNDRLQSSTRLVCDSNSSTMSSVQRDQLGQRGHGD

AMCSNLLRGLDHLKNPRLNKGMAFSLQERQILGIHGLLPAAVKSQDEQLELCRLNLDRYDNDLSKYIYLIGLLDRNEKLF

YRLLEQNVEKMMPLVYTPTVGLACQKFGLVYRRPRGLFISIHDKGYVYDILNNWPEHDVRAIVVTDGERILGLGDLGAYG

MGIPIGKLSLYTALAGIKPHQCLPITLDVGTNTQSLLDDPLYIGHRHKRITGKEYDDFLDEFMEAIIKRYGQNTLIQFED

FGNANAFRLLNKYRDHYCTFNDDIQGTASVAVAGLLASLRVTNTKLSENTIVFQGAGEASLGIAWLCVMAMQKEGISIEE

AKSKIWMVDSKGLIVKNRASGGLTEHKLHFARDDKHIDSLLEVVKYAKPTVLIGAAAVGGAFTTEILEEMANNNEKPIIF

ALSNPTSKAECTAEQAYIATKGRCIFASGSPFAPVTYDGKTFYPGQGNNSYIFPGVALGVICSGMRIIPENTFLIAAKSL

ADMVSNEDLEKGNLYPPLQDIQKCSLTIAVDVMKYGYENDIATVHPQPKDYKEFIKAQLYDTTYKPSIPPIYNWPNL

>Tcas_XP_969226

MLYIRRINSRNLKLPNSSHCSKVSVVRAYVKMLDYFTKLVGNNSGENGRLLNTGNQKVNDSSNVHTVKMSTYERDRLGQR

GAGDNAATSSTSGLDRLKNASLNKGMAFTLEERQILGIHGFLPPRVKTQEEQVEHCRMCLERLDNSLNKYLYLMNLLDRN

EKLFYRLVGENVQDIMPLIYTPTVGEACQKFGLVYRRPRGLFITIHDKGHVYDILKNWPESDIRVIVVTDGERILGLGDL

GACGMGIPIGKLSLYTALAGIKPHQCLPITLDVGTNTEKMLNDPLYIGLKQNRVRGQQYDDFIEEFMQSVVRRWGQNTLI

QFEDFGNANAFRLLGKYRDSYCTFNDDIQGTASVAVAGILASLRATKTRLSDNTLVFQGAGEASLGIAALCVMAMKAEGT

PEHEARKRIWMVDSKGLIVKNRPDGGITEHKEPFAQDHAPIKTLAEVVQQVKPTILIGAAAVGGAFTPEILREMAKNCKK

PVIFALSNPTSKAECTAEQAYENTDGTCIFASGSPFDPVTYKGRTYYPGQGNNSYIFPGVGLAAICVGMRTIGEDVFLIS

AETLANLVSEADLEKGSLYPPLSTIQEVSVKIATRLGEYAYKKGFATVCPQPDDMEAFIRAQMYNTDYIPAVPCTYPFP

>Aaeg_XP_021693932

MLARQVIASGASTALKSISAATASPKRSLSKAAITAIVQQQQTRDYHEVTGDIISPSMIQGIDHLRDPRL

NKGLAFTLEERQTLGIHGLQPARFKSQEEQLELCRISISRYQEDLNKYLYLVDLQDRNEKLFFKLISEDV

EKMMPIVYTPTVGLACQKFGLIYRRPRGLFVTINDRGYVYEVLRNWPEHDVRAIVVTDGERILGLGDLGA

CGMGIPVGKLALYTALAGIPPHQCLPIVIDVGTNNQDLLEDPLYVGLRHKRVSGKEYDDFIDEFMEAVVK

RYGQNTLIQFEDFGNHNAFRFLDKYRDTYCTFNDDIQGTASVAVAGLLASKRITGKKISENTFCFLGAGE

AAIGIADLVVKAMQAEGCGLQEARDKIWLFDIDGLLAKGRPEGRLGGHKAFYAKDHRVMKNFAELISEIK

PSVLIGASAAGGAFTPEILQTMAKNNARPIIFALSNPTSKAECTAQAAYENTEGRCIFSSGSPFPPVKYG

GKTFSPGQGNNAYIFPGVALGVIVTGTHHIPEDIFLIAAQAVADHVSQDDLDKGSLYPPLSAIRECSLEI

AVGVTKYAYEKGLASTYPEPQDKLAYIKSHLYNFNYESAMPVTWKWPPQQEAPKRPITPTKLQA

>Aaeg_XP_001658020

MRIIIVLSRSRGVPVLTRRLFPKSPSFIIHRTFNDSNGGLIMPGQLRGYDHLREPRLNKGLAFTLEERQS

LGIHGLLPAQIRTLDEQLELCRLAFSRYKEDLHKYVYMVELHDRNEKLFFRLLTEDIERMMPIVYTPTVG

LACQKFGFIFRRSVGLFVTINDRGHVYEVLKNWPEMDVRAIVVTDGERILGLGDLGAQGMGIPVGKLALY

TALAGVKPHQCLPVVIDVGTNNEEFLQDPLYIGLKQKRVRGEEYDALIDEFMAAVVKRYGQNTLIQFEDF

GNKNAFKFLDRYRNSYCTFNDDIQGTAAIVVAGLMASQKIVGRSMSEHKFLFLGAGEAAIGIADLLVRAM

ESDGIPEDQAQEKIWMFDKQGLLAKEREEGELEGHKIRYAKDKCPTKDFLEVVKKVQPSVLIGASAAAGA

FNKDVLRTMADCNERPIIFALSNPTAMAECTAQEAYDFTDGRCLFASGSPFPPVEFNGKTFVTGQGNNAY

VFPGVALGVIASGIHHISDDIFLKAAEVVANKVSEEDLSKGLLYPPLSSIKQCSIEIAVGVLEYAYQKGI

ASRYPEPEDKLALIKSHQYSFCYERSLPATWRWPQNHCKPQGTVKDPCLNDS

>Aaeg_EAT42717

MAAKVCNYPSSKSFMNGRYQPESASAKTHTTAGMMTSGSHSAASRGNAAAVCSELSNGKQAPPTSWATTG

TQSACKFSSTANSNNTMSECSRDRLGQWPVESDSEVAGGVSGFGRLHNGRFNKGLAFTIEERQALGLHGL

LPATVRSEEEQVQHCLTLLNRYENDLDKYIYLVGLLDRNERLFYRVLSSDIGNMMPLVYTPTVGLACQKY

SMIYQQPKGMYITINDKGHVYDVLKNWPETDVRAICVTDGERILGLGDLGANGMGIPVGKLALYTALAGI

KPHQCLPVTLDVGTNTQSILDDPLYVGLRHKRVTGEAYDEFIEEFMQAAVRRFGRNCLIQFEDFGNSNAF

RFLDKYRDNYCTFNDDIQGTASVAVAGLLASLRVTKTKLSENTVLFQGAGEAALGIAELCVMAMRQDGMS

EEDARKRIWLVDSKGLIVKDRPKGGISGHKHRFAHDHAPVDTLAEAVKDIKPTVIIGAAAIAGAFTPEII

RTMADLNERPIIFALSNPTSKAECTAEQAYEHTDGRCVFASGSPFPPVTYKGKTYYPGQGNNSYIFPGVA

LGVICAGADTIPEDIFLISAQRLAEIVTEDDLERGSLYPPLETIQDCSIKIAVRIMEYAYKNGLACTKPE

PSDKEAFIRGQMYDLTYKSALPVMYPWPKL

>Cqui_XP_001850914

MALKTLSLCRLLATAKLVITPSRAFREVTGDIIVPGQVKGISYLQDSRLNKGLAFSLEERQTLGVHGLLP

ATIRTLDTQVQLCRTVFSRFEDDLNRYVYLMDLHDRNETLFFKLVADHIELMLPIVYTPTVGLACQKFGL

IYRKPRGLFVTVNDRGHVYQVLKNWPESDVRAIVVTDGERILGLGDLGACGMGIPVGKLALYTALAGIKP

HQCLPVTIDVGTNNEEFQNDPQYIGLRQPRVQGDEYDELVDEFMRAVVKRYGQNTLIQFEDFGNQNAFRL

LDAYRDKYCTFNDDIQGTAAVVLAGLYASERLTGKKIADHTFVFLGAGEAALGIADLVIQAMVEEGLSKE

QAHEKVWMFDKLGLLASDRPEGDLGGPKKSFIRDHQPTKDFANFIKEVKPSVLIGASAAAGAFTQEVLQT

MATNNANPIILALSNPTAKAECTAQEAYEQTEGRCIFASGSPFPPVEFAGKILEPGQGNNAYIFPGVALG

VIASGAHHIPPEMFVIAAKVVGEMTTDEDLDRGSLYPPLRRIPECSLEIAIRVIECAYQRGIASFYPEPE

DKREFVRSTQYFARYESALPVTWPWAERKRGAASVMKLNSPACGGKARG

>Agam_XP_310951

MATTVFNGYPIMQRCQPEMDSSSSSNATKQSAAKEKPSAPSRHRVVGNELQSVKQQPSQRQQEPSRVPDR

AEANQVPTHYSQPGAIGDRRSRTADRTAPTNSSSSSIASSNISMSTPSFNRDRLGQWAAESDGEIAGSIA

GFDRLRNGKFNKGLSFSIEERQVLGIHGLMPAIVRTEEEQVQHCLELLKHYTDPLNKYIYLMGLLDRNER

LFYRVLASNIGEMMPLVYTPTVGLACQKYSLVYQQPKGMYITINDKGHVYEVLKNWPESDVRAIVVTDGE

RILGLGDLGANGMGIPVGKLALYTALAGIKPSQCLPVTLDVGTNTQSILDDPLYVGLRHRRVTGPAYDEF

VQEFMEAAVRRFGRNCLIQFEDFANSNAFRFLDQYRHDYCTFNDDIQGTASVAVAGLLASLRITRTKLAD

NRVLFQGAGEAALGIAQLCVMAMKRDGLTEEEARQRIWLVDSKGLIVKDRPTGGISGHKHLFAHEHAPVG

TLLEAVRELKPTILIGAAAIAGAFTPEVLRTMAANNERPVIFALSNPTSKAECTAEQAYEHTDGRAVFAS

GSPFAPVTLGDRTFHPGQGNNSYIFPGVALGVLCAGASTIPEEIFLLSAQRLAEIVTDDDLERGSLYPPL

ENIKDCSIKIATHIMEYAYAEGLACTQPEPDDKEAFVRAQMYDASYKPAVPSVYAWPKL

>Agam_XP_003436731

MQARSVILNAKQTVDALRTLVGTASPKKSLTKAATSAAVQAFQARDYHEVTGDIIVPSMVQGIDHLRDPR

LNKGLAFTLEERQILGIHGLQPARFKSQEEQLELCRISISRYQEDLNKYLYLVDLQDRNERLFFRLISED

VEKMMPIVYTPTVGLACQKFGLIYRRPRGLFVTINDRGHVFDVLRNWPEPDVRAIVVTDGERILGLGDLG

ACGMGIPVGKLALYTALAGIPPHQCLPIVIDVGTNNKDLLEDPLYIGLRHQRVQGKEYDEFIDEFMQAVV

KRYGQNTLIQFEDFGNHNAFRFLDKYRNTYCTFNDDIQGTASVAVAGMLAAKRITNKRISDNTFLFLGAG

EAAVGIADLVVKAMQAEGTGLQEARDKIWMFDIDGLLAKGRPEGRLGGHKAYYAKDHGVMKNFADVVKEV

KPSVLIGASAAGGAFTPEILQAMGQFNERPIIFALSNPTSKAECTAQAAYDNTEVRLGRCIFASGSPFPP

VQYGGKTFITGQGNNAYIFPGVALGVIVTGTHHIPEDMFLIAAQVVADHVSEADLEKGSLYPPLSAIKEC

SMDIAVGVTNYAYQKGLASTYPEPEDKKSYIESHLYNYNYQSAMPVTWPWPKQHESSKTREINPTKLQA

>Cqui_XP_001862425

MLSRYNQLIRYSIPLQSFSGRYQPESVVSGKTHTTAGGGGQYRNGHRAAPNSAVCSELSSGKEATLLNNK

SPATGTPNYSTMASSDRLGQWPAESDGEVAGEVTGFARLRNGRFNKGLAFSIEERQALGIHGLLPAIVRS

EEEQVQHCLTLLNRYENDLDKYIYLVGLMDRNERLFYKVLGSDIGNMMPLVYTPTVGLACQKFSMIYQQP

KGMYITINDKGHVYDVLKNWPEKDVRAICVTDGERILGLGDLGANGMGIPVGKLALYTALAGIKPHQCLP

VTLDVGTNTQSILDDPLYVGLRHKRITGDAYDEFIDEFMEAAVRRFGRNCLIQFEDFGNSNAFRFLDKYR

DNYCTFNDDIQGTASVAVAGLFASLRVTKTKLSENKVVFQGAGEAALGIAELCVMAMRKEGISEQEARQR

IWLVDSKGLIVKDRPKGGISGHKHRFAHEHAPVDTLAEAVKELKPTVIIGAAAIAGAFTPEIIRSMAEFN

ERPIIFALSNPTSKAECTAEQAYEHTDGRCVFASGSPFPPVTYKGKTYHPGQGNNSYIFPGVALGVICAG

AATIPEEMFLISAQRLAEIVTDEDLERGSLYPPLELIQDCSIKIAVRVMEYAYANGLACTKPEPSDKEAF

IRAQMYDLGYKSALPAIYPWPKL

>Cqui_XP_001850913

MLARQVILSGTSNALKTISAATASPTKSLMPKSTAPSAAPTRRYHEVTGDIISPSMVMGIDHLRDPRLNK

GLAFTLEERQVLGIHGLQPARFKNQEEQLELCRISISRYQEDLNKYLYLVDLQDRNEKLFFRLISEDVEK

MMPIVYTPTVGLACQKFGLIYRRPRGLFVTINDRGYIYEVLRNWPESDVRAIVVTDGERILGLGDLGACG

MGIPVGKLALYTALAGIPPHQCLPIVIDVGTNNTALLEDPLYVGLRHNRIQGKEYDDFIDEFMEAVVKRY

GQNTLIQFEDFGNHNAFRFLDKYRDTYCTFNDDIQGTASVAVAGLLASKRVTGKKISENTFLFLGAGEAA

IGIADLVCKAMQVEGLTLQEARDKIWLFDIDGLLAKGRPEGRLGGHKAFYAKEHKVMKNFAEVVNEVKPS

VLIGASAAGGAFTPEILQAMGKNNDRPIIFALSNPTSKAECTAQAAYDNTEGRCIFASGSPFPPVQYGGK

TFLPGQGNNAYIFPGIALGVIATGTHHIPEDMFLIAAQAVADHVAQEDLDKGSLYPPLGAIRDCSLEIAV

GVTKFAYEKGLASTYPEPKDKLAYIKTHLYNFNYESAMPVTWKWPPQKEVTGGSINPTQLQA

>Pcoq_MNCL01000112

GLAFTLEERQTLGIHGLQPAKIKTQEEQLELCKISINRYQEDLNKYLYLIDLQXISSYATVVSFQDRNEKLFFRLVSENVEELMPIVYTPTVGLACQKFGLIYRRPRGLFITVNDRGYIFEVLKNWXDVRAIVVTDGERILGLGDLGACGMGIPVGKLALYTALAGIQPHQCLPILIDVGTNNKDLLEDPIYIGLRQPRVTGEEYDCLIDEFMQAVVKRYGQNTLIQFEDFGNHNAFRFLDKYRNHYCTFNDDIQGTAGVVVAGLYASQKVVGKKFIDHTFLFVGAGEAAIGIADLCVRAMQADGISQEVHFYXIWMVDIDGLLTKDRPKEKLEGHKIYYAKDHEPMKKLEDIVNKVKPSVXLIGASATTGLFTAKILRAMATFNERPIVFALSNPTSKAECTAQQAYDNTDXGRVIFASGSPFGPVTIGHKTFYPGQGNNAYIFPGVALGVIATMTHHIPDDFFLIAAQELAESVSKEDLEKGSLYPPLKAIREVSIRIAMGITKYAYNKG

>Pcoq_MNCL01000048

QGLAYSIEERQILGIHGHLPAVVKSEEQQIEHCMILLDRLENELDKYMYLTGLSQRNERLFYKVLASNISKCMPLVYTPTVGLACQKFSMIYQYDRGMFITIKDKGHVYDVLXFQNWP-EIDVRAIVVTDGERILGLGDLGANGMGIPVGKLSLYTALAGIKPHQCLPITLDVGTNTQSILDDPLYIGLRRKRVTGKEYDDFIEEFMQAVVRRFGQNCLIQFEDFGNANAFRLIEKYRNSYCTFNDDIQGTASVALAGLLASLRITKTKLKDHTILFQGAGEAALGIANLCVMAIMREGASEEEAKRKIWLVDSKGXFAHDHDPVDTLHQAVELVKPSVLIGAAAIGGAFTHEILEMMSTFNEKPVIFALSNPTSKAECTAEQAYKXGKCIFASGSPFPPVEYEGKTFYPGQGNNSYIFPGVALGVLCAGMLTIPEEVFLMSAKTLADLCSEKDLENGSIYPPLSTITNCSVKIATEIVTYAYCKGLCQCIDIVEIGF

>Cnas_XP_031623425

MFARNILKCGSPKKQNLSAIGRALGAASSDKSQLVVPSRSYAFEVTGDIICPGFVQGIDHLRDPRLNKGL

GFTLEERQVLGIHGLQPARFKCQEEQIELCKISINRYQEDLNKYLYLIDLQDRNERLFFRLVSENVAQMM

PIIYTPTVGLACQKFGLIYRRPRGLFITINDKGHVYDVIRNWPEPDVRAIVVTDGERILGLGDLGACGMG

IPVGKLALYTALAGIKPHQCLPILIDVGTNNKDLLEDPLYIGLRQKRVTGPEYDEFIDEFMAAVVKKYGQ

NTLIQFEDFGNHNAFRFLDKYRDNYCTFNDDIQGTAAVAVGGLYASSRLTGKTFADSTILFVGAGEAAIG

IADLCCKAMEADGISTEEARGKIWMCDIDGLLTTTRKEGSLEGHKKHYAKDCQPMKDLAQVVEEVKPTIL

IGASATPGLFTPEILKKMAKFNERPIVFALSNPTSRAECTAEQAFQNTDGRIIFCSGSPFPPVTINGKTY

KPGQGNNAYIFPGVALGVIATLMHHIPDDVFLIAARELAASVRDEDLAVGSLYPPLDSIREVSLKIAIGI

TKYAYCKGLASTYPEPEDKKKWLEDQLYNFNYESSMPVTWKWPQPTPMKTRELKPVKLTEREEI

>Cnas_XP_031639001

MYAIVHEQYFFYNKCSTHAQQQPPLYQMPNQSNIDRINTTDTVEYALSKGKATEVSSRNNVNNYCGSELY

SDCKSLKKSADSDTKKMSTETSHRDRLGFWGNDSEVAAAISGLDRLRLSRYNKGLAFTREERQLLGVHGF

LPAVVKSAEEQVKHCIILLNRYENDLDKFVYLMGLYDRNERLFFRVLTSDIGSMMPLVYTPTVGLACQKY

SLIYQYPKGLFISIHDKGHIYDVLKNWPETDVRAIVVTDGERILGLGDLGANGMGIPVGKLSLYTALAGI

KPHQCLPITLDVGTNTQSILEDPLYIGLRQRRVTGKEYEDFIEEFMQAVVKRFGQNCLIQFEDFGNANAF

HLLNKYRDSYCTFNDDIQGTASVALAGVLASLKSTGTQLKENTILFQGAGEAALGIANLCVMAMCKEGLT

REEAVKHVWLVDSKGLIVKDRPKGGINEHKAHFAHEHEPVNTLAEAVDQIKPTILIGAAAIAGAFTPEIL

AKMAELNAKPVIFALSNPTSKAECTALDAYTYTNGKCIFASGSPFPPVEYMGKTYYPGQGNNSYIFPGVA

LGVICAGASIIPEDIFLVSAERLANLVTDADLDVGSLYPPLDTIRDCSMKIAVSVMNYTFEKGLASVRPE

PENKEEFISSQTYDLNYPSAIPCTYAWPKL

>Mdes_JXPD01006196

NIFYRNSECSTHAHHHSSSYQSAKQSNGKRINTSETVEYALSKGKATEIGTSNPAQSHCRINNNCCNNKHTIGVHSQQSADSNAKGFTNKMSSEQNQRDRLGLWGHDSEVAGAVSGLERLRLPRFNKVCEIYR----IIAFHRFQGLAFTHEERQLLGIHGLLPAIVKTDDEQVKHCITLLNRYENDLDKFIYLMGLFISDLASVRPEPADKEAFVKSQTYDLGYPSALPITYPWPKLXLQDRNERLFFRVLASDIEKMMPLVYTPTVGLACQKYSLVFQQPKGLFITIHDKGHIYDILKNWPETDVRAIVVTDGERILGLGDLGANGMGIPVGKLSLYTALAGIKPHQCLPITLDVGTNTQSILDDPLYVGLKQRRISGDEYYEFVDEFMQAAVKRFGQNCLIQFEDFGNANAFHLLNKYRDSYCTFNDDIQLSENKILFQGAGEAALGIASLCVMAMIKEGLSKDLASKRVWLVDSKGLIVKNRPKGGINEHKAHFAHEHAPIDTLAEAVEELKPTILIGAAAIPGAFTPEILAKMAEYNAKPVIFALSNPTSKAECTAEQAYTYTNASGSPFPPVTYMGKEYRPGQGNNSYIFPGVALGVICAGASTIPEEVFLVSAERLADMVTDSDLEIGSLYPPLNTIRDCSIKIAVEVMKYVYENGMFVV

>Smos_VUAH01006225

HNECSTHAHRQPHSHHSTKQSNSKRINTFDTVEYALSKGTATEFGESNYVNNYCRINCSKYNSVNTSFEKSADSDVRNMSSDHNCRDRLGLWGNDSEVASAISGLNRLRFSRYNKXGLAFTREERQLLGVHGFLPAVVKSEEEQVKHCVLLLDRYENDLDKFIYLMGLYXDRNERLFFRVLASDIGRMMPLVYTPTVGLACQKYSLIYQYPKGLFITIHDKGHVYDILKNWPETDVRAIVVTDGERILGLGDLGVNGMGIPVGKLSLYTALAGIKPHQCLPITLDVGTNTQSILDDPLYIGLRQRRVTGQEYEDFVEEFMQAVVRRFGQNCLIQFEDFGNANAFSLLNKYRGSYCTFNDDIQXGTASVALAGVLSSLKATGTKLKENTILFQGAGEAALGIANLCVMAMRKEGLSEDDAVKRVWLVDSKGLIVKGRPKGGINEHKAQFAHEHEPVDTLTEAVDEIKPTILIGAAAIPGAFTPEILAKMAELNAKPVIFALSNPTSKAECTAEDAYTYTNXGKCIFASGSPFPPVEYMGKTYYPGQGNNSYIFPGVALGVICAGALTIPEDIFLVSAERLAXSLVTDADLDVGSLYPPLDTIRDCSVTIAVAVMNYAYEKGXGLASVR-EPANKEEFIKSQMYDLSYPSAIPVTYPWPKL

>Smos_VUAH01000508

QGLAFTREERQVIGVRGFFPAVVKSEEEQVKHCILLSNRYENDLDKFVYLMDLYDRNERLFFRVLTSDIGRMMLLVYTPTVGLACQKFSLIYQYSKGLFITIHDKGHVYDILKNWPETDVRAIVVTDGERILGLGDLGANGMGIPLGKLSLYTALAGIKPHQCLPITLDVGTNTQIILDDPLYIGLRQKRATGKEYEDFIEEFMQAVVRRFGQNCLIQFEDFGNANAFHLLNKYRDSYCTFNDDIQ

>Smos_VUAH01000002

GLGFTLEERQVLGIHGLQPARFKSQEEQIELCKISINRYQEDLNKYLYLIDLQDRNERLFFRLVSENVXVAQMMPIIYTPTVGLACQKFGLIYRRPRGLFITINDKGHVYDVIRNWPEPDVRAIVVTDGERILGLGDLGACGXGMGIPVGKLALYTALAGIKPHQCLPILIDVGTNNKDLLEDPLYIGLRQKRVVGSEYDEFIDEFMAAVVKKYGQNTLIQFEDFGNHNAFRFLDKYRDNYCTFNDDIQGTAAVAVGGLYASSRLTGKTFADATIMFVGAGEAAIGIADLCCKAMEADGISTEDARGKIWMCDIDGLLTTTRKDGSLEGHKQHYAKGCQPMKDLAEIVKEVKPTVIXILIGASATPGLFTPQILQDMAKFNERPIVFALSNPTSRAECTAEEAFQNTEGRVIFCSGSPFPNVTYNGKTFKPGQGNNAYIFPGXTNFKCIFAKINPFIYHLTGVALGVIATLMHHIPDDVFLIAARELAASVRDEDLEVGSLYPPLDTIREVSLKIAIGITKYAYCKGMVIAR-RVQFKYNFLFNFSSISFDLSXGLASTYPEPDDKRKWLEDQLYNFNYESSMPITWKWPQ

>Mdes_AEGA01025357

DRNERLFFRLLSENVEELMPIVYTPTVVGLACQKFGLIYRRPRGLFVTIHDKGHVYDVIRNWPEPDVRAIVVTDGERILGLGDLGACGMXGMGIPVGKLALYTALAGIKPHQCLPILIDVGTNNRDLLEDPLYIGLRQKRIVGPEYDEFIDEFMAAIVKKYGQNTLIQFEDFGNHNAFRFLDKYRDNYCTFNDDIQGTAAVAVGGLYASSRLTGKTFADATILFVGAGEAAIGIADLCCKAMEADGISTEXDARGKIWMCDIDGLLTTTRTEGSLEGHKTYYAKDHPPIKDLAQVVKEIKPTILIGASATPGLFTAEILQDMGKFNERPIVFALSNPTSRAECTAEQAFQNTKGRVIFCSGSPFPPVTYNGKTYKPGQGNNAYIFPGVALGVIATLMHHIPDDVFLIAARELAASVRDEDLAMGSLYPPLGAIREVSLKIAIGITKYAYCRGM
