# Supplementary material for: Genomic signatures of globally enhanced gene duplicate accumulation in the megadiverse higher Diptera fueling intralocus sexual conflict resolution
Source: PeerJ. 2020 Oct 12;8:e10012. doi: 10.7717/peerj.10012 (PMC7560327; doi:10.7717/peerj.10012)
Supplement: Supplemental Information 9 [file peerj-08-10012-s009.zip › Colt protein sequences 2020.docx]

>Dmel_colt_AAF51209

MTTTENVSTERKANPVKSFLTGGFGGICNVLSGHPLDTIKVRLQTMPRPAPGEQPLYRGTFDCAAKTIKNEGVRGLYKGMSAPLTGVAPIFAMCFAGYALGKRLQQRGEDAKLTYPQIFVAGSFSGLFSTLIMAPGERIKVLLQTQQGQGGERKYNGMIDCAGKLYKEGGLRSVFKGSCATMLRDLPANGLYFLVYEALQDVAKSKSETGQISTASTIFAGGVAGMAYWILGMPADVLKSRLQSAPEGTYKHGIRSVFKDLIVKDGPLALYRGVTPIMLRAFPANAACFFGIELANKFFNIVAPNF

>Dmel_CG3476

MEEVEISTEKKSNPVKSFIAGGVGGMCNVLVGHPLDTIKVRLQTMPTPPP

GQPPRYKGVIDCAARTFRYEGFRGFYRGISAPLVGVTPIYAVDFAVYAAG

KRLFQTDDHIRLTYPQIFAAGALAGVCSALVTVPTDRIKVLLQTQTVSNG

PLLYNGTIDTAAKLYRQGGIRSLFKGTCACILRDSPTGFYFVTYEFLQEL

ARKKSANGKISTTSTILSGGTAGIVFWTLAVPFDVLKSRLQSAPEGTYKH

GIRSVFRNLMATEGPKALFRGILPILLRAFPSTAAVFFGVELTNDLLKA

>Dvir_XP_002051268

MAVVENVQNTPTERKANPLKSFITGGFGGICNVLSGHPLDTIKVRLQTMPRPAPGEQPLYRGTFDCAAKT

IRNEGVRGLYKGMSAPLTGVAPIFAMCFAGYALGKRLQQRGEEAKLTYPQIFVAGSFSGLFSTFIMAPGE

RIKVLLQTQGIGPGGEKKYTGMIDCAVKLYKEGGLRSVFKGSCATMLRDLPANGLYFLVYEYIQDVAKAN

SKTGEINTASTIFAGGAAGMAYWLLGMPADVLKSRLQSAPEGTYKHGVRSVFKDLIVKDGPLALYRGVTP

IMIRAFPANAACFFGIELANKFFNLVAPSF

>Dvir_XP_002051123

MAAATADHKANPVKSFIAGGFGGICSVLTGYPLDTIKVRLQTMPLPAAGQPPKYKGIIDCTVKTFSTEGV

RGFYRGISAPLVGVTPIYAVDFAVYAAGKRLFQTDEHVKLTYTQIFIAGVGAGICSALVTVPTDRIKVLL

QTQPVTGPVMYNGMLDTAIKLYRQGGLRSLFKGTCACVLRDSPTGVYFVVYEGLQDLARRRSATGQITPT

STIFAGGTAGIAFWSLAVPFDVLKSRLQSAPEGTYTHGIRSVFRELMATEGPKALYRGVLPILIRAFPST

AAVFVGVELANDVLNA

>Cpip_XP_001843922

SENVSPVKYFLSGGFGGICTVLAGHPLDTIKVRLQTMPLPAPGQAPQYRGTLDCAKQTIAREGFRGLYKGMSAPMTGVAPIFAMSFFGFGVGKRLQQSSPDEELTNTQLFAAGAFSGIFTTTVMAPGERIKCLLQIQQGGTGPQKYNGMVDCAKQLYAEGGIRSIYKGAFATLLRDVPASGMYFLTYEYVKKAMAPKADEKQDAAVGLMGTIFAGGMAGIANWAIGMPADVLKSRLQTAPEGTYKNGIRDVFRELMKNEGPLALYKGVTPVMLRAFPANAACFIGFEIFMNFLNFVAPNL

>Ccap_XP_004519322

MSEAPAQERKANPFKAFLSGGFGGICNVLSGHPLDTIKVRLQTMPRPAPGQQPIYTGTFDCAAKTIKNEGVRGLYKGMTAPLTGVAPIFALCFAGYALGKRVQQTGENAKLTYTQIAIAGSFSGLMSTVITAPGERIKCLLQVQQASGGERKYKGMLDTAVQLYKEGGIRSVYKGSCATLLRDLPANAFYFLAYEYIQDKAKEQTGSEKISVTSTIFAGGAAGIAYWLVGMPADVLKSRLQTAPAGTYPKGVRSVFKDLMRRDGPLALYRGVTPMLRAFPANAACFFGIELANDAYDAIAALL

>C_capitata_Contig2199_4_2

LIDVPANAIYFATYEAFQDFIKQTFPQMHMEVLSAIVSGGMAGIAYWVVGMPPDVLKSRLQTXXXTAPPDKYKHGIRSVFAELMRTDGPLALYRGVSPVMLRAFPANAACFFGIELANAFFRIVT

>Dant_Unigene4480

TKMSDSTERKANPIKSFLSGGFGGICTVLSGHPLDTIKVRLQTMPRPAPGQPPMFKGTFDCAAKTIKNEGFLGLYKGMSAPMTGVAPIFALCFAGYALGKRVQQTDGNTRLTYSQTFVAGSFSGLLSTIITAPGERIKCLLQIQQASTGADKKYNGMLDCFKKLYKEGGIRSIYKGSCATLLRDLPANGCYFLVYEYIQEAAKKQSGSDQVSLTATLFAGGSAGIAYWLVGMPADVLKSRLQTAPEGTYKHGIRSVFKELMLKDGPLALYRGITPIMIRAFPANAACFFGIELANKFFNKVA

>Tdal_comp121665

ERKANPIKSFLSGGFGGICNVLSGHPLDTIKVRLQTMPTPKPGEAPLYRGTFDCAAKTIKNEGVRGLYKGITAPLTGVAPIFAMCFAGYALGKRIQQTGDNTKLTYTQIFVAGGFSGLFSTCIMAPGERIKCLLQVQQASGGERKYHGMVDCAIKLYKEGGIRSIFKGSCATMLRDIPANGIYFLVYEYLQAKAKEKSGSEHVSMSSTLFAGGFAGISYWILGMPADVLKSRLQTAPEGKYKHGVRSVFKELIVKDGPLALYRGVTPIMLRAFPANAACFFGIELANKFFRSVAPNF

>Tdal_comp158528

EAKINPVKALVTGGFGGICNVLSGHPLDTIKVRLQTMPKPNPGEPPLYAGTWDCAKKTIQKEGPLGLYKGMSAPLTGVVPIFAMCFAGYALGKRLQQSDANTKLTYTQIFIAGGFSGVFSAFVMAPGDRIKSLLQIQQGSGGEQKYNGLFDCAIKLYKEDGIRSVYKGLCATLLRDIPANGTYFLTYEIISEMLTKKIGDDTTSKVAAISIISGGASGMTYWILGLPADVLKTRVQTAPPGKYPHGVRSAFPDLLKNDGPLALYRGMTPIMLRSFPANGACFLGIEMMNKFLKFVVPNF

>Aaeg_gi_78216204_gb_CH477216

SENVSPIKYFLSGGFGGICTVLAGHPLDTIKVRLQTMPLPATGQSPMYAGTLDCAKKTIRNEGFRGLYKGMSAPIAGVAPIFAMSFFGFGVGKRLQQTTPDEELSNLQLFAAGAFSGVFTTTVMAPGERIKCLLQIQQGGNVPQKYNGMVDCAKQLYAEGGIRSIYKGSFATLLRDVPASGMYFLTYEYVKKAFAPKEGEKDDAGKALLITIFAGGMAGIANWAIGMPADVLKSRLQTAPEGTYRNGIRDVFRELMKNEGPLALYKGVTPVMLRAFPANAACFIGFEVFMKFLNFVAPNL

>Gmor_GMOY007568

AQERKANPLKSFIGGGVGGVCTVVTGHPLDTIKVRLQTMPRPSPGEQPMYTGTFDCARKTIKNEGYRGLYKGMSAPLVGVTPIFALCFAGYSLGKRVQQTEDSTKLTYRQIFVAGSFSGLLSTVITAPGERIKCLLQVQQASAGERKYNGMLDCAFKLYKEGGIRSIYKGSFATLLRDLPANGAYFVTYEYIQAQAKRLTGSNEVSMAATLLAGGSSGIAYWIVGMPADVLKSRLQTSPPGYYKHGVRSAFKDLMKTEGPLALYRGIGAVMIRAFPANAACFFGIEL

>Ppap_PPATMP000683

GGIGLLGTIFAGGMAGIANWAIGMPADVLKSRLQTAPEGTYKNGIRDVFAELMKREGPLALYKGVTPVMLRAFPANAACFIGFEAFMKFLNFVAPNL

>Mdom_MDOA011608

SDSTSEKKANPAKSFLSGGFGGICTVISGHPLDTIKVRLQTMPRTGPGEAPLYKGTFDCAAKTIKNEGFLGLYKGMSAPIVGVAPIFALCFAGYALGKRVQQTEGNTKLNYRQIFVAGSFSGLLSTIITAPGERIKCLLQIQQASGAERKYNGMLDCAGKLYKEGGIRSIYKGSCATLLRDLPANGVYFLAYEAIQNYSKKQSGSDQVSLAVTLFAGGSAGIGYWLVGMPADVLKSRLQTAPEGTYKNGIRSVFKELMVKDGPLALYRGITPIMIRAFPANAACFFGIELANKFFDKFFPNF

>Agam_XP_316164

HAMSENKSPIKYFLSGGFGGICTVLAGHPLDTIKVRLQTMPLPAAGQAPLYAGTLDCAKKTIAREGFRGLYKGMSAPITGVAPIFAVSFFGFGLGKRLQQKTPDEELNYTQLFAAGAFSGIFTTTVMAPGERIKCLLQIQQGGNSPQKYSGMVDCAKQLYAEGGMRSIYKGAFATLLRDVPASGMYFLTYEYIQRALAPKAGEQKDASIGLLGTIFAGGMAGIANWAIGMPADVLKSRLQTAPEGTYPNGIRDVFRELMRREGPLALYKGVTPVMLRAFPANAACFIGVEVFMKFLNVVAPGL

>Tcas_XP_967309

PIQYFICGGFGGICTVVVGHPLDTIKVRLQTMPIPKPGEKPLYSGTLDCLKTTVRNEGIRGLYKGMGAPLLGVAPIFAISFMGYGVGKKIFGPGDGQHYSYLQYFTAGAFSGIFTTTIMAPGERIKCLLQIQQASTGPKTYSGPVDVVKKLYKEGGIRSIYRGSGATLLRDIPASGMYFLTYEAIKDYITDHGKESPSILGTIFAGGAAGIANWAVGMPPDVLKSRLQTAPEGTYPNGIRDVFKKLMLTEGPGALYKGITPVLLRAFPANAACFVGFELCKTFL

>Amel_XP_006560286

SEKENLIKYFLSGGFGGICTVIVGHPLDTIKVRLQTMPIPGPNGVLLYNGTIDCARKTIAKEGIRGLYKGMGAPLCGVAPIFAISFYGFGLGKQLVQRNNEELTSFQLFYAGAFSGIFTTIIMAPGERIKCLLQTQQGIKSKYSGPIDCMKQLYKEGGIKSIYKGTCATLLRDVPASGMYFMTYECLKKWMSSEEGKLGILQTIMAGGFAGITNWIVGMPPDVLKSRLQSAPDGTFKNGIRDVFIILMKEEGPKALYKGCVPVMLRAFPANAACFLGFEIAMNFLNWV

>Pcoq_MNCL01000057

TEGGTMGTSVNPAKYFISGGFGGICTVIAGHPLDTIKVRLQTMPKPGPGQEPMFKGTMDCALKTIKNEGFMGLYKGVQAPLVAFAPIFAVSFFGFGVGKRLLQREGEPMRYIDYFFAGAFSGIFTTVLMAPGERIKCLLQIQQEGEKKYDGMIDCAKKLYKEGGIRSVYKGTCATLARDLPAAGVYFCTYEIVTDQIKQYKGTDQIGFLPTIFAGGLAGIIFWVPGMPPDVLKSRLQTAPEGKYPHGIRSVFKELMQVDGPMALYRGVTPVMLRAFPANAACFFGFEVAMKTLNYLFPN

>Pcoq_MNCL01000031

YPQVRLQTMPTPAPGQEPMFKGTFDCAAKTVKNEGFKGLYKGMSAPLTGIAPIFAISFFGFGLGKKLLQK-DNEKLSYTDFFLAGAFSGIFTTTIMAPGERIKCLLQIQQGGTGPKKYNGMLDCAQKLWKEGGLRSIYKGTCATLLRXKAPEGKYPKGVRSVFKELMKVDGPVALYRGVTPVMLRAFPANAACFFGFELCMKFLNDIAPN

>Mdes_gi_309241387_gb_GL501437

SEHTSPIKYFLSGGFGGICTVVAGHPLDTIKVPVPVAGGSPMYAGTYDCAKKTVQKEGFRGLYKGIXXXWVGMSAPLTGVAPIFAMSFFGFGVGKRLQQKTPDQKLTNVQLFAAGAFSGIFTTSIMAPGERIKCLLQIQQGGSGPQKYNGMVDCAKQLYKEGGIRGVYKGSFATLLRXXXLKFCFFFSCLDVPASGMYFLSYEYVKEFTANKFGTEGGWALMGTILAGGSAGIANWAVGMPADVLKARLQTAPAGSYPNGIRDVFRELMQREGPLALYKGITPVMLRAFPANAACFIGFEIAMKFLNFVAPN

>Cnas_XP_031627939

MSENQTSPIKYFLSGGFGGVCTVIAGHPLDTIKVRLQTMPLPAPGQLPQYAGTYDCARKTVQREGFRGLYKGMSAPLTGVAPIFAISFFGFGLGKRLQQKTPEEKLTNVQLFAAGAFSGIFTTSIMAPGERIKCLLQIQQGGGAPQKYNGMVDCAKQLYKEGGIRSVYKGSVATLLRDIPASGLYFLTYEYVKEFAAREFGTEGSRGLIGTIFAGGSAGIANWAVGMPADVLKSRLQTAAPGTYPNGIRDVFRELMTKEGPLALYKGVTPVMLRAFPANAACFIGFEISMKFLNFAAPNL

>Smos_VUAH01000029

SNVPSPIKYFLSGGFGGXGICTVIAGHPLDTIKVTVXVRLQTMPLPSPGQLPQYTGTYDCARKTVQREGFRGLYKGXGMSAPLTGVAPIFAMSFFGFGLGKRLQQKTPDEQLTNTQLFAAGAFSGIFTTSIMAPGERIKCLLQIQQGGNAPQKYNGMVNIDLRQIKQXVDCAKQLYKEGGIRNVYKGSFATLLRXLFTDVPASGLYFLTYEYVKDLAAREFGTEGSLALIGTIFAGGSAGIANWAVGMPADVLKSYVAPNL
