# Supplementary material for: Genomic signatures of globally enhanced gene duplicate accumulation in the megadiverse higher Diptera fueling intralocus sexual conflict resolution
Source: PeerJ. 2020 Oct 12;8:e10012. doi: 10.7717/peerj.10012 (PMC7560327; doi:10.7717/peerj.10012)
Supplement: Supplemental Information 9 [file peerj-08-10012-s009.zip › Hexokinase protein sequences 2020.docx]

>Dmel_HexA

MDRELNGAMKSVSINSAQLNGHNNGGGLDETDRVGVAQGAALSATEKITT

TTAAATKSATATTNATTATATTTNLTTHSPQQIALLSAAEKSKMVHELCQ

QLLLTDEQVQELCYRILHELRRGLAKDTHPKANVKCFVTYVQDLPNGNER

GKFLALDLGGTNFRVLLIHLQENNDFQMESRIYAIPQHIMIGSGTQLFDH

IAECLSNFMAEHNVYKERLPLGFTFSFPLRQLGLTKGLLETWTKGFNCAG

VVNEDVVQLLKDAIARRGDVQIDVCAILNDTTGTLMSCAWKNHNCKIGLI

VGTGANACYMERVEEAELFAAEDPRKKHVLINTEWGAFGDNGALDFVRTE

FDRDIDVHSINPGKQTFEKMISGMYMGELVRLVLVKMTQAGILFNGQDSE

VLNTRGLFFTKYVSEIEADEPGNFTNCRLVLEELGLTNATDGDCANVRYI

CECVSKRAAHLVSAGIATLINKMDEPTVTVGVDGSVYRFHPKFHNLMVEK

ISQLIKPGITFDLMLSEDGSGRGAALVAAVACREDILNGKK

>Dmel_HexC

MLDAEVRELMQPFVLSDYQVQEVYSRFCLEVARGLKRSTHPQANVKCFPT

YVQDLPTGDEMGKYLALDLGGTNFRVLLVSLKGHHDATVDSQIYAVPKDL

MVGPGVDLFDHIAGCLAKFVEKHDMKTAYLPLGFTFSFPCVQLGLKEGIL

VRWTKGFDCAGVEGEDVGRMLHEAIQRRGDADIAVVAILNDTTGTLMSCA

HRNADCRVGVIVGTGCNACYVEDVENVDLLRADFKKTKRSVIVNAEWGAF

GEGGQLDFVRTEYDREVDEKSLNRSEQLFEKMTAGMYLGNLVRLVLLRAL

ERKLIFKQSSRRPEFASVLQRNEEVFETRYISEIEDDSFPEFASTRKIVK

NLFGLEKASVEDCQTLRYICECVAKRAATLVAIGVSGLVNRTSNRRVIVG

MDGSVYRYHPKFDAYMRQTLQKLVKADKEWDIMLSEDGSGRGAALVAAVA

SKTK

>Dmel_Hext2

MRKSTRLLTHSLFGPVFKILFHNKTVCGGCNRKMPSLVNTEIEAAVKGFLIDQEKMTEVVERMTKEIKMGLAKDTHARAVIKCFVSHVQDLPTGKERGKYLALDLGGSNFRVLLVNLISNSDVETMSKGYNFPQTLMSGSGKALFDFLAECLSEFCHSHGLENESLALGFTFSFPLQQQGLSKGILVAWTKGFSCEGVVGKNVVSLLQEAIDRRGDLKINTVAILNDTVGTLMSCAFYHPNCRIGLIVGTGSNACYVEKTVNAECFEGYQTSPKPSMIINCEWGAFGDNGVLEFVRTSYDKAVDKVTPNPGKQTFEKCISGMYMGELVRLVITDMIAKGFMFHGIISEKIQERWSFKTAYISDVESDAPGEYRNCNKVLSELGILGCQEPDKEALRYICEAVSSRSAKLCACGLVTIINKMNINEVAIGIDGSVYRFHPKYHDMLQYHMKKLLKPGVKFELVVSEDGSGRGAALVAATAVQAKSKL

>Dmel_Hext1

MANTFNPEEDFPEVYKVCKLFNPSIDDLEKIKNAMDREITMGLSRDHHDRSTVPCHLSYVQDLPTGRERGQFLALEMMPTNCRIMLVKFSSERDIYTSSKCVIMPHTVAAGRGTEVFTFLATSIANFVKEKKVDKDNLPLGIAFAFTLKKLALDVGILVSWTKEFGAQGAIGKDVVQLLRDALAKFPEISVDVMGIINVGAGSLLALCWAQPDTRIGLIMGSIANSCYVERVERCETYEGDEYRKLMIINSDWAHFGDTGQLDFIRNEYDRQLDTESINPGTRIYEKFSGALCMGELVRIIVLRLMKSGAIFAEDRRDYIGIQWKLDMVSLIEIVSDPPGVYTKAQEVMDKFRIRHCKERDLAALKYICDTVTNRAAMLVASGVSCLIDRMRLPQISIAVDGGIYRLHPTFSTVLNKYTRLLADPNYNFEFVITQDSCGVGAAIMAGMAHANKYKTDAKLFTMDY

>Aaeg_AAEL009387_1

EIREQCQELILSDKQIEEIMRRLLKEINRGLGKATQPEADIKCFITYVQDLPNGKEKGKFLALDLGGTNFRVLLIHLKDENDFEMLSKIYAIPQSIMLGSGTQLFDHIAECLANFMKEHSVYEEKLPLGFTFSFPLTQLGLTKGILARWTKGFNCSGVVGEDVVQLLKDAIARRGDVQIEICAILNDTTGTLMSCAWKNHNCRIGLIVGTGSNACYVEKVENCEMFDGPKDPKKEHVLINTEWGAFGDNGALDFVRTEYDREIDHFSINPGRQIQEKMISGMYMGELARLAIVKFTRAGLLFGGVGSDILFKRGQFFTKYVSEIESDKPGTYMYCRDVLDELGLEHATDEDCANVRYICECVSSRAAHLVSAGIAALINKMDEKSVTVGVDGSVYRFHPKFHDLMKAKIRQFVKPDISFDLMLSEDGSGRGAALVAAVA

>llon_LLOTMP000608

LLVELNKGLKKKTHAEADIKCFVTYVQDLPNGKETGKFLALDLGGTNFRVLLIHLKAENEFEMLSKIYAIPKNIMVGTGKQLFDHIAECLANFMKEHQVYKERLPLGFTFSFPLTQLGLTKGLLVRWTKGFDCEGVVGVDVVQLLKDAIARRGDVQIEVCAILNDTTGTLMSCAWKNHNTRIGLIVGTGSNACYVEKVENAEMFDVPENKKPHVIINTEWGAFGDNGALDFVRTEFDRDIDENSINPGSQLQEKMISGMYLGELARLVIQKFTKSGHLFGGKGSELLFKRWKFFTKYVSEIESDKPGVFTNCREVMEELGLGHATDEDCANVRYICECISRRAAHLVSAGIAVLINKMEEPNVTVGVDGSVYRFHPKFHDLMMEKTRQYVNPGINFDLMLSEDGSGRGAALVAAVASRE

>Gmor_GMOY013300

VVEEICKPFIVSDDVYRKIRDVFQNEIKKGLCKYTHESASVKCFLTFVEKLPSGCERGKFLALDIGGINFRILLVNINSGENLKIEAANYELPESLMTGTGRDLFDFLAECLSAFIYKHELQKEELSLGFTFAFPLKQTDLSKGVLITWTKAFSCSGVVNHDVVDLFKQAINRRDDIRINNIVILNDTTGTLISCAWNYREAKIGLIIGSATNMCYLEKTKHIELFKGGVNASPTMIINCESGNFGSDGSLDFVRTPIDIALDQNSVNAGEQIYEKMISGMYLGEIVRLILLECVNAGAMLNGVQSEELRTSMSLDVKHMSEIEAAEPGNDSASRKIFEIMGYKRPSDEDCEHLRYICNVISTRSAYMGAATLATLVNRVGDPFVVIGVDGAVYRMYPNYPERLRKKLRDLARPEYQFCLKVAEDGSGRG

>Gmor_GMOY013299

KIVKDICKQFVLSDDVYKQIKEMFLGEIKRGLCKYTHESACVKCFMTFVEKLPSGCERGKSLALDVDETRCRVLYINLQGDRDFRMYSQNYPIPPQILVGPGRDLFDFFVECIADFVYDHNLQNDELSLGFNFGFPLNQKSIKKAILMTWTRDISSAGVVGRDVVALLQDAINRRGGLRISNIVIANDTTGTLVSCAWKYREAKIGLVVSTGFNMCYLEKTKYLQLIRNNVNTSPTMIINCESGAFGNDGTLDFMRTPIDITLDKNSVHVGEQLFEKMISGMYLGEIARLTMLECIKAGGMMQGDFSEEVRTPMIFDVEDMSQIEADGPGNYAVTRKIFQKMGYSEPTNDDCENLRYICTVVSTRSANLIASCLACLIDRVGDPYIIIGVKGSIYETYPNFSTRLERKLKRLVRPEYEFDLVPAEGDSGQGAALLAA

>Gmor_GMOY007230

TDKAVSRVSNKATSSEAEAVNDDNILCHKRAIESFASASVAEKRKMILDLCQQLVLTDEQNKELCHRILHEIKNGLAKETHKKADVKCFVTYVQDLPNGKERGKFLALDLGGTNFRVLLIHLKEEHDFQMESRIYAIPEHIMVGSGKQLFDHIAECLCNFVTEHEVKYERLPLGFTFSFPLKQLGLTKGLLVTWTKGFNCEGVVNEDVVQLLKDAIARRGDIKIDVCAILNDTTGTLMSCAWKNPSCKIGLIVGTGSNACYVEQVKECEMFDGDTHGKPHVVINTEWGAFGNSGSLNFVRTEFDEEIDRHSINPGQQIFEKMISGMYMGELVRLIMVKMVQAGVLFKGHSSDVLMTRGQFFTKYVSEIEADEPDTYSNCHMVLEELGLTEVTDDDCANVRYICECVSKRAAHLVSCGIATLINKMNEPHVTVGVDGSVYRFHPKFHSLMVEKIAQIVKPGLTFELMLSEDGSGRGAALVAAVASRED

>Gmor_GMOY013303

IVEDFCKPFVLNEETSQQVSDLFLAEIKKGLCKYTQPKADIKCYPTHVEKLPTRCEQGKFLALDVGGSNFRISLFIIQDVDNTKVESQDFQLPPKVLTGPGEHLFEFFAECMSNFIQTHELQEEEFHLGFTFSFPLMKTSLKEAILISWSKDFQCKDVVGHDVVAMLEAALSRRDNIHIKDIFVLNGTTATLISCAWKHKETKIGVTIDRDTNAVYEEKMKHIQLFSEERFNSTMLINTQWGSFGNHGALNFMRSPIDFALDESSTNPHEAIFEKMVAGMYIGEIVRLTMIECINAGALLKGNLSEQIRKQMVFDTKHMSQIENEKGDAYQSTRQILETLGYKEATNEDCENIRYICNKVSTRSAELIAICLACLIDRIGDPYIVIGIDGEMYSSYPNYHERLRKKTKQLKNSSILQTFSMAE

>Gmor_GMOY013302

LNEILTPFDITKDEMMKIKDLILKELKLGLKRDTHNTADTKCYPTYIQSYPSGCEHGMFLVTTIHAVKVHVLFFHLKGENDYRLDEESSDIPEGINQAVELFDFIVEKLHKLVKSLNLEREPLPLTMVLPYPLLQINLASAILLKFTTKLKIAGMENKDVGQMMRESMRRHPNIRFELTAVINDVTSAFMSAAWRHKNVRISFIVGAATNAGYWEKVSNIESVIQTRKPEMLVNTDIAEFGSSGQLEFLATEFDEALEKLSPTKGQNIFEKMASAWYMSELSRRVIIKCINENIIFGGQSNVQLNRQDALKFANVQGTLVEADQYLYMSLMLDKLGINLPSETDCARIHHIMEKVVTRSASLVAAAIVAMIEIIDEPDIKIGLDGEVCNSLPIYHNMIRSKIDSILKPEHTYELVEANDEHGRGGAITASLILQEDYI

>Cqui_CPIJ008049-RA

KIREQCQELILTDKQMEEIMRRVLKEINNGLHKETQPTADVKCFITYVQDLPNGKEKGKFLALDLGGTNFRVLLIHLKDENDFEMLSKIYAIPQSIMLGSGTQLFDHIAECLANFMKEHAVYDEKLPLGFTFSFPLTQLGLTKGMLARWTKGFNCSGVVGEDVVQLLKDAIARRGDVQIEICAILNDTTGTLMSCAWKNHNCRIGLIVGTGSNACYVEKVENCEMFDGYKDPNKQHVLINTEWGAFGDNGALDFVRTEYDREIDHFSINKGRQIQEKMISGMYMGELARLAIVKFTKAGLMFHGIISEKIQERWSFKTAYISDVESDAPGEYRNCNKVLSELGILGCQEPDKEALRYICELFGGVGSDILFKRGQFFTKYVSEIESDKPGTYSYCRDVLDELGLEHATDEDCANVRYICECVSSRAAHLVSAGIAALINKMDEKSVTVGVDGSVYRFHPKFHDLMVQKIRQFVKPDISFDLMLSEDGSGRG

>Adar_ADAC004619PA

IREQCKELILTDKQIEELMRRIIKEINRGLSKETHAEADVKCFITYVQDLPNGKEKGKFLALDLGGTNFRVLLIHLKDENDFEMLSKIYAIPQNIMLGSGTQLFDHIAECLANFMKEHSVYEERLPLGFTFSFPLTQLGLTKGILARWTKGFNCSGVVGEDVVQLLKDALARRGDVQIDICAILNDTTGTLMSCAWKNHNCRIGLIVGTGSNACYVERVENCDLFDGPKSGPGIKQHVLINTEWGAFGDDGALDFVRTEYDREIDQHSINPGRQLQEKMISGMYMGELARLAIVKFTKAGLLFGGVGSDILFKRGQFFTKYVSEIESDKPGTYTYCRDVLDELGLDHATDEDCANVRYICECVSSRAAHLVSAGIAALINKMDEPSVTVGVDGSVYRFHPKFHDLMVQKIRQFVKPSISFDLMLSEDGSGRGAALVAAVACRE

>Ppat_PPATMP000132PA

RILAEINRGLKKETHAEADIKCFVTYVQDLPNGKETGKFLALDLGGTNFRVLLIHLKGENDFEMLSKIYAIPKNIMIGTGKQLFDHIAECLANFMKEHEVYKERLPLGFTFSFPLTQLGLTKGMLVRWTKGFDCEGVVGEDVVQLLKDAIARRGDVQIEVCAILNDTTGTLMSCAWKNHNTKIGLIVGTGSNACYVEKVENAELFDVPNNKKPHVLINTEWGAFGDNGALDFVRTEFDREIDENSINPGKQLQEKMISGMYLGELARLVIQKFTKSGHLFKGKGSELLFQRWKFFTKYVSEIESDSPGVFTNCREVLEELGLGHATDVDCANVRYICECVSRRAAHLVSAGIAALINKMGEPIVTV

>Dant_Unigene2949_GAWI01007422.1

ASASVAEKRKMVHELCQQLILTDEQINELNYRILHEIKKGLAKDTHQKANVKCFVTYVQDLPNGNERGKFLALDLGGTNFRVLLIHLKEDHDFQMESRIYAIPQHIMLGSGIQLFDHIAECLANFMNEHDVYKERLPLGFTFSFPLTQLGLTKGLLVTWTKGFNCAGVVNEDVVQLLKDAIARRGDVQIDVCAILNDTTGTLMSCAWKNHNCKIGLIVGTGSNACYVERVEEAEMFDAGANGKPHVLINTEWGAFGDNGALDFVRTEFDEDVDSHSINPGKQVFEKMISGMYMGELVRLAIVKMVKAGVLFKGVDSDVLMTRGQFFTKYVSEIEADEPGVFTNCRLVLEELGLNNATDEDCANVRYICECVSKRAAHLVSCGIATLINKMNEPQVTVGVDGSVYRFHPKFHNLMVEKITQLVKPGITFDLMLSEDGSGRGAALVAAVACREDV

>Dant_Unigene8342_14485_GAWI01012776.1

KPLVYSDSQLQEVYKRFCNEIRKGLAKSTHSQADTKCYVTYVPDLPTGDEVGKYLALDLGGTNFRVLLVTLKGSHEAVVESMIYAVPKDIMLGPGIELFDHIAQCLATFVEEHKVSNELLPLGFTFSFPCIQLGLTKALLVRWTKGFQCAGVEQEDVARLLKEAIARRGDLAIHVIAILNDTTGTLMSCAHRNPECRIGVIVGTGCNACYVERVEEVELLEPEYKTHNKHVIVNCEWGAFGDGGVLDFIRTDYDKVIDRNSLNAGTQLFEKMISGMYLGELVRLILIDAIQRNLLFVLSQHKQEYIKILSNKEGCFETSMISDIEADTFPECKNTRNILKQLFGIEKASVEDCQKLRYICECVSQRAANLVAVGISGLINKINEPKVVVGIDGSVYRYHPKFDAYMRATMKKLVDP

>Tdal_comp158884

TPEEIPPYANASVAEKYKMVHELCQQLILTDEQILELNYRILHEVKKGLRKDTHDKANVKCFVTYVQDLPNGNERGKFLALDLGGTNFRVLLIQLKEDHDFQMESRIYAIPQHIMLGSGQQLFDHIAECLANFMSEHNVYKERLPLGFTFSFPLLQLGLTKGLLVTWTKGFDCSGVVNEDVVQLLKDAIARRGDVQIDVCAILNDTTGTLMSCAWKNHNCKIGLIVGTGCNVCYVERVEEVELYDGEDNGKPHVLINTEWGAFGDNGALNFVRTEFDEEVDSHSINPGKQVFEKMISGMYMGELVRLVIVKMVKAGVLFRGQNSDVLMTRGQFFTKYVSEIEADEPGTYTNCRLVLEELGLNNATDDDCANVRYICECVSKRAAHLVSCGLATLINKMDEPSVTVGVDGSVYRFHPKFHNLMVEKISQLVKPGISFDLMLSEDGSGRGAALVAAVACRED

>Tdal_comp157604

LKEFMKEFTLSDSQLKEVYNRFGNEIRKGLNKDTHAKADTKCFITYVQDLPNGDERGKFLALDLGGTNFRVLLVTLKGHHDATIVSNTYPIPQEILVGTGEQLFDHIAKCLDDFVKAQQVGESYLPLGFTFSFPCVQLGLKKSMLVRWTKGFQCSNTENEDVGRLLKEAIARRGNTEIEVLALLNDTTGTLMSCAHRNANCRIGLIVGTGCNACYVENVDCVDLLEPELKRTKKKVIINVEWGAFGDSGLLEFVRTDYDRIVDKMSPNIGQQLYEKMISGMYLGELVRLALHKATKENLMFLRSANKNKILEVLNKEVGCFKTEMLSRIEADTFPDFTNTKNILKELFGSEKVNIEDCQKLRFMCECVAQRAANLVAVGLAGLVNRINEPNVVIGVDGSVYKHHPKFDAYMRVTMRKFVTPGIKFDIMLSEDGSGRGAALVAAVASRQ

>Tdal_comp160205

VLLRAIKKNKFAMTDLKIREFTSCMSLKFPQVHEIYKRFCNQIRSGLGKHSHATADTKCYVTFVQDLPTGQEVGNYLALDLGGSNFRVLLVTLKGNYDSEQVSVTYPIHSDLMIGTAEELFDYIASCLENFVKLHNIEDDKLYLGFTFSFPCTQINLTQAILRRWTKGFQVAGVVDEDISVLLKDAIDRRGTLNIEPVAIVNDTVGTLISCAYFNNQCRIGLIVGTGCNACYLEHVKCIERMDAQYREKKNMIINTEWGAFGEGGDLEFIRTDYDKQVDDTSPNPKEQIFEKMVAGMYLGKLVGYMTVRAINESYILVENEEKHKIVDVLEKDPDVFETYRISQIECDLSTELKSIQTIMRELFEVTFISHDDCLNFKHICESVTRRSATLVAVNISALINKINEPRVVVAVDGSLFRLHPRYDSYMRETLRKFVNPKIVYDLVLSEDGSGRGAALIAAVV

>Tdal_comp147884

ELKRSLSKNLFHLSDLMCIPTAITSLPQGYERGRSLTIVINETQVRVSLVVLMSYGDHKEVTEVYELPDKYTRCSLQKLLTYVAFCLESFLYDITSLPQTEIPLGVIFPLPCYYRSLSGVQVIKNFGKFNLRFKPYVDVGLLLEKTIDKLQTIKIGKISMMNDATNAAFLGAYNAKNVRIGLHNGDYCNACYFEALKCVERADTEYKRKSNMLINIEWGALGEKGYLEYFFNKHDIYIDEMSSNRGKQLFNKLVSGKYIIEMWRVVLTDTMIKGLIFKKNYDKVKIIDAISYKNALTLQLMGYLERCGMSNIKHLLDKFEARQISYDDLCALKGICSVIIKRAAYIVAVPLSVLVNKIAEANITIAVDGDVFREVKSYEKHLRKVMDYFVCPKISYEFVCVDGNTACGPALL

>Mdom_XP_019891905

SVAEKRKMVHELCQQLILTDEQIDELTYRILHEIKRGLAKDTHAKANVKCFVTYVQDLPNGNERGKFLALDLGGTNFRVLLIHLKEDHDFQMESRIYAIPQHIMLGSGTQLFDHIAECLSNFMVEHNVKSERLPLGFTFSFPLTQLGLTKGLLVTWTKGFNCAGVVNEDVVQLLKDAIARRGDVQIDVCAILNDTTGTLMSCAWKNHNCKIGLIVGTGSNACYVERVEEAEMFDGASNGKPHVLINTEWGAFGDNGALDFVRTEFDEEVDRHSINPGKQVFEKMISGMYMGELVRLAIVKMVNAGVLFKGQDSDVLMTRGQFFTKYVSEIEADEPGTYTNCRLVLEELGLTEATDDDCANVRYICECVSRRAAHLVSSGIACLINKMNEPHVTVGVDGSVYRFHPKFHTLMVEKISQLVKPGITFDLMLSEDGSGRGAALVAAVACREDNLPKKK

>Mdom_MDOA012708PA

IQEICAPFEVNIEQLLQIKNQILDEIASNLETKSEYQKPTNLKCFNTFLQDFPQGCEHGRFIVVNVEEENFRIHLYQMRGQREPLVQSEFYEINSKLLENDENELFVHIANTLQMFLKKLQLEREPLAVVFVFQFPLNNQNSERVNFTEYTKRFDFKNQNKDDIDVRKLLESIIMTKCPRTQVVAVISQATAVFCAATWKYPNCAIGVYLGSEDTSMAYKEKTGNFPSPNNPCPTKPFVAVHLDWGAFGDNGCLDHIRHELDIKVDKLSPFPRKRIYEKMICGQYVGELARQIFLICTQQGFLFNGEIGKQLITPFSFNTRHICEVLAESPGNFDNTRLMFDRLGVMKPMDSECSKVHFILECLAKRSAALLAAGCACLVDKISEPDILIAIEGSFYGNCLIYQKFVSEYLRQLTGEGTRFQLKNVEDSVGLGAAVVAAITEQQKFL

>Mdom_MDOA002808

TDESPEETNDRMIKEICQALILDKEKLQQIKDVFLNDLKLGLCKYSHTKAMAQWAVTYIQDLPGGCENGTFLALDWSGVNFRIMAIYLKCKKDFHMDSKLYEIPTKLLTGPGGDLFDFLAESLSTFVKTFQVNGGDESLPLALNFGFEVKQTAINKAELVSWQGGIDCPDVVGRDIIQMLRDALERRDDVKIQILILVKDPIGTLMNGAWQDRNCKIGLNVAPATCKACYMEKLRNMTIYENPKGNTTPTMLINCEWGLLGDNGKLDFLQSKYDKDIDESSENPKENVFEKLLIDKHLGELVRLILMDCINAELLFKGNVSEQLRQEASFKSEYIFLIENENRESNDIIREILEKLGYKNPSDFDCEQTCFICHVVMKRSADLLAAIIACLIDRVGDPYTMIAIDGTLCHQFPPYNCLMSQRISQLVRPEHKFKLMLSEDGSGRGAALTAAV

>Mdom_ALHF_02218_g1275

LKEFLKPLVLSDSQLQEAYKRFCNEIRKGLSAREHEQADTKCYVTYVQDLPTGNEVGKYLALDLGGTNFRVLLVTLKGHHEAVVESEIYAVPKDIMLGPGEELFDHIAQCLATFVEGHKVASEHLPLGFTFSFPCVQLGLTKALLVRWTKGFQCSGVEQEDVGRLLKEAIARRGDLEIHVMAILNDTTGTLMSCAHRNPECHIGVIIGTGCNACYVEKVDQVELLEPEYKVNNRYVLINTEWGAFGDGGLLDFIRTDYDKEIDKKSLNAGTQIFEKMISGMYLGELVRLILLEALKRNLIFVLSQNKSAFITKLSGECECFETSMISEIEADVFPEFKKTREIIKQLFGIEKASVEDCQKLRYICECVSQRAASLVGVGLSGLINKIEEPKVVVGVDGSVYRFHPKFDAYMRATMKKLVKDNVEFDLMLSEDGSGRGAALVAAVASR

>Ccap_XP_004521010

SIAEKRKMVHELCQQLILTDEQIRELNYRILHEIKRGLNKDTHAKADVKCFTTYVQDLPNGNERGKFLALDLGGTNFRVLLIHLKEEHDFQMESRIYAIPQHIMIGTGQQLFDHIAECLASFMSEHNVYKERLPLGFTFSFPLQQLGLTRGVLSTWTKGFNCSGVVGEDVVQLLKDAIARRGDVQIEICAILNDTTGTLMSCAWKNHNCKIGLIVGTGSNACYVERVEECELFEGADNGKSHMLINTEWGALGNNGMLNFLRTEFDDEIDRHSINPGRQLFEKMISGMYMGELVRLVIVKLVKAGILFKGQDSDVLMTRGQFFTKYVSEIEADEPGNFTNCRMVLDELGLNDATDEDCANVRYVCECVSKRAAHLVSCGIATLINKMDEKHLTVGVDGSVYRFHPKFHSLMVEKITELIKPDISFDLMLSEDGSGRGAALVAAVACREDNIKTK

>Ccap_XP_004520566

QDQRLKDFLKEFIISDSQLHEVYKRFTTEIQNGLSLATRKQADIKCYMTYIQDLPTGDEKGNYLALDLGGTNFRVLHVSLKGHHEAEIESKVFVVDKELMTGHGTQLFDHIANCLAEFVNEYSLNNQHLPLGFTFSFPCVQLGLTKALLVRWTKGFNCAGSVQEDVGRLLKEAIARRGDLEINVMAILNDTTGTLMSCAHRNPECRIGVIVGTGCNACYVERVENAELLESEYKVDKTHIIVNTEWGAFGESGKLEFVRTDYDRTIDKASLNVGSQLLEKMISGMYLGELVRLVLADAISENLIFSRTSDKQSLINTLRENVCCFEARFISEIENDNFPDFQQTRTILKELFAMEKASVEDCQKLRYICECISRRAANLVAVGISSLINRIAEPHVVVGVDGSVYRLHPHFDAYMRETMRKLVNPKITFDLMLSEDGSGRGAALVAAVASK

>Ccap_XP_004533719

MSTTSELTRLREFLNELIPSEDQLVRLYQNFCAQIRKGLGSKTNKDASTKCYLTYVQDFPTGNETGKYLALDLGGSKFRVLLIHLKGDSEAEQITKIYDLTERELHSTGVELFDYIAKCLHEFVCEQKVDNEHMPLGFTFSFPCVQTGLSRATLVRWTKGFNCSDTVGEDIGLMLKAAIARRPGLRIDMVAILNDTTGTQMSCAHRNPSCHIGLIVGTGCNACYTEKVENCERLDSKYKDKPKIIINVEWGAFGEKGGVDHLFTDYDRNVDQNSINRGSQIFEKMVSGMYMGELVRLILLRAVNENLVFAQCKLRKHITDILTKKPHCIETKVLSDIANDRGDDWPLVKDVFKQVFNLNNADPDDCMKFKFICDVVVKRSGNMIGVTLSGLVNKVGESFSVIGVDGTVYRCHPNFDGYMRETLDRFVDKDYKYDMMLSEDGSGRGAALVAAVVHRD

>Tcas_XP_970645

MAAEKVDPVLKERCKDLILDLDTLREISRRFLHDVDKGLRKDTHATSIVKCFVTYVQDLPNGTENGKFLA

LDLGGTNFRVLLIELSKDHFEMRSKIYAIPQHIMLGSGEQLFDHIADCLASFMKQEQVIAETLPLGFTFS

FPLTQKGLTKGILERWTKGFNCSNCVGNDVVQLLKDAIARRGDIQIKVCAILNDTTGTLMSCAWKNPNCR

IGLIVGTGSNACYVEKQKNAELFDDEDKGSGNVIINLEWGAFGDDGALDFIRTEYDRDVDANSVNPGRQL

HEKMISGMYMGELTRLALEKFTKEGLLFEGKASDALFTRGKFYTKYVSEIEGDPAGVITSCRDILEELGI

YHATDQDCFNVRYVCECVSKRAADLASTGIATLLNKMNEARVTVAIDGSVYRYHPHFHDLMMQTISTLVN

PGIKFDLMLSEDGSGRGAALVAAVACRTQQKG

>Tcas_XP_008201713

MRLKQIIESVNDLGRLQVDQIGLNPDAENEITDEIREKCQELILSNAQLEEYKKKLLNDLKKGLGKATHP

TSIVKCFITYVQDLPDGSETGKFLALDLGGTNFRVLLIELSKNHFEMRSKIFAIPQHIMLGSGEQLFDHI

ADCLAKFAKDEKIQHEVLPLGFTFSFPLMQKGLTKGILERWTKGFNCSNVVGNDVVQMLKDAIDRRGDIQ

IEVCAILNDTTGTLMSCAWKNRYCKIGLIIGTGTNACYVEDQKNAEMFDEPDRGSGKVLINCEWGAFGDD

GALDFVRTQYDREVDEHTVNPGKQLHEKMISGMYMGELVRLALERFTNEGLLFGGKGSDQLFTRDRFYTK

YVSEIESDPPGTFTNCKEILEELGLKHATEQDCINVRYVCECISRRAAHLASAGIATLLNKMKEPRVTVG

IDGSLYRFHPHFHNLMMEKISELVEPGIVFDLMLSEDGSGRGAALVAAVASRKR

>Amel_XP_006557645

MKTKNRSLFSRMCKCYGKESHAQVQQQIEEEPSTVAKVSDEIRDICKDLVLSDEKLRQLMDTLNDQLHKGLGKETHATATTKCFPTYVQDLPQGTEKGNFLALDLGGTNFRVLLITLDEQNFDMKSKIYVIPQSLMVGTGVQLFDHIAHCLALFVKDLNLQNEVLPLGFTFSFPLDQHGLTKGYLIRWTKGFKCDDIIGEDIVDLLEQAIKKRGDVKIQICAILNDTTGTLMSCAWKNKNCRIGLIVGTGTNACYVEKLENIQTVYPENVLPGKPKMLINIEWGAFGEGTLLDFIATDIDRDVDENSINPAKQVFEKMISGMYMGELVRLLIEKAINAGLLFIGKSTNELKKRGRFYAKYVSEIENDPNGKYTNCREVLAELGLRNVTDQDCENVKYICSVVSRRAAHLASAGIATLLNKMDEDNVVVGIDGSVYRYHPHFHNLMTEKISQLQNHKFELMLSEDGSGRGAALVAAVAAGNR

>Dvir_XP_002053421

FMLNKQHMDEIVNRLTKEINMGLTRDTHPRATVKCFVTYVQELPTGNERGKYLALDLGGTNFRVLLVNLINDRDIDITGKTYAISKELQEGSGIKLFDFIARCLAEFCKEHKMEQSNTPLGFTFSFPCKQVGIDNGTLVAWTKGFKAEGVVNQNVVELLRDAIKRRGDFKVNVVAILNDTTGTLMSCAFNHRNCKIGMIVGTGSNACYVEKTANTQTFDGYLSSTKPNMIINCEWGAFGDNGVLDFIRTSYDHEVDKQSINPKKQIFEKCISGMYMGELVRLVLLELMEKEAIFKGQTSQSILIKGKFDTSFITEIESDQPGSHRNAALVMDRLGIRTNNEKDLGCLRFICETISTRSARLAACGLVCLINKMNVKDLTVGIDGSVYRYHPNYHRLLTENMNMLLKGSVKFELVLSEDGS

>Dvir_XP_002053418

KQFTLTEDQMFEVVDRLTKEIEMGLRKETNLRSTVKCYITYVQDVPSGKERGKYLALDLGGSNFRVLLVDLKSQTDVDIQSKSFVLGKNLLTGSGKRLFDYIAECLAEFCIEQRIAWDNLPLGFTFSFPCKQTAINKGVLVNWSKGFKCPDVVGHNVVQMLQQAIDSRLDLRVRVVAILSDTTGTLMACAQRQPNCRIGLIVGNVCNACYIEKTENTEMFQRYQTSRKRNMIVNCEWGSFGDNGVLDFIRTSYDKSMDQLTPDPMMQTFDKCISGVYLGEIVRQIVLDLMKKNVIFKGLMSQAIQTEWKFESRFIAEIESEPFGQYRSTAMVLNSLGIRTCNEKDLACLRYICESVTRRSAQLVACGLVCLIRKMNVCKSSVGIDGGMYRYHPNYHRLLIENMNILLKCSAEYELVLSEDGSGRG

>Dvir_XP_002053420

KMPYDPETDFPEAWKLCKPFMLTDDHLLSIRNSMTRELLNGLGRDTHDRSSIPCYLSYVQHLPTGRERGRFLALEMWPTNCRIMLVKFGSEKDIYMSSKCVIVPHTIAASRGTTLFNFLAQNIAVFVREKKVEKDNLPMGIAFAFALNKLSLDVGILVSWTKGYGAQGAVGKNVVQLLRNALDEYKDISINLNSIINIAAGSLMALSWSQTNCKMGLIIGTVTNAAYVEQSVECEMYEGDASVPLMIINTEWNNFGSNGHLDFIRTEFDKIVDAESNNPGKRYYEKCISTLYLGELVRLIIVRLMNMGVIFREHNLDYMGIQWKMEMKSIMAIDSDPPDVYVKAQEVMDKFRMRNCQERDLATLRFICQTVSTRSAKLVAAGVACLINRMNYANISIAVDGGIYRLYPRYQEVLNKHAAALINPDLKFEITIAQDSPGVG

>Dvir_XP_002055038

MDQEQLNGAMRSVSINSNGLATNQNGHNGNDETDNARVLGGGGGGGGISFGLSSGNAASKATNAATNAAT

NAATNAATDAATATETATATANANVNAIQIQTSAAGASAADKKKMVHELCQQLLLTDEQVQELCYRILHE

VRRGLAKDTHPKANVKCFVTYVQDLPNGNERGKFLALDLGGTNFRVLLIHLQENNDFQMESRIYAIPQHI

MIGSGLQLFDHIAECLSNFMAEHNVYSERLPLGFTFSFPLRQLGLTKGLLETWTKGFDCAGVVNEDVVQL

LKDAIARRGDVQIDVCAILNDTTGTLMSCAWKNHNCKIGLIVGTGSNACYVEKVDEAELFDMRDNRKPHV

LINTEWGAFGDNGALDFVRTEFDKDIDSHSINPGKQTFEKMISGMYMGELVRLVLAKMTQAGILFNGQGS

EVLFTRGLFFTKYVSEIEADEPGTYTNCRLVLEELGLSNATDGDCANVRYICECVSKRAAHLVSAGIATL

INKMDEPHVTVGVDGSVYRFHPKFHNLMVEKITQLIKPGISFDLMLSEDGSGRGAALVAAVACREDILNS

K

>Dvir_XP_002059924

MVDVEIRELMQPFVLSDYQVQEVYSRFCLEIAKGLRRTTHEQASTKCFPTYVQDLPTGDEMGKYLALDLG

GTNFRVLLVTLKGHHEATVESQIYAVPKDLMVGPGVELFDHIAECLAKFVAKHDMHNAYLPLGFTFSFPC

IQMGLREAKLTRWTKGFNCPGVEGEDVGLMLHDAIQRRGDAEIAVVALLNDTTGTLMSCAHRNPDCRVGV

IVGTGCNACYVEHVDNVDLLDTEFKRIQKQVIVNVEWGAFGDHGQLEFVRTDYDREVDRKSINRSEQLFE

KMTSGMYLGELVRLVLLRALERNKIFKLNTKRAAFVAVLQKNVDIFETKYISEVEADSFPEFPNTRRIVK

QLFGVEKATVEDCQKLKYICECVTKRAATFVAIGISGLINKIIDRRVVVGIDGSVYRYHPKFDGYIREMM

QKLVKPGKEFDIMLSEDGSGRGAALVAAVASKTK

>Pcoq_MNCL01000216

QQIREQCRNLILSKEQIDELSARFLAEIKRGLSRSTHHKADIKCFVTYVQDLPNGERGKFLALDLGGTNFRVLLIHLKGENDFEMQSRIYAVPENIMTGTGVQLFDHIAECLANFMKVHDAVXEEKLPLGFTFSFPLIQLGLTKGLLVRWTKGFNCSGVVGEDVVQLLKDAIARRNVSMIILCDVKIAVMAILNDSTGTLMSCAHRNYNCKIGIIIGGTGSNACYVERVENAELFDMPGNAKEHVLINTEWGAFGENGTLDFIRTEYDDDVDKHSINPGKQMSQEKMISGMYMGELARLVIVKFTKMGLLFGGKGSDMLFTRGQFFTKYVSEIESDPDGDHSSCRMVLEELGLSHATDEDCANVRFICECVSTRAAHLVSTGIATLINKMDEPSVTVGVDGSVYRFHPRFHDLMTKKIRELVKPHIQFELMLSEDGSGRGAALVAAVASRD

>Pcoq_MNCL01005250_1

GTGTNACYVEKVENAELFDLPGNEKPYVLINTEWGAFGENGCLDFIRTEFDREIDKNSIHVGKQMYEKMISGMYMGEFVRLVIVKFTKMGLLFGGKGSDLLFTREKFTTEYVSEIESDPEGHYSRCRMVLEELGLPHATDEDCANVRFICERVSTRAAHLVSAGIATLINKLDEPSVTVGVDGSVYRLHPRFHDLMTNKIRELVKPHIQFELMLSEDGSGRGAALVAAVA

>Pcoq_MNCL01005250_2

YLALDLGGTNFRVILMELRNSIVIREEVKHYHISDNLRIGCGDKLFDYLAECVDNFVVEQNMINDDIIMGFTFSFPMKQHSLDSGCLVTWTKTFNCPSIVDKNVVKKLRESLDKIGRNNIQVLAILNDTTGTLVQGAQLDNNTRIGIVVGTGTNACYMEKADRVKHWEIQRHDEEHVIIDVEWGAFGDNGTIDFIKTEFDRAVDDGSLNSILSRFEKYIAGKYLGELCRVIMKKLNEENLFLIGTDLCKFPAPWTFGSDNVSLIEQTLEGSDYRIKDILNKYNYIYKNDYTDDDIQILRYICGLISKRAGLFLAITTSALLERMEENNITVAIDGSVYKHHSRLRQWLHYFTNKFVPNKKVFILMLTEDGSGKGAALVAAIAKR

>Pcoq_MNCL01000057

GTGTNACYVEKVENAELFDLPGNEKPYVLINTEWGAFGENGCLDFIRTEFDREIDKNSINVGKQXYEKMISGMYMGELVRLIMEKFTKMGLIFDGKGTDLLFTREKFTTEYVSDIESDPEGVYTTCRKVLHKLRLSHATDEDCTKVRFICKSISTRAAHLVSAGIATLINKIGQTYVTVSXVGVDGSVYRLHPRFHDLMTSKIRELVKPFIKFDLMLSEDGSGRGAALVAAV

>Mdes_AEGA01030422

STIKMSTKKADANSQRIREECQELILTDDQIQELMKRIIYEIERGLSAKTHNDADVKCFITYVQDLPNGNERGKFLALDLGGTNFRVLIIHLNGENEFKMQSKIYAVPQSIMLGPGEQLFDHIAECLANFMKEHNVYSERLPLGFTFSFPLKQLGLTKGLLSTWTKGFACDGVVGEDVVQLLKDAIARRGDVQIDVCAILNDTTGTLMSCAWKNHNCRIGLIVGTGSNACYVEKIENCHLFDGNKMEKSHVIVNTEWGAFGENGALDFLRTQYDREIDDHSINRGKQIQEKMISGMYMGELVRLALVRFTKEGLLFGGESTDMLNTRGRFFTKFVSEIESDKPGSYMYCRQVLEELGLDHATDQDCANVRFICECVSTRAAHLVSAGIAALINRMDEKSLTVGIDGSVYRFHPKFHDLMTAKIRTLIKSDIKFDLMLSEDGSGRGAALVAAVACREE

>Cnas_XP_031632506

MGTKKADANSQRIRDECEELILTDEHMQELMKRIIHEIERGLSAKTHDEADVKCFITYVQDLPNGNERGKFLALDLGGTNFRVLIIHLNGENEFKMQSKIYAVPQSIMLGKGEELFDHIAECLANFMKENNVYAERLPLGFTFSFPLKQLGLTRGLLAQWTKGFACSGVVSEDVVQLLKDAIARRGDVQIDVCAILNDTTGTLMSCAWKNHNCRIGLIVGTGSNACYVEKVENCGLFDGNSKKSHVIVNTEWGAFGDNGALDFVRTQYDREIDEHSINRGRQIQEKMISGMYMGELVRLALVRFTKEGLLFDGQSTDMLNTRGRFFTKFVSEIESDKPGSYMYCRQVLEELGIDSATDEDCENVRYICQCVSTRAAHLVSAGIAALINRMDEKSLVVGIDGSVYRFHPKFHDLMTAKIRQLIKPDIKFELMLSEDGSGRGAALVAAVACREEGRK

>Cnas_XP_031618677

MSYLFQDFEKEYQVTLLDRSDVQNRKINEVLNGLFFNEPTLNRIAKQFETEMNLALNEQWNEASVLMENTYIPELPDGSEHGKFLALDLGGTNFRVILLELDNGKITNEVVKMYEIRSELRVGSEDVAVALFDHIGQCLCDFVEENDLVSVPLPLGFTFSFPMKQHSLNSATLDAWAKSFNLPTVLGTDVVERLRDSLHKLGHNHIEVVAILNDTTSTLIQGVNLDKRTRIGIVFGTGSNAAYVENADRVKHWEGKGRRGAKQVCIDIEWGAFGDKGSLDFIRTRFDDRLDETSLLPGGYTFEKYIGGKYLGELVRLVLEELQQRKLAFHNTPAIAFPKPWSFDTREISSIEEDNLNGTTNLTKDVLNEAGFKNVSEHDIAVIQHVAAVVSHRAALLVSITTSVILARLTCQDVTIAIDGSVYKKHPRMDAWLYRIIGKLNTTGKTFRLMLAEDGSGKGAALTAAIALKIDNQMIK

>Smos_VUAH01006190

MGTKKADAKSQQIRDECQELILTDEHMQELMKRVIFEIERGLSAKTHNEADIKCFTTYVQDLPNGSECGKFLALDLGGTNFRVLIIHLNGENEFKMQSKIYAVPQSIMLGKGEELFDHIAECLANFMKENNVYSERLPLGFTFSFPLRQLGLTRGLLSQWTKGFACSGVVGEDVVQLLKDGIARRGDVQIDVCAILNDTTGTLMSCAWKNHNCRIGLIVGTGSNACYVEKVENCGLFDGTSKKDHVIVNTEWGAFGDNGALDFVRTQYDREIDEHSINRGRQIQEKMISGMYMGELVRLALVRFTKEGLLFSGQSTEMLNTRGRFFTKFVSEIESDKPGSYMYCRQVLEELGLDHATDEDCENVRYICQCVSTRAAHLVSAGIAALIDRMDEKSVVVGIDGSVYRFHPKFHDLMTAKIRELIKPDIKFELMLSEDGSGRGAALVAAVACREEGR

>Smos_VUAH01003649

MGTKSADTNLQRIRDECQELILTDGHIQKLMKTIIQEIERGLSAETHNEADIKAFNTYVQDLPNGNERGKFLALDLGGTNFRVLLIHLNGKYEYEKTSKIYAVPQSIMLGKGEELFDHIADCLANFMKENNVYDEHLPLGFTFSFPLRQLGLTRGLLAQWTKGFACSGVVGEDVVQLLRDAIARRGDVQIDVIAILNDTTGTLMSCAWKNRRTRIGLIVGTGTNACYVEKVKNCELCDGTSKKENVIINTEWGAFGDNGALDFVRTKYDREIDEHSINRGRQIYEKMISGMYLGELVRLALVQFTKEGLLFSGQSADLLNTRDRFFTKFLSEIESDKPGSYKHCHQVMDELGLHNATDEDCQNVRYICERVSTRAAHLVSAGIAALINRIDEKSVMVAVDGSVYRFHPKFHDLMTAKIRELIKPDIEFELMLSEDGSGRGAALVAAVASRENSQQ

>Mdes_JXPD01005490

FNYLFLSFCRSFQISXQITEVLHGFYMDNATVDRVAKQFEIEMILSLNEQTDESSLLMENTYIPELPDGSENGKFLALDLGGTNFRVILLELDNGEIVQEIVKKYHIGSHLRVGDENVAIALFDHIGQCLCDFVAENDLVSVPLPLGFTFSFPMRQHSLSSATLEAWAKSFNLPSVIGADVVEMLRDSLHKLGHNHIDVVAILNDTTSTLVSXRIGIVFGTGSNAAYVEQADRVKHWEGKGKNINIKQVCIDIEWGAFGDKGSLEFFKTKYDQELDETSLLPGXQIRDSLRGTTKMTKEMLTDCGFNNITDHDIEVIQYVSAMISQRAAVLVSITTSDIVKRIYEKDLTIAIDGSVYKKHPRM

>Smos_VUAH01006180

EHGKFLALDLGGTNFRVLLMDLDNGKITNEVVKMYEIRSELRVG-GEDMAVALFDHIGQCLCDFVEENDLVSVPLPLGXFAFSFXLGFTFSFPMKQHSLDSATLDAWAKSFNLPTVLGTDVVDRLRDSLHKLGHNHIEVVAILNDTTGVLVRNKFSSSVFRIXDKRTRIGIVFGTGSNAAYVEQADRVKHWEGKGRRGAKQVCIDIEWGAFGDKGSLDFIRTRFDDRLDETSLXGTKHLTKEVLIEAGFKDLTDYDIMVCQHVAAVLSHRAALLVSITTSVIMARLTCPDVTIAIDGSVYKNHPRXLQFRLMLAEDGSGKGAALTAAIALKLENK
