# Supplementary material for: Genomic signatures of globally enhanced gene duplicate accumulation in the megadiverse higher Diptera fueling intralocus sexual conflict resolution
Source: PeerJ. 2020 Oct 12;8:e10012. doi: 10.7717/peerj.10012 (PMC7560327; doi:10.7717/peerj.10012)
Supplement: Supplemental Information 9 [file peerj-08-10012-s009.zip › Tim17b protein sequences 2020.docx]

>Dmel_CG1724

MEEYSREPCPFRIVDDCGGAFTMGCFGGGLFQGLKGFRNAPQGLKRRFAG

GLAAVKSRSPTIGGNFAAWGCVFSIVDCSLVHLRKKEDPWNSIMSGAIAG

GILSSRNGVAAMFGSAIIGGVLLSMIEGVGILFTRISAEQFRNSDPQNDL

ElGRAGAFASGSGMGSGDINSSPGFEFPVVQQANATS

>Dmel_Tim17b2

MEEYSREPCPHRIVDDCGGAFIMGCVGGGLFQGLKGFRNAPQGLGRRVAG

SVAAIKTKSPVIGGSFAAWGAVFSIVDCSLVHFRQKEDPWNSIVSGAVTG

GILASRNGAAAMAGSAIIGGVLLSMIEGLGIFFTRFAAEQFRNREPHIMP

DANEGYGDFNSSGFGFPGAQQATATS

>Dvir_XP_002052164

MEEYSREPCPYRIVDDSGGAFAMGSIGGGIFQALKGFRNAPQGLSRRLMGSMMAVKARSPVIAGNFAAWG

GMFSTIDCTLVHFRKKEDPWNSIISGAATGGILAARNGLASMAGSAIIGGILLSLIEGVGILLTRISAEQ

FRNPAPPIDPQDVGVNAFESDPAKGFGFGTRTANVNS

>Dmel_Tim17b1

MAEYGREPCPFRIVEDCGGAFAMGALGGGAFQAIKGFRNAPSGLGYRLSG

GLAAVRARSGLVGGNFAVWGATFSAIDCSLVYFRKKEDPWNAIISGATTGGILAARTGLTSMLSSALVGGALLALIEGVGIVVSHYSADSYRQVSPVERQQRYKQELLRQQKGVSPLAATYGEIDSSAL

>Dvir_XP_002056569

MEEYTREPCPFRIVEDCGGAFTMGAVGGAIFQAIKGFRNAPCGLPRRLAGGLAAMRARSGLVGGSFAIWG

GTFSAIDCSLVYSRGKEDPWNSIISGAATGGILAARGVTAMLSSALVGGVLLALIEGAGIAMSHYTADNY

RQVSVLERMQHYNERQLRQQQQQQQQQQQTTTTIPSFYTDPNEASLCLV

>Dvir_XP_002056572

MVEYTRQPCPIRIVEDCGCAFMMGCIGGSMFQYMRGFRNAPTGILRGLYGGLDSVKMKTPAIAGSFAVWG

ATFSTVDCTMVYYRQREDSWNSIVSGAATGGILAARNGIKAMGNGALVGGLVLAMIEGAGAAVATIYAAQ

PSCTESAHPLRPQWEQMSVAPDNSGQQSSSDALAEIERVLDKCNTYKSPTRRARQKPKYMETEDYIKQSP

SLLELVKLANIFKT

>Amel_XP_001120807

MEYNREPCPWRIMDDCGGAFTMGAICGTLFQSIIGFRNAPSGFQRRFYGGIMTVKNRVPQISGNFAIWGCLFSAIECTLIHFRSKEDPWNSILSGALTGGVLAARTGIPSMIGSATVGGIFLALVEGFGIMATRLHADAFAHHMQMYEMENLPEFHGLPPRARLGFTGAPVSTESVTVTRQVNGNGMEVDRISKGR

>Dmel_Tim17b

MEEYAREPCPYRIVDDCGGAFAMGCIGGGVFQAIKGFRNAPSGLNRRLVG

SIIAIKTRSPVIAGNFAVWGGMFSTIDCTLVHFRKKEDPWNSIISGAATGGILAARNGVPAMAGSAIIGGVLLALIEGVGILFTRISADQFKNPIPPAEDPVALGDPGRNFSFESASNRTQYQ

>Dvir_XP_002058764

MEEYARDPCPYRIVDDCGGAFAMGCIGGGVFQAIKGFRNAPSGLNKRLIGSVAAIKTRSPVIAGNFAIWG

GMFSTIDCTLVHIRKKEDPWNSIISGAATGGILAARNGIPAMAGSAIIGGVLLALIEGVGILFTRISADQ

FKNPSPPTEDPAALGDPVNNFTFGPSNNKHYQ

>Ccap_XP_004526337

MEEYAREPCPFRIIDDCGGAFAMGCIGGGVFQAIKGFRNAPSGISKRMIGSLTAVKIRSPVIAGNFAVWGGMFSTIDCTLVHFRKKEDPWNSIISGFATGGILAARNGIPAMAGSAIIGGVLLALIEGVGILFTRLSAEQFKNPAPPIEDPAVLGEAAQPFSFGQTSQHPSQYQ

>Dant_Unigene1466

MEEYAREPCPYRIVDDCGGAFAMGCIGGGIFQAIKGFRNAPSGMSRRMIGSLSAIKTRSPVIAGNFAVWGGMFSTIDCTLVHFRKKEDPWNSIISGFTTGGILAARNGIPAMAGSAIIGGVLLALIEGVGILFTRLSSEQFRNPLPPTE

>Tdal_Td_comp142682

MEEYAREPCPYRIVDDCGGAFAMGCIGGGIFQAIKGFRNAPSGISKRMIGSLSAIKTRSPVIAGNFAVWGGMFSTIDCTLVHYRKKEDPWNSIISGAATGGILAARNGIPAMAGSAIIGGVLLALIEGVGILFTRLSAEQFRTPGPPSE

>Aaeg_AAEL015575

MEEYAREPCPYRIVDDCGGAFAMGCIGGGVFQAIKGFRNAPSGFSRRLVGSLTAIKSRSP

VIAGNFAVWGGMFSTIDCTLVHFRKKEDPWNSIISGAATGGILAARNGVGAMAGSAIIGG

VLLALIEGVGILFTRISAEQFRSQPIVEDPSALGDPSQNAGAAAPSSSMPFGFGQSGQNY

Q

>Gmor_GMOY012330

MEEYAREPCPYRIVDDCGGAFAMGCIGGGVFQAIKGFRNAPSGMNRRLLGSLSAIKTRSPVIAGNFAVWGGMFSTIDCTLVHFRKKEDPWNSIISGFATGGILAARNGVPAMAGSAIIGGVLLALIEGVGILFTRLSAEQFRNPLPPSEDPSALGDPSKPFGFGQPHSNQGQYQ

>Mdom_MDOA012163

MEEYAREPCPFRIVDDCGGAFAMGCIGGGVFQAIKGFRNAPSGMSRRMLGSLSAIKTRSP

VIAGNFAVWGGMFSTIDCTLVHFRKKEDPWNSIISGAATGGILAARNGVPAMAGSAIIGG

VLLALIEGVGILFTRLSSEQFRNPLPPTEDPSVLGAAGFGQPQGSQGQYQ

>Agam_AGAP004657

MEEYAREPCPYRIVDDCGGAFAMGCIGGGVFQAIKGFRNAPSGFNRRLLGSLTAIKSRSP

IIAGNFAVWGGMFSTIDCTLVHFRKKEDPWNSIISGAATGGILAARNGVPAMIGSAVIGG

VLLALIEGVGIMFTRISAEQFRNPTPPSDDPSVLGDPNQQQMSSSAPSTFAFGQSGQNYQ

>Cpip_CPIJ003898

MEEYAREPCPYRIVDDCGGAFAMGCIGGGVFQAIKGFRNAPSGFSRRMVGSLTAIKARSP

IIAGNFAVWGGMFSTIDCTLVHFRKKEDPWNSIISGAATGGILAARNGVGAMAGSAIIGG

VLLALIEGVGILFTRLSAEQFRSQPIVEDPSALGDPTQNAASSSGASSTMPFGFGKDKGI

G

>Tcas_XP_971201

MEEYTREPCPWRIVDDCGGAFTMGLIGGGVFQSIKGFRNAPSGFNRRFVGSLAAIKQRSPIIAGNFAVWGGMFSTIDCALIHIRKKEDPWNSIISGAATGGILAARNGLPAMAGSAFIGGVLLALIEGVGILFTRLSAEQFQPQMPPMEDPSQLRGQQNGGYSFQ

>Amel_XP_003249498

MEEYAREPCPWRIIDDCGGAFTMGAIGGAVFQTIKGFRNAPSGINKRVLGSLTAIKQKSPIIAGNFALWGGMFSTIDCTLVHLRKKEDPWNSIISGAATGGILAARNGLPAMAGSAIIGGVLLALIEGVGIFITRLSAEQFKPPSFTEDANPLRQQGHPS

>Pcoq_MNCL01000012.1

MEEYAREPCPYRIVDDCGGAFAMGCIGGGVFQAIKGFRNAPAGFSKR----MARIKSDSSMEEYAREPCPYRIVDDCGGAFAMGCIGGGVFQAIKGFRNAPAGFSKR----MARIKSDSSXGSLSAIKTRSPIIAGNFAVWGGMFSTIDCTLVHIRKKEDPWNSIISGAATGGILAARNXVLLALIEGVGILFTRLSAEQFRNPMPPTEDPSVLGDANQGF

>Mdes_AEGA01006707.1

MEEYAREPCPYRIVDDCGGAFAMGCIGGGVFQAIKGFRNAPSGFSRRMVGXXXGSLAAIKQRSPIIAGNFAVWGGMFSTIDCTLVHFRKKEDPWNSIISGAATGGILAARNGXXXGVPAMAGSAVIGGVLLALIEGVGILFTRLSAEQFKNPMPPTEXXXGVPAMAGSAVIGGVLLALIEGVGILFTRLSAEQFKNPMPPTE

>Cnas_XP_031623587

MEEYAREPCPYRIVDDCGGAFAMGCIGGGVFQAIKGFRNAPSGFSRRMLGSLLAVKHRSPIIAGNFAVWGGMFSTIDCTLVHFRKKEDPWNSIISGAATGGILAARNGVPAMAGSALIGGVLLALIEGVGILFTRLSADQFRNPLPPTEDPSVLGDATQNNNNSNSAGSFFGLGQSTQYQ

>Cnas_VYII01000687_2

MEENECETCPYRIVGDCCAAFTTGCIGGGIYQMMKGFRNAPSGFQRRMVXAIKHRSPTIAGNFAIWGCLFSATECTLVHYRRKEDQWNSTISGATTLGILSARNGMKIYQXSQNISGVPAMVGSAVFGGVLLASIECIEMWFVR

>Smos_VUAH01000010.1

MEEYAREPCPYRIVDDCGGAFAMGCIGGGVFQAIKGFRNAPSGFSRRMVXFTPSQLGSLSAIKQRSPIIAGNFAVWGGMFSTIDCTLVHFRKKEDPWNSIISGAATGGILAARNGIPAMAGSAVIGGVLLALIEGVGILFTRLSADQFKNPLPPTEDPAALGDPSQQQNSSNSGAGFFGLSQPTQYQ
